# Comparison of bioactive material failure rates in vital pulp treatment of permanent matured teeth – a systematic review and network meta-analysis

**DOI:** 10.1038/s41598-024-69367-7

**Published:** 2024-08-08

**Authors:** Péter Komora, Orsolya Vámos, Noémi Gede, Péter Hegyi, Kata Kelemen, Adél Galvács, Gábor Varga, Beáta Kerémi, János Vág

**Affiliations:** 1 Department of Restorative Dentistry and Endodontics, Semmelweis University, 1088 Budapest Szentkiralyi Utca 47, Budapest, Hungary; 2 Centre for Translational Medicine, Semmelweis University, Budapest, Hungary; 3 Department of Prosthodontics, Semmelweis University, Budapest, Hungary; 4 Institute of Pancreatic Diseases, Semmelweis University, Budapest, Hungary; 5 Institute for Translational Medicine, Medical School, University of Pécs, Pécs, Hungary; 6 Department of Oral Biology, Semmelweis University, Budapest, Hungary

**Keywords:** Tricalcium silicate, Mineral trioxide aggregate, Calcium hydroxide, Network meta-analysis, Biocompatible materials, Dentistry, Endodontics, Pulp conservation, Pulpitis

## Abstract

Mineral Trioxide Aggregate (MTA) is the gold standard for vital pulp treatment (VPT), but its superiority over novel calcium silicate-based cements in permanent teeth lacks systematic evidence. This study aimed to compare the efficacy of these materials in VPT through a network meta-analysis. A systematic search was conducted in MEDLINE, EMBASE, Cochrane Library, and Web of Science until January 20, 2024. The inclusion criteria were randomized controlled trials involving VPT with biomaterials and reversible or irreversible pulpitis diagnoses in mature permanent teeth. The primary outcome was the odds ratio (OR) of failure rates with 95% confidence intervals. In the 21 eligible trials, failure rates were significantly higher with calcium-hydroxide than MTA at six (OR 2.26 [1.52-3.36]), 12 (OR 2.53 [1.76-3.62]), and 24 months (OR 2.46 [1.60-3.79]). Failure rates for Totalfill at six (OR 1.19 [0.55-2.58]) and 12 months (OR 1.43 [0.71-2.92]), and Biodentine at six (OR 1.09 [0.66-1.78]), 12 (OR 1.21 [0.74-1.96]), and 24 months (OR 1.47 [0.81-2.68]) were not significantly different from MTA. The results were similar in the direct pulp capping subgroup, whereas, in the partial and full pulpotomy subgroup, there was not enough evidence to achieve significant differences. MTA, Biodentine, and Totalfill are the most efficient materials for VPT. However, calcium-hydroxide-based materials are not recommended in VPT.

## Introduction

Vital pulp treatment (VPT, alternatively, the vital pulp therapy), such as pulp capping, partial pulpotomy, or full pulpotomy, offers a faster, less invasive, and technically simpler alternative to root canal treatment for the treatment of deep carious lesions and exposed pulp^1^. VPT could be at least as successful as root canal treatment^2–4^.

VPT aims to preserve pulp vitality and function^5^; therefore, the innate immune defense mechanism of the pulp is retained^6^. In contrast, loss of pulp vitality results in impaired receptor function, such as the sensation of biting forces^7,8^. The pulp tissue in a tooth acts as a shock absorber during biting and chewing^9^ and by influencing proprioceptive sensation, potentially providing a higher tolerance to mechanical stress and reducing the risk of tooth fracture^8,10,11^.

A key factor in the success of VPT is the bioactive material applied to the pulp tissue^1,12,13^. Mineral Trioxide Aggregate (MTA) is the gold standard material for VPT^12,13^. Its biocompatibility has been demonstrated in vitro^14,15^, in vivo^16,17^, and in case report^18^. The pH of MTA is high during the setting process; therefore, it has an excellent antimicrobial effect^19,20^. Clinical studies^21–23^ have reported a high success rate of MTA in VPT. However, the clinical application and mixing of MTA are not straightforward, but maintaining a proper mixing ratio of the components affects the physical characteristics of MTA^20^. In addition, MTA has a long setting time^24,25^ and needs excess water for the complete setting process^20^. Although the newer version of MTA has a reduced setting time, Biodentine is still the fastest^26^. Nevertheless, MTA may cause tooth discoloration^27–29^.

Over the past 10–15 years, several bioactive materials have been developed for VPT to replace MTA. For example, Biodentine has a faster setting time and causes less discoloration on the tooth^30^. TheraCalLC is a light-curing resin-modified calcium silicate-based material^31^ with a dentin bridge formation similar to MTA^32^. However, TheraCalLC has shown higher in vitro cytotoxicity than MTA or Biodentine^33^. Totalfill is a premixed calcium silicate-based root repair material with an excellent clinical success rate^34^. The calcium-enriched mixture (CEM) contains calcium oxide (CaO), sulfur trioxide (SO_3_), phosphorous pentoxide (P_2_O_5_), and silicon dioxide (SiO_2_)^35^. CEM has good sealing ability and antimicrobial effects^36,37^.

Some previous pairwise meta-analyses have compared the MTA with the Ca(OH)_2_. The MTA was found to be superior to Ca(OH)_2_ in direct pulp capping of permanent teeth after three months^38^ and at 12 and 24 months^39^. However, after indirect pulp capping, the difference was not significant at 6 and 12 months^40^. However, less evidence is available for pulpotomy. A meta-analysis based on cohort studies^41^ found no significant superiority of CEM or MTA over Ca(OH)_2_ after full pulpotomy in matured permanent teeth. More recently, a meta-analysis of randomized controlled trials^42^ found significant superiority of MTA over Ca(OH)_2_ in direct pulp capping and pulpotomy.

In addition, previous pairwise meta-analyses have compared the MTA with new biomaterials. The efficacy of Biodentin was comparable to that of MTA in direct pulp capping^39^. Similarly, calcium silicate-based materials were similar to MTA in other VPT modalities^43^. However, the biomaterials were combined into a single group. Whether the new biomaterials had a distinct effect on the success rate based on the VPT modalities and follow-up period remains to be answered. Therefore, a stratified network meta-analysis is proposed to compare MTA, Ca(OH)_2_, and different calcium silicate-based materials considering the follow-up period and using only RCTs in matured permanent teeth.

The primary objective of this study was to compare the clinical failure rate of calcium-hydroxide-based and contemporary bioactive materials in VPT of permanent teeth to MTA. The secondary aim was to perform a subgroup analysis based on intervention types by comparing the direct and indirect pulp capping and partial and full pulpotomy. This study aimed to rank these bioactive materials by network meta-analysis^44^.

## Materials and methods

### Protocol and registration

This systematic review and meta-analysis were conducted according to the Preferred Reporting Items for Systematic Reviews and Network Meta-Analyses (PRISMA-NMA) guidelines^45,46^. It was registered in the Prospectively Registered Systematic Reviews (PROSPERO) database (CRD42022375573).

### Eligibility criteria

The PICOS framework was used. The clinical question was: Is there a difference in failure rate between different bioactive materials and MTA in the vital pulp treatment of permanent teeth?

The PICOS details were as follows:

P: Mature permanent teeth with vital pulp therapy.

I: Bioactive materials (BiodentineTM, Ca(OH)_2_, CEM, Calcium Silicate-based Sealer, Calcium silicate-based materials).

C: Mineral Trioxide Aggregate (MTA).

O: Failure rate.

S: Randomized controlled trials.

### Inclusion criteria for studies

Randomized controlled trials were included. Studies with preoperative diagnosis, a description of the clinical procedure, and biomaterials. Studies with the initial diagnosis of reversible or irreversible pulpitis or Wolters classification-based pulpitis^47^. The failure of VPT was defined as having clinical symptoms, pain, sensitivity to cold and percussion tests, and/or signs of periapical inflammation. The number of study participants in each follow-up period is defined. The minimum follow-up period was six months.

### Exclusion criteria for studies

The exclusion criteria of studies are the following: case reports, two-armed interventional, cohort, and case-controlled studies, meta-analyses, reviews, animal studies, conference abstracts, non-English written papers, and the study population involved primary dentition, immature permanent teeth, and trauma cases.

### Information sources and search strategy

An electronic literature search was conducted using the following databases: MEDLINE (PubMed), EMBASE, Cochrane Library, and Web of Science. A citation chase was performed (https://www.rayyan.ai/). The following search key was used: (vital pulp therapy OR vital-pulp therapy OR direct pulp capping OR direct pulp-capping OR indirect pulp capping OR indirect pulp-capping OR partial pulpotomy OR partial-pulpotomy OR full pulpotomy OR full-pulpotomy OR pulp capping OR pulp-capping) AND (Biodentine OR CEM OR Calcium Silicate Sealer OR Calcium-silicate based materials OR calcium silicate-based material OR calcium hydroxide OR MTA OR pulp capping material* OR calcium silicate OR calcium silicate materials OR biomaterial*) AND (Adult* OR mature tooth OR mature teeth OR permanent teeth OR permanent tooth OR permanent OR mature).

The systematic search was completed on January 20, 2024. All articles were imported into EndNote 20 for duplicate removal. The articles were arranged alphabetically for manual screening. First, automatic duplicate removal was performed in EndNote 20, followed by manual duplicate removal. Then, the database was uploaded to the Rayyan system (https://www.rayyan.ai/).

### Selection process

The entire selection process was conducted simultaneously by two independent observers (P.K. and O.V.), with each observer evaluating articles based on their title, abstract, and full text. The first screening involved the titles and the abstracts of the articles. Then, a full-text selection was completed for all studies that met the inclusion criteria or when there was insufficient information in the abstract to make a proper decision. Conflicts were resolved through discussion with the two reviewers. The same two independent researchers reviewed the full texts for quality and inclusion in the network meta-analysis. A third author (K.K.) was also involved in case of disagreement.

### Data collection/extraction process

Two independent observers collected data, P.K. and O.V. No automatic tools were used for data extraction. First, the data of some articles that matched the inclusion criteria was extracted into an MS Excel sheet form. The statistician verified the form first. Studies declared that only the percentage of failed and successful cases without absolute numbers were excluded from the meta-analysis.

### Data items/Effect measure

The verified MS Excel form was used for data extraction of all included studies, comprising the following information: publication information (author/year), study type, demographics of study participants, pulpal diagnosis, type of vital pulp treatment, materials applied, follow-up times, and numbers lost to follow-up. The primary outcomes were vital pulp treatment failure rates after six months, 12 months, and 24 months. The effect measure was the odds ratio (OR) with 95% confidence intervals^48^. If the tested material had an odds ratio of higher than one, indicating a higher failure rate for the tested material than for the MTA group.

Some studies^49–53^ did not calculate the previously lost unsuccessful cases for every follow-up. Therefore, the authors of this study added the number of unsuccessful lost cases to the number of participants at the subsequent follow-up for the meta-analysis to obtain the actual failure rate.

### Risk of bias (RoB) assessment

According to the Cochrane Collaboration, the risk of bias in the randomized clinical trials was analyzed by the Risk of Bias 2 tool (https://methods.cochrane.org/bias/resources/rob-2-revised-cochrane-risk-bias-tool-randomized-trials). The Risk of Bias 2 tool consists of five domains: randomization process (D1), deviations from intended interventions (D2), missing outcome data (D3), measurement of outcome (D4), selection of reported results (D5). Each domain can have three different results: low risk, some concern, and high risk. The worst domain result will set the overall bias of the particular study.

### Synthesis methods

First, pooled analysis was performed separately for data from all treatment modalities (including indirect and direct pulp capping and partial and full pulpotomy) at each follow-up interval (6, 12, and 24 months). In the second step, subgroup analysis was conducted separately for each treatment modality (indirect pulp capping, direct pulp capping, partial pulpotomy, and full pulpotomy) at each follow-up interval, provided a minimum of three eligible studies were available for each subgroup.

A frequentist method was used to perform the network meta-analyses (NMAs). As we assumed considerable between-study heterogeneity due to the nature of medical treatments and conditions, a random-effects model was used to pool effect sizes (ORs) with a 95% confidence interval (95% CI). The reason for using this method for dichotomous data is zero events in the studies. This method handles zero events with correction. The network plot indicates the strength of the comparisons between materials by depicting the number of patients and studies.

The three follow-up periods were analyzed in a stratified manner. Forest plots were used to examine the consistency of the relationship between direct, indirect, and network point estimates. Consistency means that the relative effect of a comparison based on direct evidence does not differ from the one based on indirect evidence^54^. The evidence plot shows direct and indirect estimation ratios in the network meta-analysis. Funnel plots were constructed for all outcomes, and Egger's tests were performed to assess the small-study effect. P-scores rank treatments, which measure the certainty that one treatment is better than another treatment, and are averaged over all competing treatments. P-score ranges from 0 to 1, with higher values indicating higher ranks. Calculations were performed using the R package (version 4.1.1), Netmeta (version 2.7–0), and Pairwise (version 0.6.0–0). Netmeta could estimate network meta-analysis models within a frequentist framework and derive from graph-theoretical techniques initially developed for electrical networks^55^.

### Certainty assessment

The certainty assessment was evaluated according to CINeMA (Confidence in Network Meta-Analysis). CINeMA includes six domains: within-study bias, reporting bias, indirectness, imprecision, heterogeneity, and incoherence^56,57^. There are three levels of judgment for each domain (no concerns, some concerns, or major concerns). Domain judgments can be summarized into four confidence levels for each outcome: very low, low, moderate, or high.

CINeMA considers the agreement between the confidence and prediction intervals to assign a score for 'heterogeneity' for each NMA effect (i.e. pair of cements). Prediction intervals provide a range within which the true effect of a new study is likely to lie^57^. The imprecision and heterogeneity scores were based on an arbitrarily chosen odds ratio of 1.2, which was considered clinically important.

### Ethics approval statement and document

No ethical approval was required for this systematic review and network meta-analysis, as all data were published in peer-reviewed journals. Datasets used in this study can be found in the full-text articles included in systematic reviews and network meta-analyis.

## Results

### Study selection

**Figure 1:**
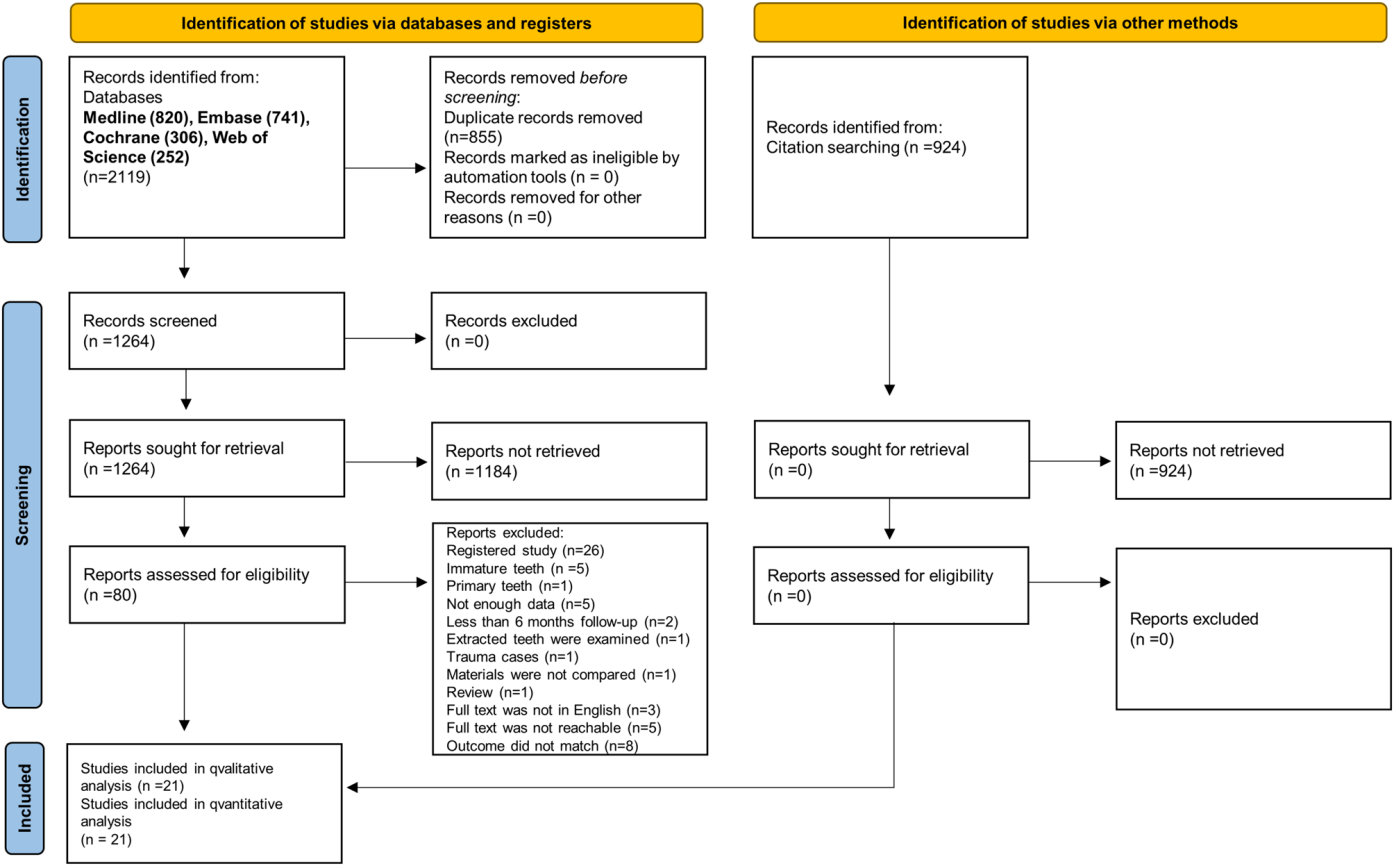
Prisma 2020 flow diagram of the screening and selection process.

The systematic search revealed 2119 records in MEDLINE (820), EMBASE (741), CENTRAL (306), and Web of Science (252). Automatic and manual removal of duplicates resulted in 1264 articles. After title and abstract selection, 80 eligible articles remained. Cohen's kappa was 0.96 between the two independent observers. After full-text reading, 21 articles were selected^4,34,49–53,58–71^. Cohen’s kappa of the full-text selection was 0.89 (Fig. 1). The excluded studies and the reason for exclusion are listed in Supplementary Table 1.

### Study characteristics

**Figure 2:**
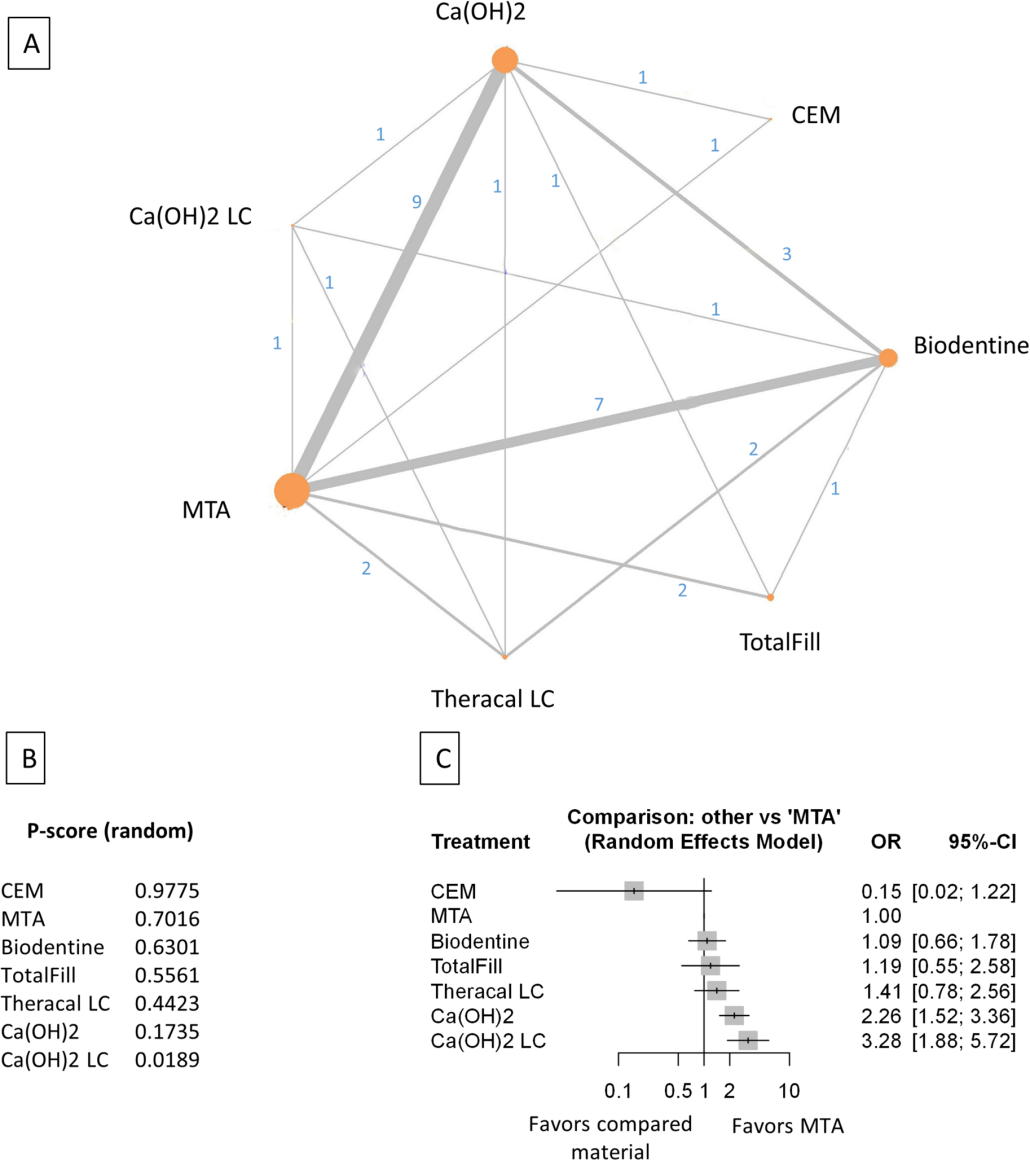
Comparing MTA and investigated bioactive materials at 6 months follow-up—pooled data, including all treatment modalities (**A**) Network-plot: An orange circle represents every bioactive material; the circle size represents the number of treated patients. The width of the lines and the numbers represent the number of studies where a direct comparison was made. (**B**) P-score: The p-score represents a ranking between the compared materials. (**C**) Outcome forest plot: The zero effect is the MTA vs. the other materials. The grey squares represent the odds ratio (OR), and the length of the black lines shows 95% (CI) confidence intervals.

**Figure 3:**
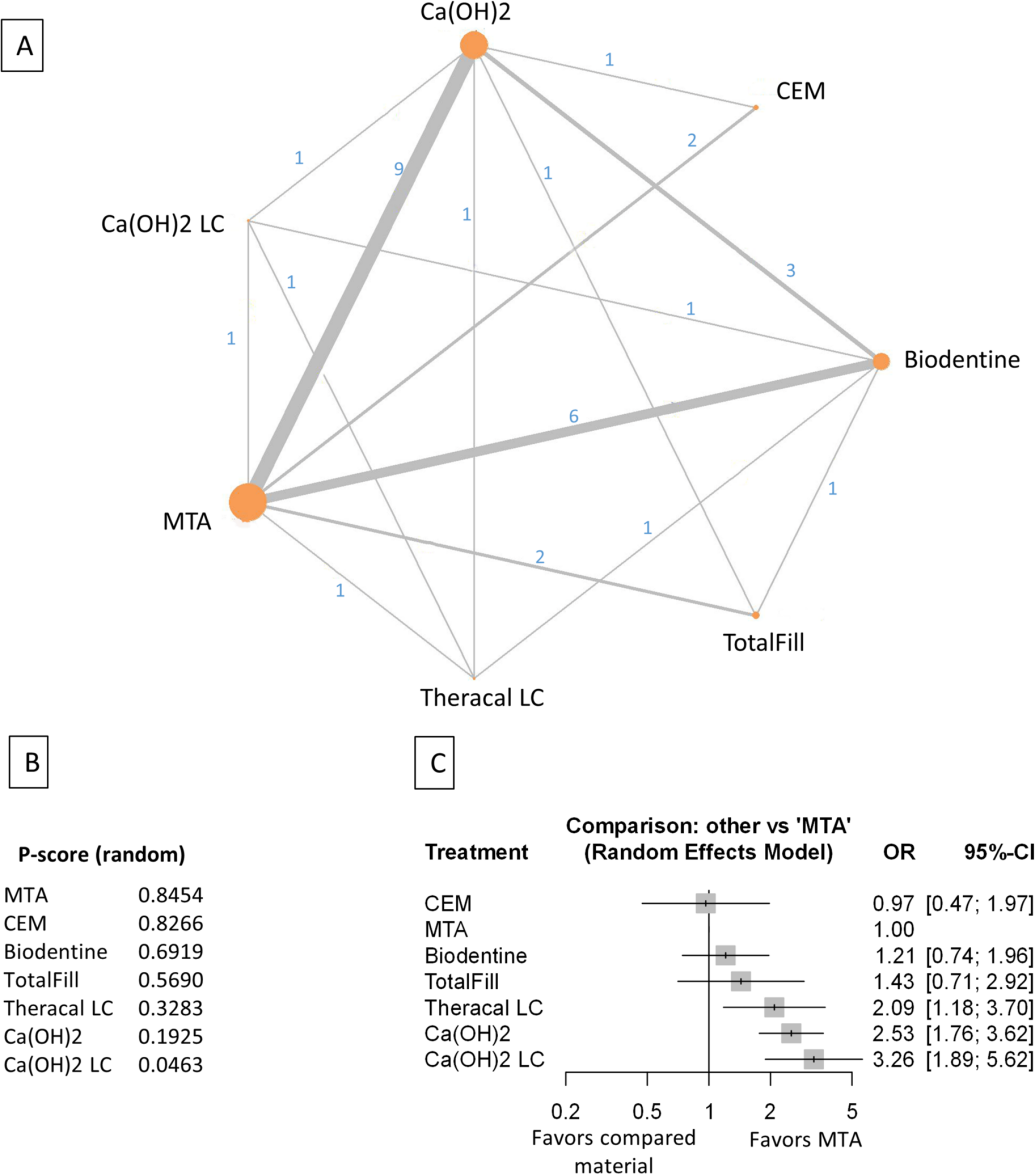
Comparing MTA and investigated bioactive materials at 12 months follow-up—pooled data, including all treatment modalities (**A**) Network-plot: An orange circle represents every bioactive material; the circle size represents the number of treated patients. The width of the lines and the numbers represent the number of studies where a direct comparison was made. (**B**) P-score: The p-score represents a ranking between the compared materials. (**C**) Outcome forest plot: The zero effect is the MTA vs. the other materials. The grey squares represent the odds ratio (OR), and the length of the black lines shows 95% (CI) confidence intervals.

**Figure 4:**
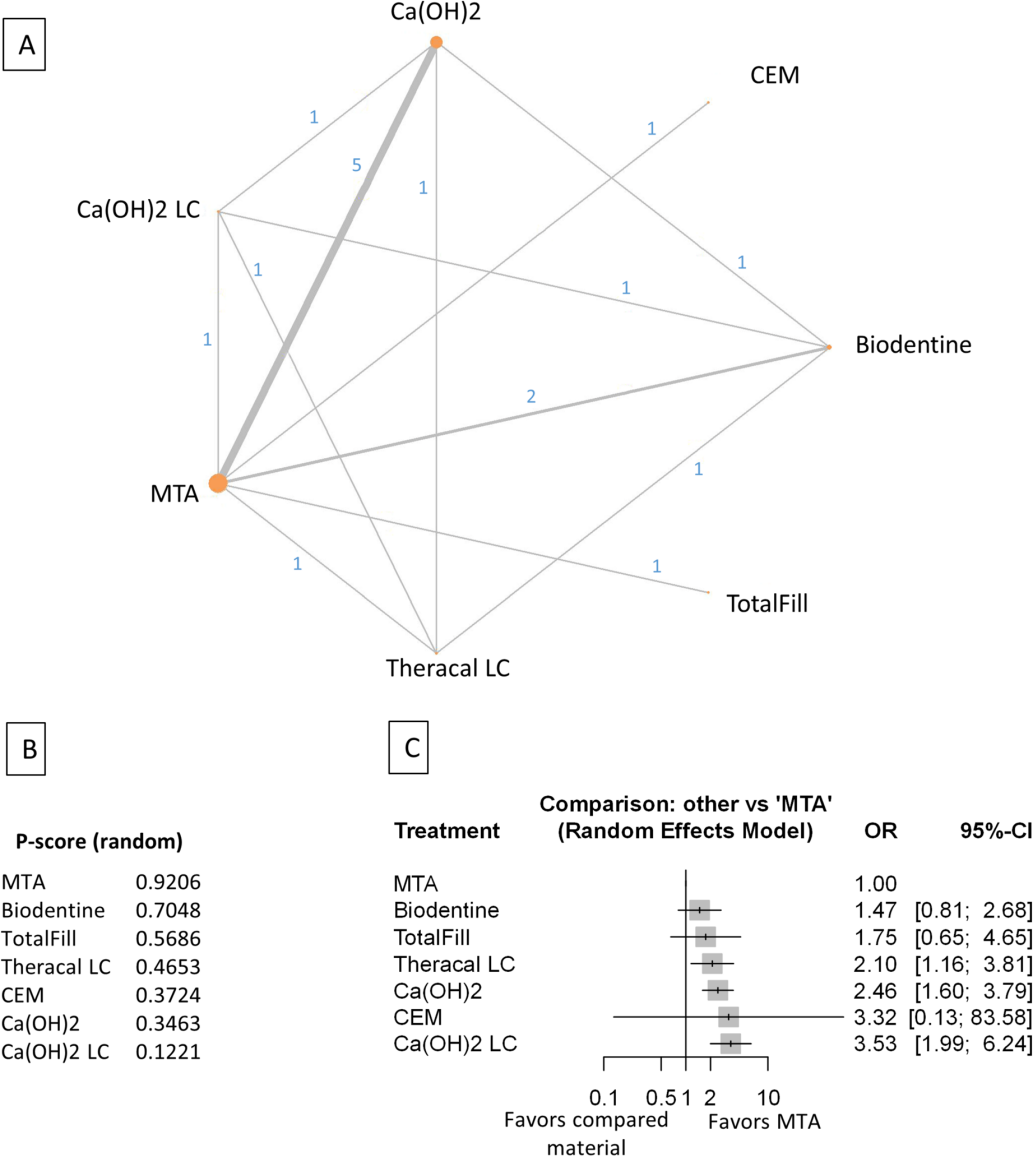
Comparing MTA and investigated bioactive materials at 24 months follow-up—pooled data, including all treatment modalities (**A**) Network-plot: An orange circle represents every bioactive material; the circle size represents the number of treated patients. The width of the lines and the numbers represent the number of studies where a direct comparison was made. (**B**) P-score: The p-score represents a ranking between the compared materials. (**C**) Outcome forest plot: The zero effect is the MTA vs. the other materials. The grey squares represent the odds ratio (OR), and the length of the black lines shows 95% (CI) confidence intervals.

**Figure 5:**
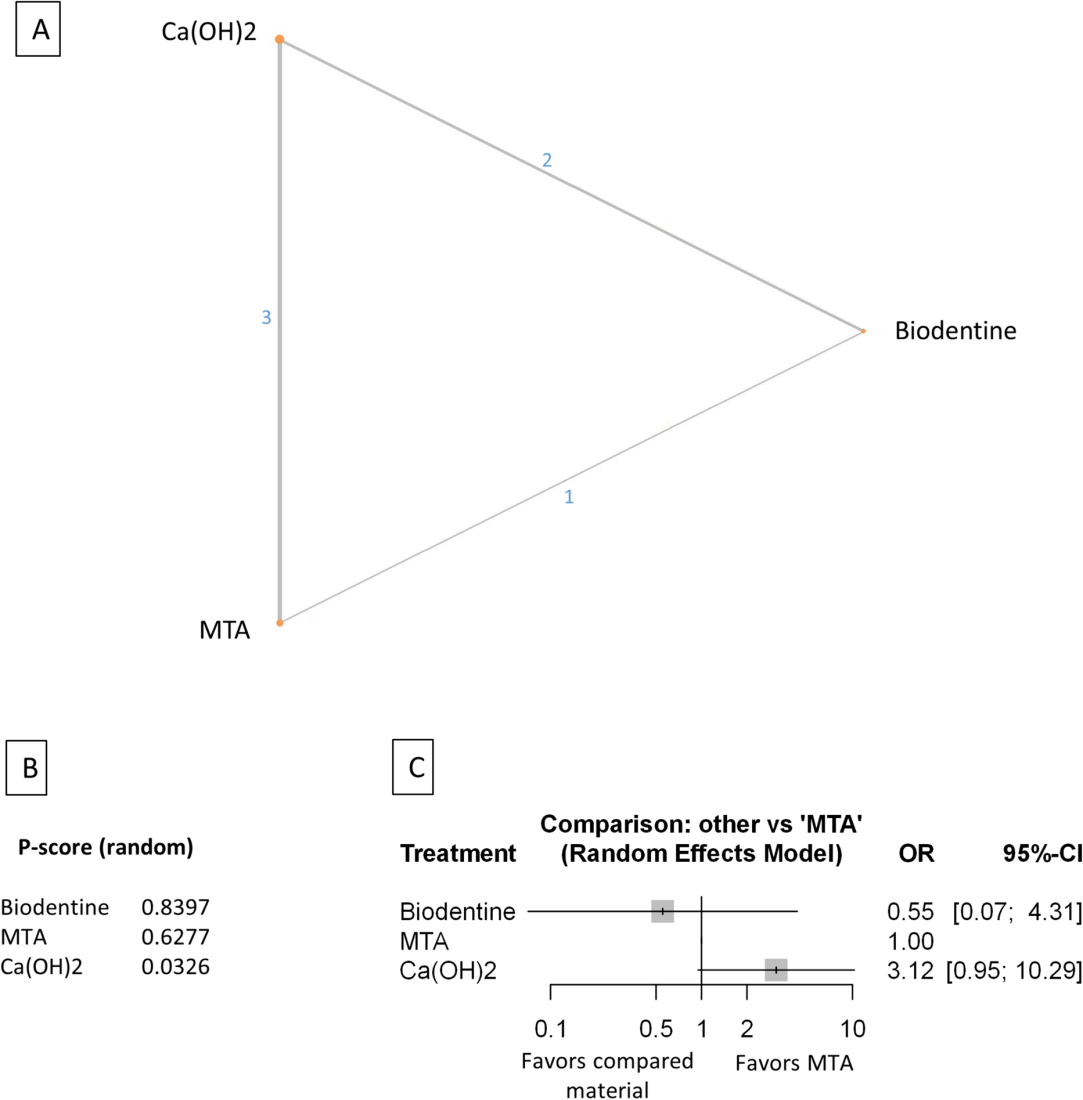
Comparing MTA and investigated bioactive materials at 6 months follow-up—indirect pulp capping (**A**) Network-plot: An orange circle represents every bioactive material; the circle size represents the number of treated patients. The width of the lines and the numbers represent the number of studies where a direct comparison was made. (**B**) P-score: The p-score represents a ranking between the compared materials. (**C**) Outcome forest plot: The zero effect is the MTA vs. the other materials. The grey squares represent the odds ratio (OR), and the length of the black lines shows 95% (CI) confidence intervals.

**Figure 6:**
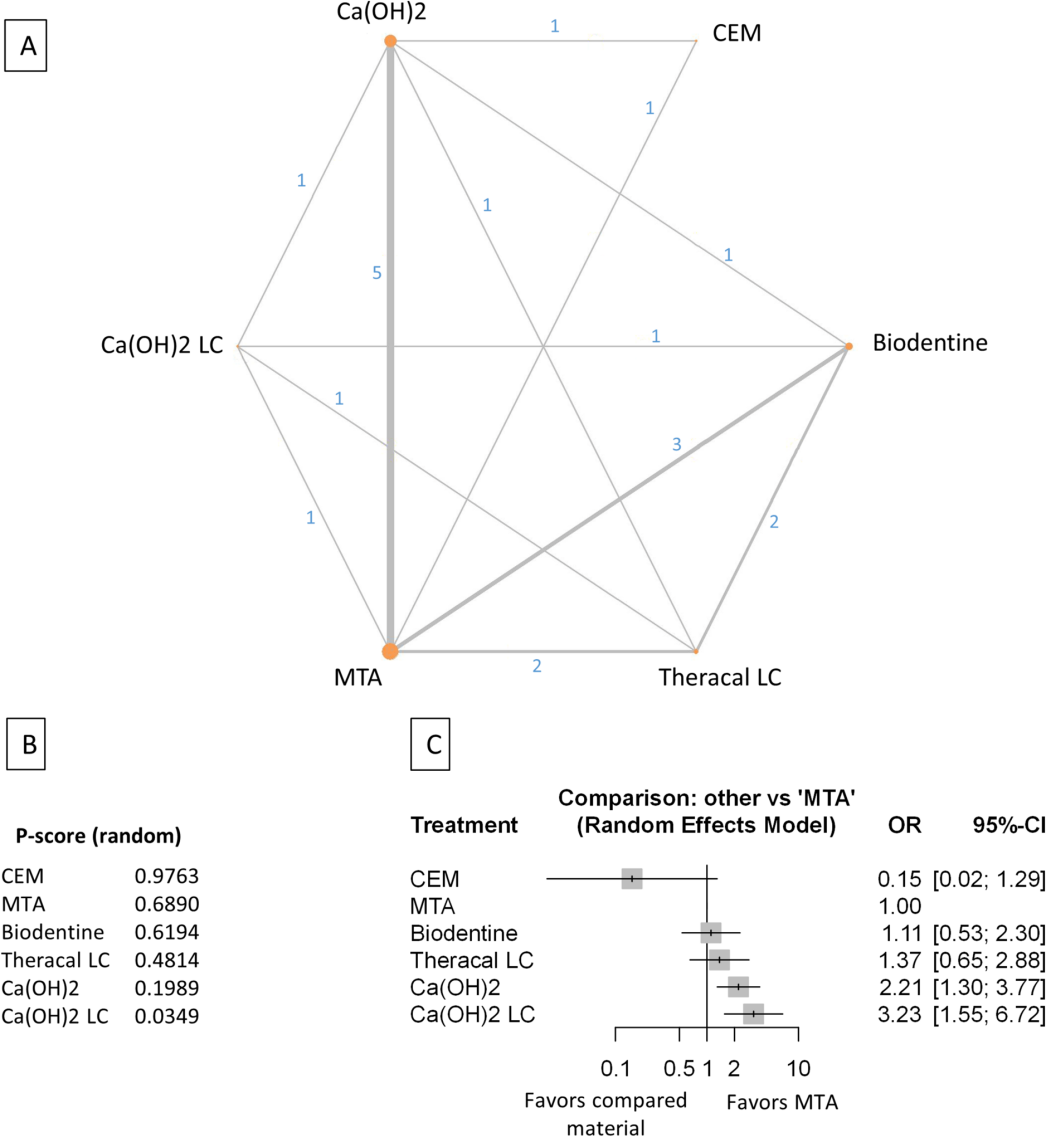
MTA and investigated bioactive materials at 6 months follow-up—direct pulp capping (**A**) Network-plot: An orange circle represents every bioactive material; the circle size represents the number of treated patients. The width of the lines and the numbers represent the number of studies where a direct comparison was made. (**B**) P-score: The p-score represents a ranking between the compared materials. (**C**) Outcome forest plot: The zero effect is the MTA vs. the other materials. The grey squares represent the odds ratio (OR), and the length of the black lines shows 95% (CI) confidence intervals.

**Figure 7:**
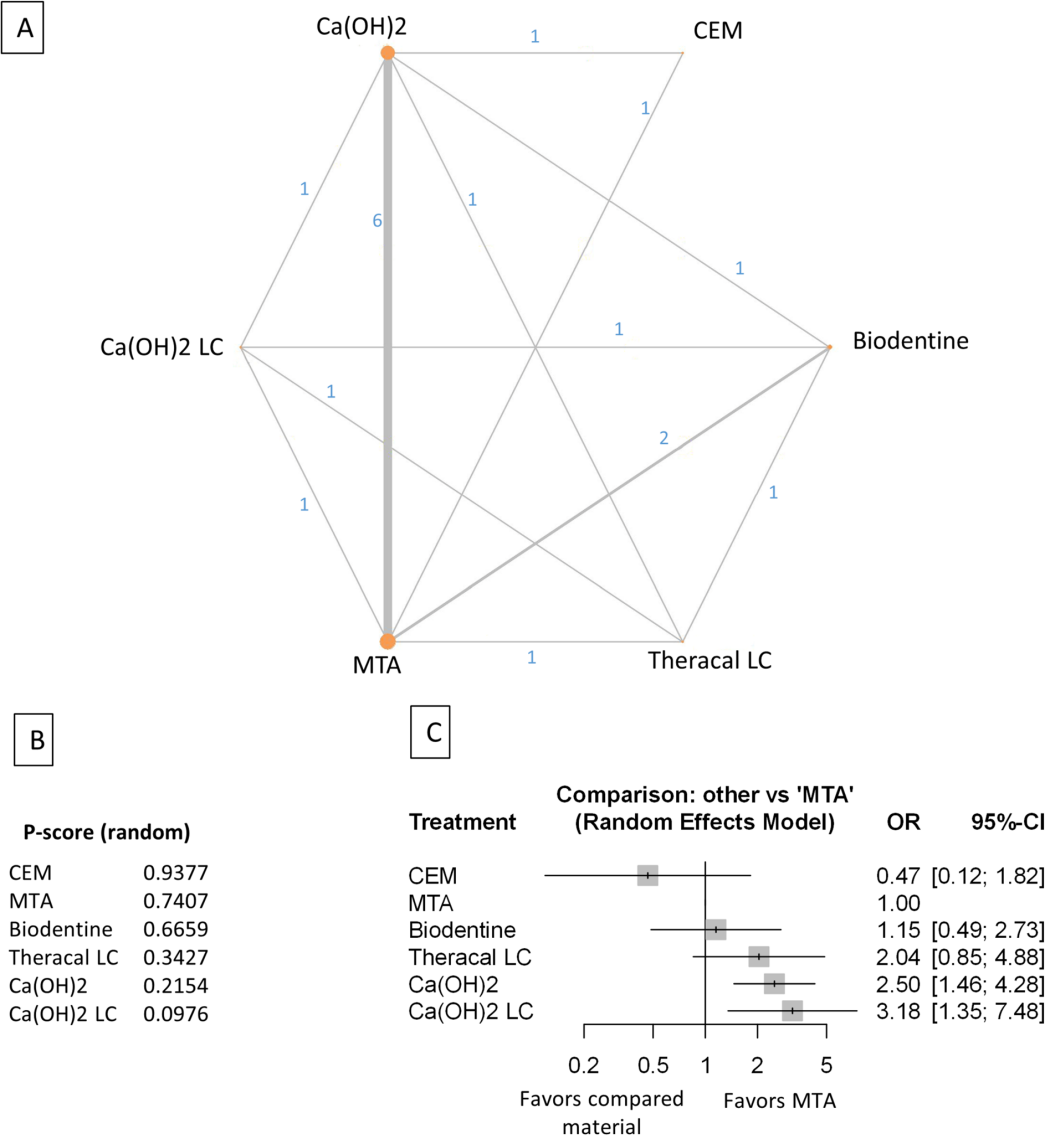
MTA and investigated bioactive materials at 12 months follow-up—direct pulp capping (**A**) Network-plot: An orange circle represents every bioactive material; the circle size represents the number of treated patients. The width of the lines and the numbers represent the number of studies where a direct comparison was made. (**B**) P-score: The p-score represents a ranking between the compared materials. (**C**) Outcome forest plot: The zero effect is the MTA vs. the other materials. The grey squares represent the odds ratio (OR), and the length of the black lines shows 95% (CI) confidence intervals.

**Figure 8:**
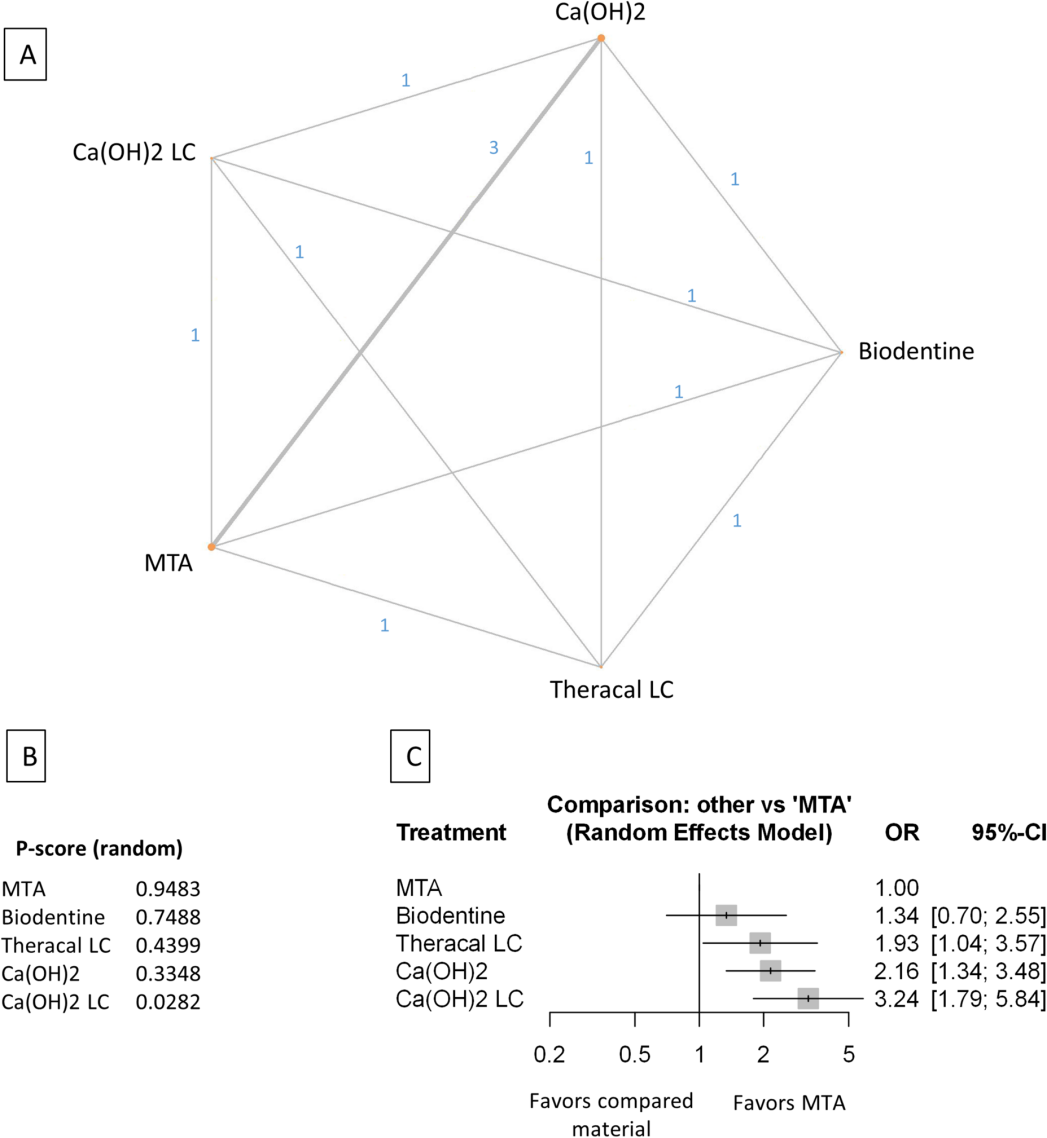
MTA and investigated bioactive materials at 24 months follow-up—direct pulp capping (**A**) Network-plot: An orange circle represents every bioactive material; the circle size represents the number of treated patients. The width of the lines and the numbers represent the number of studies where a direct comparison was made. (**B**) P-score: The p-score represents a ranking between the compared materials. (**C**) Outcome forest plot: The zero effect is the MTA vs. the other materials. The grey squares represent the odds ratio (OR), and the length of the black lines shows 95% (CI) confidence intervals.

**Figure 9:**
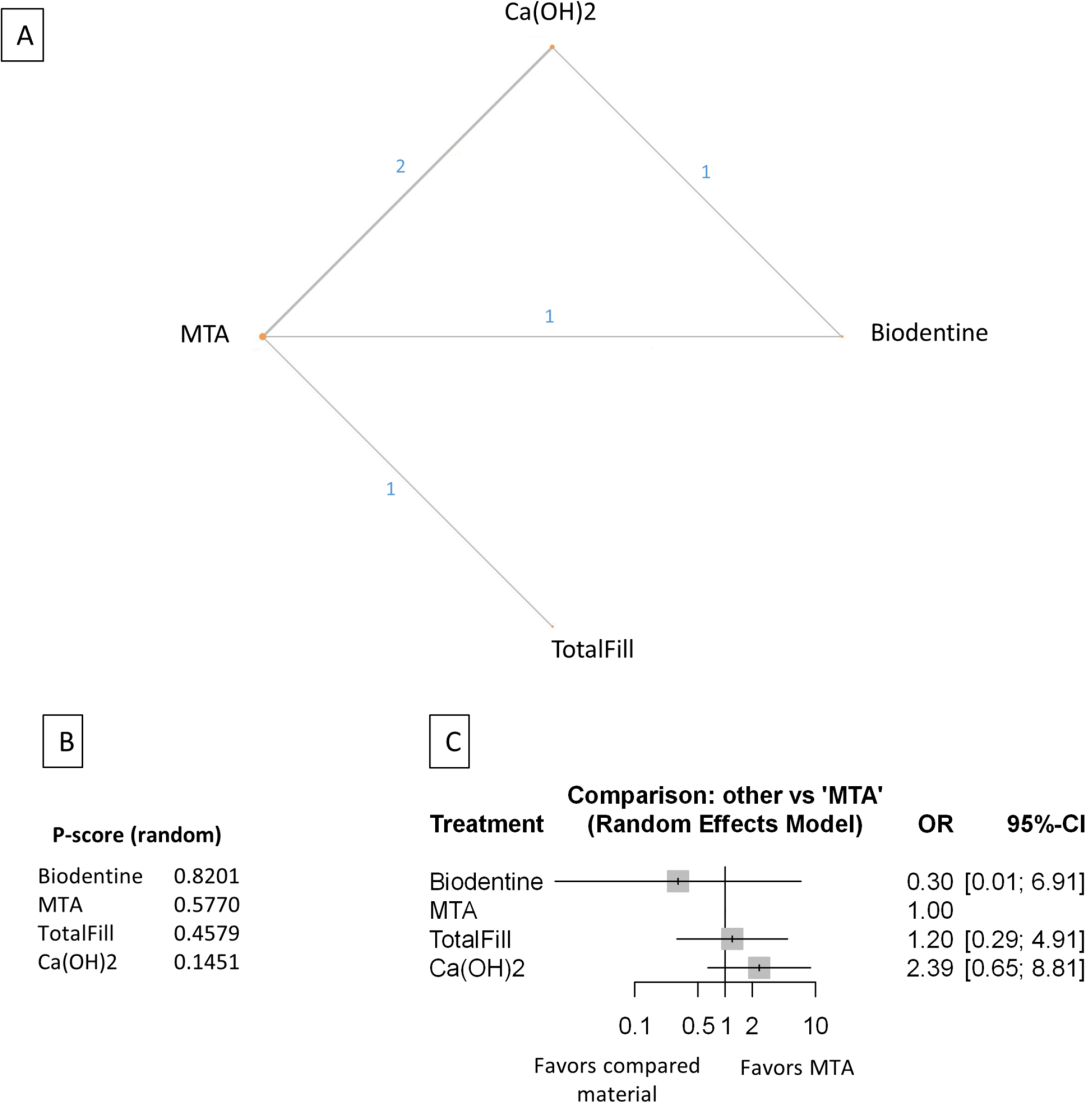
Comparing MTA and investigated bioactive materials at 12 months follow-up—partial pulpotomy (**A**) Network-plot: An orange circle represents every bioactive material; the circle size represents the number of treated patients. The width of the lines and the numbers represent the number of studies where a direct comparison was made. (**B**) P-score: The p-score represents a ranking between the compared materials. (**C**) Outcome forest plot: The zero effect is the MTA vs. the other materials. The grey squares represent the odds ratio (OR), and the length of the black lines shows 95% (CI) confidence intervals.

**Figure 10:**
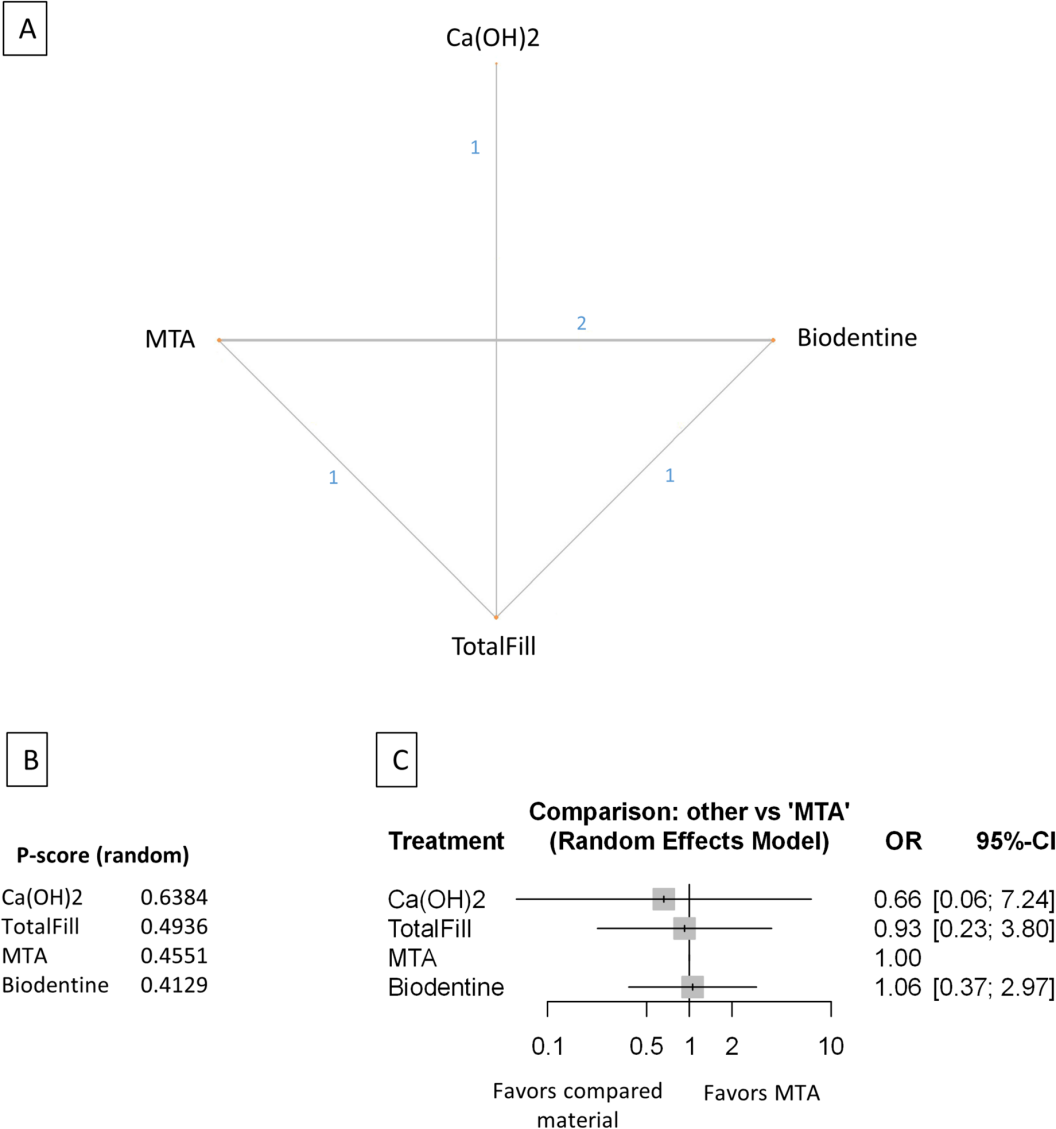
Comparing MTA and investigated bioactive materials at 6 months follow-up—full pulpotomy (**A**) Network-plot: An orange circle represents every bioactive material; the circle size represents the number of treated patients. The width of the lines and the numbers represent the number of studies where a direct comparison was made. (**B**) P-score: The p-score represents a ranking between the compared materials. (**C**) Outcome forest plot: The zero effect is the MTA vs. the other materials. The grey squares represent the odds ratio (OR), and the length of the black lines shows 95% (CI) confidence intervals.

**Figure 11:**
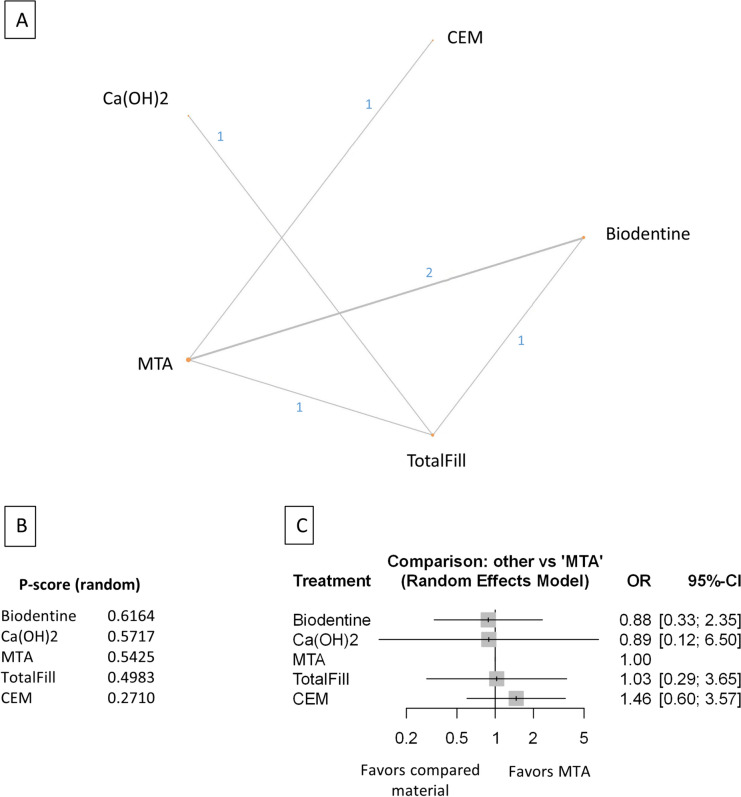
Comparing MTA and investigated bioactive materials at 12 months follow-up—full pulpotomy (**A**) Network-plot: An orange circle represents every bioactive material; the circle size represents the number of treated patients. The width of the lines and the numbers represent the number of studies where a direct comparison was made. (**B**) P-score: The p-score represents a ranking between the compared materials. (**C**) Outcome forest plot: The zero effect is the MTA vs. the other materials. The grey squares represent the odds ratio (OR), and the length of the black lines shows 95% (CI) confidence intervals.

Baseline characteristics of the 21 included studies are presented in (Table 1). The number of treated and lost patients and the successful and unsuccessful cases in every follow-up period, funding source, and conflict of interest were recorded. The network plot (Figs. 2A, 3A, 4A, 5A, 6A, 7A,8A, 9A, 10A, 11A) indicates the number of direct comparisons between materials for the 6, 12, and 24 month follow-up periods. The description of the used materials is presented in Supplementary Table 2.

**Table 1:** Baseline characteristics of the included studies.

Publication data	DOI	Study design	Type of therapy	Diagnosis	Material	Investigated follow-up periods	6 months follow-up			12 months follow-up			24 months follow-up			Funding sources	Conflict of interest
							Sample size	Number of success cases	Number of failed cases	Sample size	Number of success cases	Number of failed cases	Sample size	Number of success cases	Number of failed cases		
Taha et al. 2022	10.1111/iej.13707	rct	Full pulpotomy	Revesible and irreversible	MTA (ProRoot; Dentsply, Tulsa, OK)	6, 12 months	41	38	3	49	45	4				This study was supported by Deanship of Research at Jordan University of Science and Technology. Research grant number 2017/503	The authors deny any conflicts of interest related to this study
					Biodentine (Septodont)		33	30	3	45	42	3					
					TotalFill (FKG Dentaire SA)		54	50	4	62	57	5					
Awawdeh et al. 2018	10.1016/j.joen.2018.08.004	rct	Direct pulp capping and full pulpotomy	Revesible and irreversible pulpitis	MTA (Angelus)	6, 12 and 24 months	31	29	2	30	28	2	29	27	2	Supported by the deanship of research at Jordan University of Science and Technology, Irbid, Jordan (grant no. 154/2013)	The authors deny any conflicts of interest related to this study
					Biodentine (Septodont)		29	27	2	27	24	3	27	24	3		
Taha and Khazali 2017	10.1016/j.joen.2017.03.033	rct	Partial pulpotomy	Irreversible pulpitis	MTA (ProRoot; Dentsply, Tulsa, OK)	6, 12 and 24 months	25	21	4	24	20	4	26	22	4	Supported by deanship of research at Jordan University of Science and Technology (grant no. 218/2014)	The authors deny any conflicts of interest related to this study
					Ca(OH)2 (Dycal; Dentsply Caulk, Milford, DE)		21	13	8	20	11	9	23	10	13		
Mente et al. 2010	10.1016/j.joen.2010.02.024	rct	Direct pulp capping	Reversible pulpitis, accidental opening	MTA (ProRoot; Dentsply-Maillefer)	12, 24 months				29	24	5	26	18	8	N/A / Not mentioned	N/A / Not mentioned
					Ca(OH)2 (Hypocal SN)					20	15	5	20	11	9		
Asgary and Eghbal 2013	10.3109/00,016,357.2011.654251	rct	Full pulpotomy	Irreversible pulpitis	CEM (CEM cement; BioniqueDent, Tehran, Iran)	12 months				167	155	12				This work was supported by the Iranian Ministry of Health and Medical Education (Health Deputy, Oral Health Office) and Shahid Beheshti University of Medical Sciences [grant number p/25/17/1/tm/103]	The authors alone are responsible for the content and writing of the paper. Prof. Saeed Asgary is the inventor of calcium enriched mixture (CEM) cement (Endodontic Filling Material;USA, 7,942,961, 2011 May 17)
					MTA ( ProRoot, Dentsply, Tulsa Dental, Tulsa, OK)					179	170	9					
Leye Benoist et al. 2012	10.1111/j.1875-595X.2011.00084.x	rct	Indirect pulp capping	Reversible pulpitis	Ca(OH)2 (Dycal Ivory, Dentsply Caulk, Dentsply, L.D. Caulk, Milford, DE, USA)	6 months	26	22	4							The authors thank Dr Papa Ibrahima Ngom, Associate Professor in Orthodontics at University Cheikh Anta Diop, Dakar, Senegal, who performed statistical analysis, and Roland Arsan for the gracious provision of products that enabled this study to be conducted	This study was not financed by any company or manufacturer and has no commercial aim
					MTA (ProRoot; Dentsply ⁄ Tulsa Dental, Tulsa, OK, USA)		29	27	2								
Hilton et al. 2013	10.1177/0,022,034,513,484,336	rct	Direct pulp capping	Not defined, but spontaneous or lingering pain was excluded	Ca(OH)2 (Life, Kerr, Orange, CA, USA)	6, 12 and 24 months	143	129	14	117	91	26	53	21	32	This work was supported in part by NIH/NIDCR Grants U01 DE016750 and U01 DE016752	The authors declare no potential conflicts of interest with respect to the authorship and/or publication of this article
					MTA (Pro Root, Tulsa Dental, Tulsa,OK, USA)		160	151	9	134	122	12	42	23	19		
Koc Vural et al. 2017	10.2341/16–110-C	rct	Indirect pulp capping	Reversible pulpitis	Ca(OH)2 (Dycal, Dentsply/Caulk, Dentsply International Inc, Milford, DE, USA)	6, 12 and 24 months	49	47	2	49	45	4	49	45	4	This study was supported by the Hacettepe University Scientific Research Project Coordination Unit (project number 012D09201)	The authors of this article certify that they have no proprietary, financial, or other personal interest of any nature or kind in any product, service, and/or company presented in this article
					MTA (ProRoot Dentsply Tulsa Dental, Johnson City, TN, USA)		51	51	0	51	50	1	51	49	2		
Suhag et al. 2019	10.1016/j.joen.2019.02.025	rct	Direct pulp Capping	Reversible pulpitis	Ca(OH)2 (Prevest)	6, 12 months	30	21	9	29	20	9				N/A / Not mentioned	The authors deny any conflicts of interest related to this study
					MTA (ProRoot Dentsply Tulsa Dental, Tulsa, OK)		27	25	2	27	25	2					
Peskersoy et al. 2021	10.1016/j.jds.2020.08.016	rct	Direct pulp capping	Reversible pulpitis	Ca(OH)2 (Dycal, Dentsply-Sirona, Charlotte, NC, USA)	6, 12 and 24 months*	106	81	25	106	76	30	106	73	33	This study was funded by grant 2013-DIS-016 from the Ege University Hospital, Turkey	The authors have no conflicts of interest relevant to this article
					Ca(OH)2 Light cure (LC Calcihyd, Dr. Roberts’, Istanbul, Turkey)		105	69	36	105	67	38	105	64	41		
					Theracal LC (Bisco, Schaumburg, IL, USA)		105	85	20	105	77	28	105	76	29		
					Biodentine (Septodont,Saint-Maur-des-Fosse´s France)		105	88	17	105	84	21	105	83	22		
					MTA (BioMTA + ,Cerkamed, Stalowa Wola, Poland)		105	90	15	105	90	15	105	89	16		
Iyer et al. 2021	10.4103/jcd.jcd_71_21	rct	Direct pulp capping	Reversible pulpitis	Theracal LC (Bisco, USA)	6 months	27	25	2							None	There are no conflicts of interest
					Biodentine (Septodont, France)		27	24	3								
					MTA Plus (Prevest Ltd., Jammu, India)		26	24	2								
Ahlawat et al. 2022	10.4103/jpbs.jpbs_837_21	rct	Direct pulp capping	Pulp sensibility was tested, diagnose was not defined. Possible reversible pulpitis	MTA (manufacturer not defined)	6, 12 months	60	51	9	59	47	12				None	There are no conflicts of interest
					Biodentine (Septodont)		60	60	0	56	56	0					
Selvendran et al. 2022	10.4103/jcd.jcd_551_21	rct	Indirect pulp capping	Reversible pulpitis	Ca(OH)2 (Dycal, Dentsply, USA)	6 months	12	7	5							None	There are no conflicts of interest
					MTA (Angelus, Brazil)		12	10	2								
					Biodentine (Septodont, France)		12	11	1								
Asgary et al. 2022	https://doi.org/10.1007/s00784-021-04310-y	rct	Full pulpotomy	Revesible and irreversible	MTA (ProRoot,Dentsply, OK, USA)	24 months							51	51	0	The project was approved and supported by the Deputy Minister of Research, Iranian Ministry of Health and Medical Education	Dr. Asgary is the inventor of CEM cement (Endodontic Filling Material; USA, 7,942,961, 2011 May 17). All other authors declare that there are no conflict of interest regarding the publication of this paper
					CEM (BioniqueDent,Tehran, Iran)								47	46	1		
Doranala et al. 2021	10.4103/jcd.jcd_264_21	rct	Full pulpotomy	Irreversible pulpitis	TotalFill (EndoSequence root repair material)	6, 12 months	18	15	3	18	13	5				None	There are no conflicts of interest
					Ca(OH)2 (Dycal)		16	14	2	16	12	4					
Kaul et al. 2021	10.5005/jp-journals-10024–3084	rct	Indirect pulp capping	Reversible pulpitis	Biodentine (Septodont, Mumbai, India)	6, 12 months**	24	24	0	24	23	1				None	None
					Ca(OH)2 (Dycal)		24	23	1	24	22	2					
Rao et al. 2022	10.47750/pnr.2022.13.S07.310	rct	Direct pulp capping	Reversible pulpitis	MTA (NuSmileNeoMTA)	6, 12 months	15	14	1	15	14	1				N/A / Not mentioned	N/A / Not mentioned
					Ca(OH)2 (Dycal)		15	13	2	15	13	2					
Parameswaran et al. 2023	10.14744/eej.2023.83007	rct	Direct pulp capping	Reversible pulpitis	CEM (Bionique Dent Tehran, Iran)	6, 12 months	45	44	1	32	28	4				This study did not receive any financial support	The authors deny any conflict of interest
					MTA (Proroot, Dentsply)		44	40	4	32	25	7					
					Ca(OH)2 (Dycal, Dentsply)		38	27	11	31	17	14					
Singh et al. 2023	10.1007/s00784-023–05,136-6	rct	Partial pulpotomy	Reversible pulpitis	Ca(OH)2 (Dycal, Dentsply)	12 months				23	21	2				None	The authors declare no competing interests
					MTA (White ProRoot; Dentsply)					24	22	2					
					Biodentine (Septodont,France)					22	22	0					
Singla et al	10.4103/jcd.jcd_170_23	rct	Full pulpotomy	irreversible pulpitis	MTA (MTA Angelus, Brazil)	6, 12 months	22	16	6	22	15	7				JCD Dental College	There are no conflicts of interest
					Biodentine (Septodont, France)		23	17	6	23	16	7					
Tzanetakis et al. 2023	10.1111/iej.13955	rct	Partial pulpotomy	irreversible pulpitis	TotalFill (FKG)	6, 12 and 24 months	63	56	7	63	55	8	63	52	11	N/A / Not mentioned	The authors deny any conflict of interest related to the present study
					MTA (MTA Angelus)		74	66	8	74	66	8	74	66	8		

rct: randomized controlled trial.

* In this study the 36 months data were used.

** In this study 8 months and 16 monts data were used.

### Risk of Bias assessment

Five studies showed high risk, twelve had some concern, and four indicated low risk (Supplementary Fig. 1).

### Assessment of publication *bias*

All funnel plots were inverted and symmetric in all follow-up periods and subgroups, indicating no small study effect (Supplementary Figs. 2–11). Egger’s p values also indicate the lack of publication bias in all three follow-ups^72,73^.

### Certainty of evidence

After comparing materials based on pooled data at each follow-up interval, concerns regarding imprecision and heterogeneity prompted a downgrade in the certainty of evidence. The heterogeneity in pooled data at six and 24 month data was more pronounced. It was pronounced at those material comparisons where the number of direct data was small. Similarly, imprecision, heterogeneity, and incoherence warranted downgrading within the direct pulp capping subgroups. The appearance of heterogeneity in the direct pulp capping subgroup at all follow-up periods was pronounced. It was also pronounced in those material comparisons where the number of direct data was few. The main concerns in imprecision and heterogeneity were observed in the indirect pulp capping subgroup. The main concern in the partial and full pulpotomy subgroups was imprecision (see Supplementary Figs. 12–37). Consequently, the certainty of evidence ranged from high to very low across all subgroups.

### The outcome of the studies

#### Pooled data, including all treatment modalities at the six-month follow-up

The total number of patients included in the network analysis was 1932, with 257 failure events. The comparison structure is depicted in the network graph (Fig. 2A). Based on the p-score (Fig. 2B), the materials ranked from lowest to the highest OR as follows: CEM, MTA, Biodentine, TotalFill, TheraCalLC, Ca(OH)_2_, Ca(OH)_2_LC.

No significant difference was observed in the OR between CEM, Biodentine, Totalfill, and TheraCalLC groups compared to MTA (Fig. 2C, Supplementary Figs. 38, 48). However, the OR was significantly higher in the Ca(OH)_2_ and Ca(OH)_2_LC groups than in the MTA group.

#### Pooled data, including all treatment modalities at the 12 month follow-up

The total number of patients in the network analysis was 2158, with 333 failure events. The comparison structure is depicted in the network graph (Fig. 3A). Based on the p-score, the materials ranked from lowest to the highest OR as follows: MTA, CEM, Biodentine, TotalFill, TheraCalLC, Ca(OH)_2_, and Ca(OH)_2_LC (Fig. 3B).

No significant difference was observed in the OR between CEM, Biodentine, and TotalFill groups compared to MTA (Fig. 3C, Supplementary Figs. 39, 49). However, the OR was significantly higher in the TheraCalLC, Ca(OH)_2,_ and Ca(OH)_2_LC groups than in the MTA group.

#### Pooled data, including all treatment modalities at the 24 month follow-up

The total number of patients in the network analysis was 1107, with 257 failure events. The comparison structure is illustrated in the network graph (Fig. 4A). Based on the p-score, the materials ranked from lowest to the highest OR as follows: MTA, Biodentine, TheraCalLC, TotalFill, CEM, Ca(OH)_2_, and Ca(OH)_2_LC (Fig. 4B). The probability of MTA's rank gradually increased from six to 24 months.

No significant difference was observed in the OR between Biodentine, TotalFill, and CEM groups compared to MTA (Fig. 4C, Supplementary Figs. 40, 50). However, there was no direct comparison between CEM and the other materials except between CEM and MTA. The OR was significantly higher in TheraCalLC, Ca(OH)_2_, and Ca(OH)_2_LC groups than in the MTA group.

#### Indirect pulp capping at the six-month follow-up

Four studies were included with 239 patients and 17 failure events. The comparison structure is illustrated in the network graph (Fig. 5A). Based on the p-score (Fig. 5B), the materials ranked from lowest to highest OR are Biodentine, MTA, Ca(OH)_2_. No significant difference was observed in the OR between Biodentine and Ca(OH)_2_ groups compared to MTA (Fig. 5C, Supplementary Figs. 41, 51).

#### Direct pulp capping at the six-month follow-up

Seven studies were included with 1243 patients and 182 failure events. The comparison structure is depicted in the network graph (Fig. 6A). Based on the p-score (Fig. 6B), the materials ranked from lowest to highest OR are as follows: CEM, MTA, Biodentine, TheraCalLC, Ca(OH)_2_, Ca(OH)_2_LC.

No significant difference was observed in the OR between CEM, Biodentine, and TheraCalLC groups compared to MTA (Fig. 6C, Supplementary Figs. 42, 52). Failure was significantly more likely in Ca(OH)_2_ and Ca(OH)_2_LC groups higher than in the MTA group. However, there were just two direct comparisons of CEM with Ca(OH)_2_ and MTA.

#### Direct pulp capping at the 12 month follow-up

Six studies were included, with 1122 and 231 failure events. The comparison structure is illustrated in the network graph (Fig. 7A). Based on the p-score, the materials ranked from lowest to the highest OR as follows: CEM, MTA, Biodentine, TheraCalLC, Ca(OH)_2_, and Ca(OH)_2_LC (Fig. 7B).

No significant difference in the OR was observed between CEM, Biodentine, and TheraCalLC groups compared to MTA (Fig. 7C, Supplementary Figs. 43, 53). The OR was significantly higher in TheraCalLC, Ca(OH)_2,_ and Ca(OH)_2_LC groups than in the MTA group.

#### Direct pulp capping at the 24 month follow-up

Three studies were included with 667 patients and 209 failure events. The comparison structure is shown in the network graph (Fig. 8A). Based on the p-score, the materials ranked from lowest to the highest OR as follows: MTA, Biodentine, TheraCalLC, Ca(OH)_2_, and Ca(OH)_2_LC (Fig. 8B).

No significant difference was observed in the OR between Biodentine compared to MTA (Fig. 8C, Supplementary Figs. 44, 54). The OR was significantly higher in TheraCalLC, Ca(OH)_2,_ and Ca(OH)_2_LC groups than in the MTA group.

#### Partial pulpotomy at the 12 month follow-up

Three studies were included with 250 patients and 33 failure events. The comparison structure is shown in the network graph (Fig. 9A). Based on the p-score (Fig. 9B), the materials ranked from lowest to the highest OR are as follows: Biodentine, MTA, TotalFill, Ca(OH)_2_.

No significant difference was observed in the OR between Biodentine, TotalFill, and Ca(OH)_2_ groups compared to MTA (Fig. 9C, Supplementary Figs. 45, 55).

#### Full pulpotomy at the six-month follow-up

Three studies were included with 207 patients and 27 failure events. The comparison structure is shown in the network graph (Fig. 10A). Based on the p-score (Fig. 10B), the materials ranked from lowest to highest OR are as follows: Ca(OH)_2_, TotalFill, MTA, and Biodentine.

No significant difference was observed in the OR between Ca(OH)_2_, TotalFill, and Biodentine groups compared to MTA (Fig. 10C, Supplementary Figs. 46, 56).

#### Full pulpotomy at the 12 month follow-up

Four studies were included with 581 patients and 56 failure events. The comparison structure is depicted in the network graph (Fig. 11A). Based on the p-score (Fig. 11B), the materials ranked from lowest to highest OR are as follows: Biodentine, Ca(OH)_2_, MTA, TotalFill, and CEM. Compared to MTA, the OR was not significantly different between the Biodentine, Ca(OH)2, TotalFill, and CEM groups (Fig. 11C, Supplementary Figs. 47, 57).

## Discussion

The current network meta-analysis showed solid statistical evidence for MTA over Ca(OH)_2_ products, such as Ca(OH)_2_ and Ca(OH)_2_LC, with a 2–3 times higher failure rate in VPT over two years. Therefore, the first null hypothesis comparing Ca(OH)_2_ products, such as Ca(OH)_2_ and Ca(OH)_2_LC with MTA, regardless of the treatment modalities, was rejected.

The second hypothesis was partially rejected. The Ca(OH)_2_ resulted in three times more failure than the MTA in direct pulp capping. No significant difference was observed between MTA and Ca(OH)_2_ in pulpotomy groups. Nevertheless, only a few studies were eligible and did not compare directly (full pulpotomy at 6 and 12 months), resulting in high confidence intervals. However, it is not rational based on its significant inferiority even in the least severe pulp lesion, including Ca(OH)_2_ in future pulpotomy randomized clinical trials.

In contrast to our results, most previous studies^34,49,62,64^ found no significant difference between Ca(OH)_2_ and MTA at six-month follow-ups. Despite the higher odds ratio for Ca(OH)_2_ and for Ca(OH)_2_LC than for MTA, the limited sample size of the individual studies prevented a conclusion from being drawn. However, synthesizing the individual study results by meta-analysis could provide a high level of evidence^74,75^.

In addition, a recent retrospective cohort study^76^ has found success rates of 100%, 95%, 95%, 86%, and 89% for direct pulp capping at 1, 5, 10, 20, and 35 years of follow-up with Ca(OH)_2_ (Dycal, Dentsply), respectively. However, the careful methodology developed by the author might explain these excellent results. In the case of medium/large pulp exposure, the calcium-hydroxide powder was applied to the pulp to control the moisture before Dycal application. It is important to note that the same person performed the treatment and evaluation, indicating a possible bias.

Most randomized clinical trials that compared Ca(OH)_2_ to MTA^49,50,61–64,67,68,71^ included teeth with reversible pulpitis and direct or indirect pulp capping without pulpotomy. The success rates of these studies varied widely, between 58 and 96% at six months, 64 and 92% at 12 months, and 40 and 92% at 24 months. However, the success rates of MTA in the same studies (direct comparison) were more consistent, between 84 and 100% at six months and 83 and 98% at 12 months. At 24 months, the success rates ranged from 85 to 100% after excluding one study^61^. This study reported a crude failure rate of 13.6%. However, to align with the methodology of other studies in the meta-analysis, the failure rate was recalculated, excluding lost cases. This recalculation yielded an unrealistically low failure rate of 45% with MTA. The involvement of 43 dental practices may have introduced bias, leading to the loss of an exceptionally high number of cases (135 out of 183) during follow-up, thus significantly distorting the reported rates.

No subgroup analysis could be conducted in this meta-analysis for pulpotomy because only one randomized clinical trials^34^ performed pulpotomy with Ca(OH)_2_. In that particular study, the odds ratios for Ca(OH)_2_/MTA were high: 3.23 at six months, 4.09 at 12 months, and 7.15 at 24 months, indicating a clear advantage of MTA over the Ca(OH)_2_ in pulpotomy after irreversible pulpitis.

In a histological study on animals, Ca(OH)_2_ induced less dentin bridge formation after direct pulpal application than MTA^77^. Twelve of the 15 teeth that underwent Ca(OH)_2_ treatment developed pulp inflammation; only the rest formed dentin bridges without signs of inflammation. It is hypothesized that the inadequate adhesion of calcium-hydroxide-based materials to the tooth structure, their low mechanical strength, and solubility can result in microleakage and dental pulp infection^78^. Ca(OH)_2_ forms only a weak mineralized barrier with high permeability. The high dissolvability of Ca(OH)_2_ disintegrates the material, leaving voids ^79^. This is a potential way for reinfection and a possible reason for the higher failure rate in VPT^79^.

Compact hard tissue formation without bacterial invasion is crucial for successful vital pulp treatment, and this depends on the sealing ability of the capping material^80^. In contrast to Ca(OH)_2_, bioactive tricalcium silicate-based cements can create an ideal healing environment and form reparative dentin^81^. They also have a better sealing ability than CaOH_2_, which is likely one of the main reasons for their success^79,80^.

The hypothesis that the newer bioactive materials would be as efficient in VPT as MTA was partially rejected as TheraCalLC was inferior to MTA at 12 and 24 months by significant odds ratios of 2.09 and 2.10. TheraCalLC is a resin-modified light-curing calcium silicate-based material. The TheraCalLC was only used for direct pulp capping in the included studies. The material has low solubility, fast setting time, and good sealing ability^82^. However, no calcium-hydroxide formation was observed during hydration with TheraCalLC^83^. Calcium-hydroxide is highly effective in stimulating odontoblast activity, directly resulting in mineralization^84^. Therefore, these specific properties might be attributed to the low performance at 12 and 24 months.

CEM has an antimicrobial effect^37^ and a good sealing ability^36^, which are essential for the success of VPT. This meta-analysis comparing CEM to MTA, including all treatment modalities, indicated a non-significant odds ratio of 0.15, 0.97, and 3.32 at six, 12, and 24 months. Similar results were obtained in the direct pulp capping subgroup with reversible pulpitis at both six and 12 month follow-ups in the full pulpotomy subgroup with irreversible pulpitis at 12 months. However, only three randomized clinical trials^4,60,67^ could be included for comparison between CEM and MTA. Consequently, more studies are necessary to increase the level of evidence for CEM.

The network meta-analysis showed that Totalfill was not inferior to MTA at each follow-up in the pooled subgroups. Three studies were included in the six- and 12-month follow-ups, but only one study in the 24 month follow-ups. The TotalFill demonstrated non-inferiority to MTA in the 12 month partial pulpotomy and six- and 12 month full pulpotomy subgroups. It should be noted that the partial pulpotomy subgroup included one study with TotalFill, while the full pulpotomy subgroups included three studies. Totalfill is a calcium silicate-based repair material with beneficial biological properties^58^. The manufacturer already premixes TotalFill, so no further preparation is required. TotalFill demonstrates optimal biocompatibility, no cytotoxicity, and has an osteogenic property^85^. Totalfill does not shrink during setting and is hydrophilic and insoluble in tissue fluids^86^.

The current meta-analysis showed that the clinical efficacy of Biodentine in VPT is not inferior to MTA in any subgroups of treatment modality, suggesting that it has an excellent alternative. Biodentine shows high cytocompatibility^87^ and has good antimicrobial potential^88^. Fast setting time and less discoloring potential are favorable clinical properties^30^. Biodentine has beneficial regenerative properties that improve dental pulp stem cell proliferation, attachment, and migration^89^. Furthermore, Biodentine was more effective in promoting mineralization than MTA^90^.

A recently published meta-analysis compared calcium silicate and hydroxide-based materials to MTA^91^. However, this study performed a network meta-analysis to compare all materials studied directly and indirectly. Through the network meta-analysis, additional materials could be included, such as TheraCalLC, TotalFill, and Ca(OH)_2_LC. Consequently, materials could be ranked according to their clinical efficacy. Another distinction was that we excluded immature and trauma cases and focused solely on mature permanent teeth, which is more clinically relevant for dentists treating adult patients. In addition, we included 24-month follow-up periods contrary to the 12 month follow-up in the previous study. Although the authors of the previous meta-analysis noted that additional studies were required to determine success rates accurately, we addressed this issue using a bias elimination calculation method.

### Strengths and limitations

An essential strength of the current analysis is that all studies included were randomized controlled trials with large sample sizes. In addition, some original data was reevaluated to derive precise estimates of odds ratios by standardizing the calculation across the studies. A successful subgroup analysis for the direct pulp capping revealed that Ca(OH)_2_ performs poorly even in the least severe pulp lesions.

However, the present study has limitations due to weak arms for some of the comparisons and subgroups. Specifically, CEM was compared only to MTA and Ca(OH)_2_ at six and 12 months and only to MTA at 24 months, whereas Totalfill was only compared to MTA and Biodentine and Ca(OH)_2_. In addition, CEM was used in only three studies, warranting caution in interpreting the results. These weaker comparison arms may impact the accuracy of the findings. However, as the odds ratio of conventional Ca(OH)_2_ is significantly higher than MTA, it would be unfeasible and ethically questionable to include Ca(OH)_2_ in a large-scale study comparing new bioactive materials to it.

In the pooled results, heterogeneity was not a severe problem. However, in subgroup analyses, heterogeneity increased, but imprecision was the primary concern. This is because there are few randomized clinical trials with more clinically complicated situations, such as symptomatic cases with a partial or complete pulpotomy. Four studies^4,53,58,69^ included full pulpotomy, and three studies^34,68,70^ included partial pulpotomy. In one study^60^, the extent of the pulpotomy was not defined, and another study did not distinguish between direct pulp capping and full pulpotomy^51^. The rest of the studies did not apply pulpotomy or did not declare it.

The longest follow-up in the included studies was 24 months, except for one article, Peskersoy et al.^64^. The limited duration of follow-up for VPT restricts the ability to draw long-term conclusions from the results.

### Implications for research

A review of the articles clarified that critical demographic data, such as sex, age, and tooth types, were either not published or lacked stratified results. Given the influence of age in clinical decision-making, it is necessary to conduct studies involving mature permanent teeth, including middle-aged and possibly elderly individuals.

It is also crucial to report the number of cases lost to follow-up and calculate failure rates considering the lost cases.

In conventional asymptomatic pulp capping, MTA has shown promising results. However, neither study directly compared the reversible and irreversible pulpitis; many did not classify the pulpal state, nor did the studies use the Wolters classification^47^. Further studies with larger sample sizes are needed to establish the best clinical practice for treating irreversible pulpitis, as recommended by Wolters et al.^47^.

More extended follow-up periods are strongly recommended. Some of the included studies did not report all follow-up periods. It would also be helpful to look at the trends in failure over the whole follow-up period*.*

### Implications for practice

Ca(OH)_2_ is not recommended for any vital pulp treatment, including pulp capping. However, newer calcium silicate-based materials such as Biodentine and Totalfill, which offer improved treatment properties, can be considered as alternatives to MTA. Based on the results of the present study, it can be concluded that MTA has the best clinical performance in VPT. It is important to emphasize that the handling and setting time of MTA is worse than that of Biodentine. In those clinical situations where setting time is relevant, Biodentine is the preferred material. Newer MTA products may be better at handling tooth discoloration, but they are still inferior to Biodentine. In cases where aesthetics are mandatory (in the anterior region), Biodentine could be a better alternative for VPT. Failure can occur even after six months without symptoms, so long-term monitoring is essential.

## Conclusion

The network meta-analysis indicated a high to very low confidence rating that Mineral Trioxide Aggregate and Biodentine are superior materials for vital pulp treatment during 6, 12, and 24 months compared to calcium-hydroxide-based materials. Based on the 2–3 times higher failure rate, the clinical application of Ca(OH)_2_ and Ca(OH)_2_LC for VPT can no longer be recommended for direct pulp-capping for pulpotomy. With a moderate to low confidence rating, Biodentine and Totalfill could be a suitable MTA substitute.

### Supplementary Information

Supplementary Figure 1.

Supplementary Figure 2.

Supplementary Figure 3.

Supplementary Figure 4.

Supplementary Figure 5.

Supplementary Figure 6.

Supplementary Figure 7.

Supplementary Figure 8.

Supplementary Figure 9.

Supplementary Figure 10.

Supplementary Figure 11.

Supplementary Figure 12.

Supplementary Figure 13.

Supplementary Figure 14.

Supplementary Figure 15.

Supplementary Figure 16.

Supplementary Figure 17.

Supplementary Figure 18.

Supplementary Figure 19.

Supplementary Figure 20.

Supplementary Figure 21.

Supplementary Figure 22.

Supplementary Figure 23.

Supplementary Figure 24.

Supplementary Figure 25.

Supplementary Figure 26.

Supplementary Figure 27.

Supplementary Figure 28.

Supplementary Figure 29.

Supplementary Figure 30.

Supplementary Figure 31.

Supplementary Figure 32.

Supplementary Figure 33.

Supplementary Figure 34.

Supplementary Figure 35.

Supplementary Figure 36.

Supplementary Figure 37.

Supplementary Figure 38.

Supplementary Figure 39.

Supplementary Figure 40.

Supplementary Figure 41.

Supplementary Figure 42.

Supplementary Figure 43.

Supplementary Figure 44.

Supplementary Figure 45.

Supplementary Figure 46.

Supplementary Figure 47.

Supplementary Figure 48.

Supplementary Figure 49.

Supplementary Figure 50.

Supplementary Figure 51.

Supplementary Figure 52.

Supplementary Figure 53.

Supplementary Figure 54.

Supplementary Figure 55.

Supplementary Figure 56.

Supplementary Figure 57.

Supplementary Legends.

Supplementary Table 1.

Supplementary Table 2.

## Data Availability

The data that support the findings of this study are available upon reasonable request from the corresponding author. Relevant reporting guidelines paperwork (see 5.2 Reporting Guidelines below) This protocol for the systematic review was registered in the PROSPERO database (registration number CRD42022375573) and adheres to the Preferred Reporting Items for Systematic Reviews (PRISMA-NMA) guidelines.
