# Supplementary figures and images for: Comparison of bioactive material failure rates in vital pulp treatment of permanent matured teeth – a systematic review and network meta-analysis
Source: Sci Rep. 2024 Aug 8;14:18421. doi: 10.1038/s41598-024-69367-7 (PMC11310317; doi:10.1038/s41598-024-69367-7)

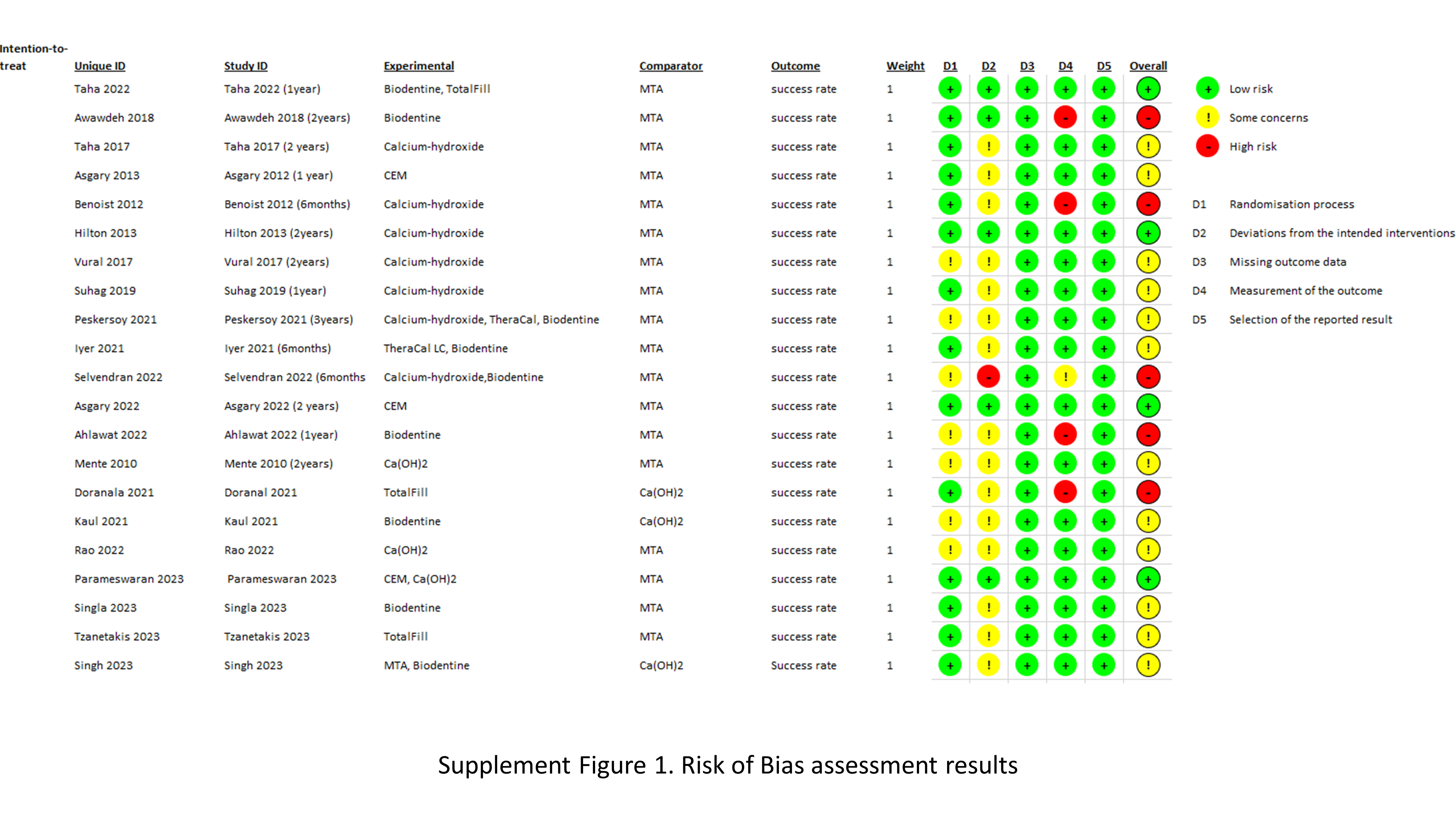

Supplement: Supplementary file 1 — Supplementary Figure 1. [file 41598_2024_69367_MOESM1_ESM.tif]

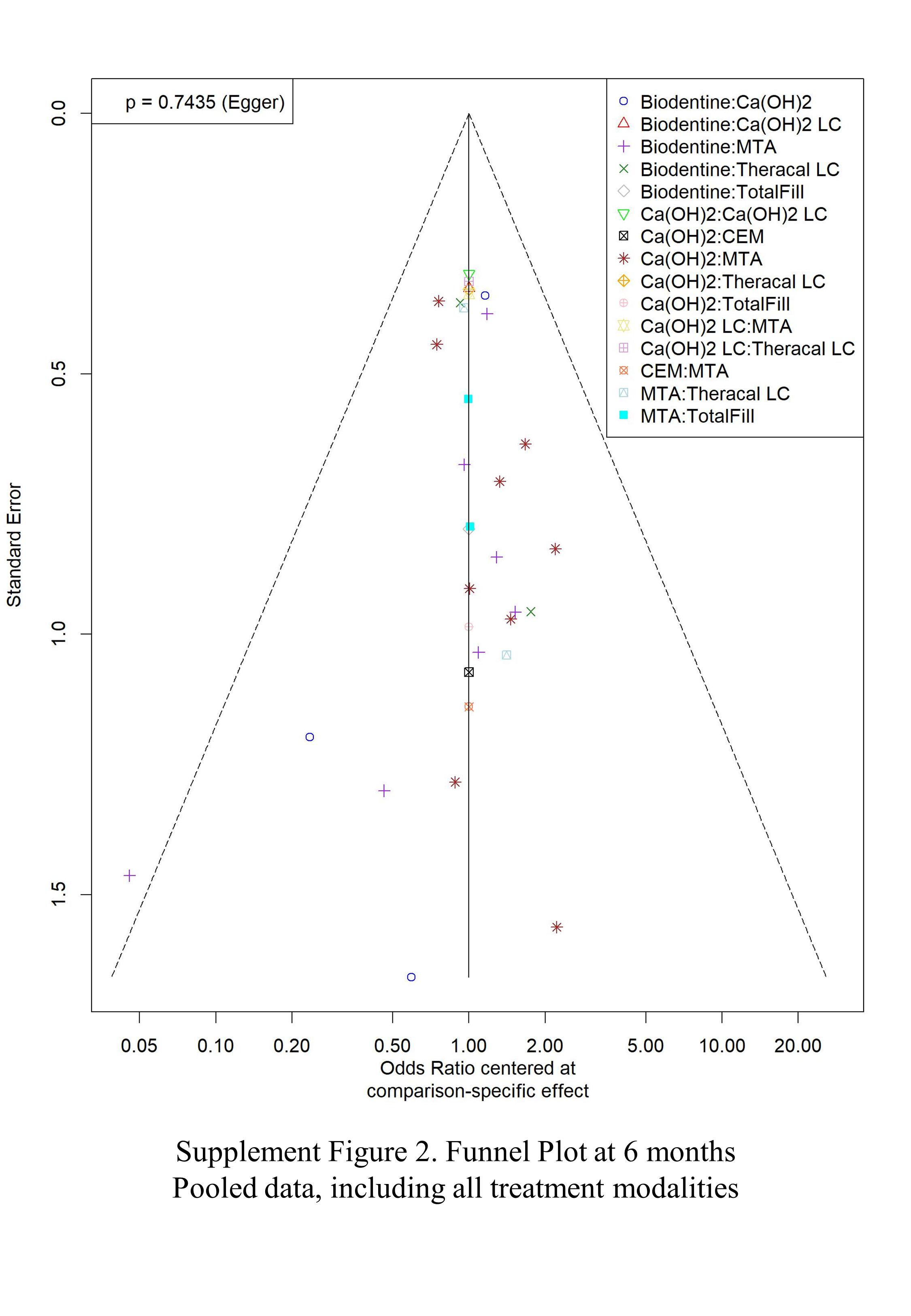

Supplement: Supplementary file 2 — Supplementary Figure 2. [file 41598_2024_69367_MOESM2_ESM.tif]

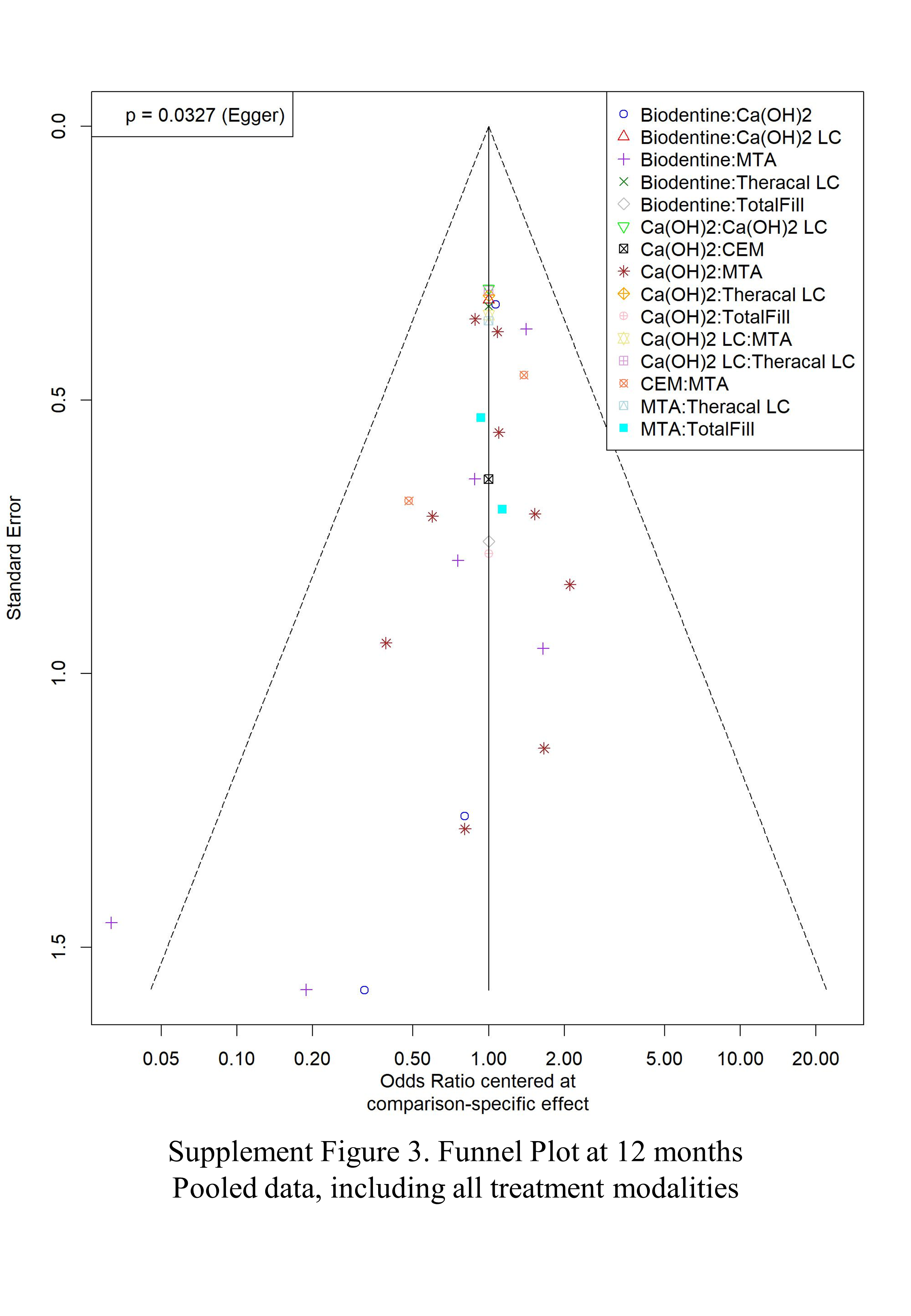

Supplement: Supplementary file 3 — Supplementary Figure 3. [file 41598_2024_69367_MOESM3_ESM.tif]

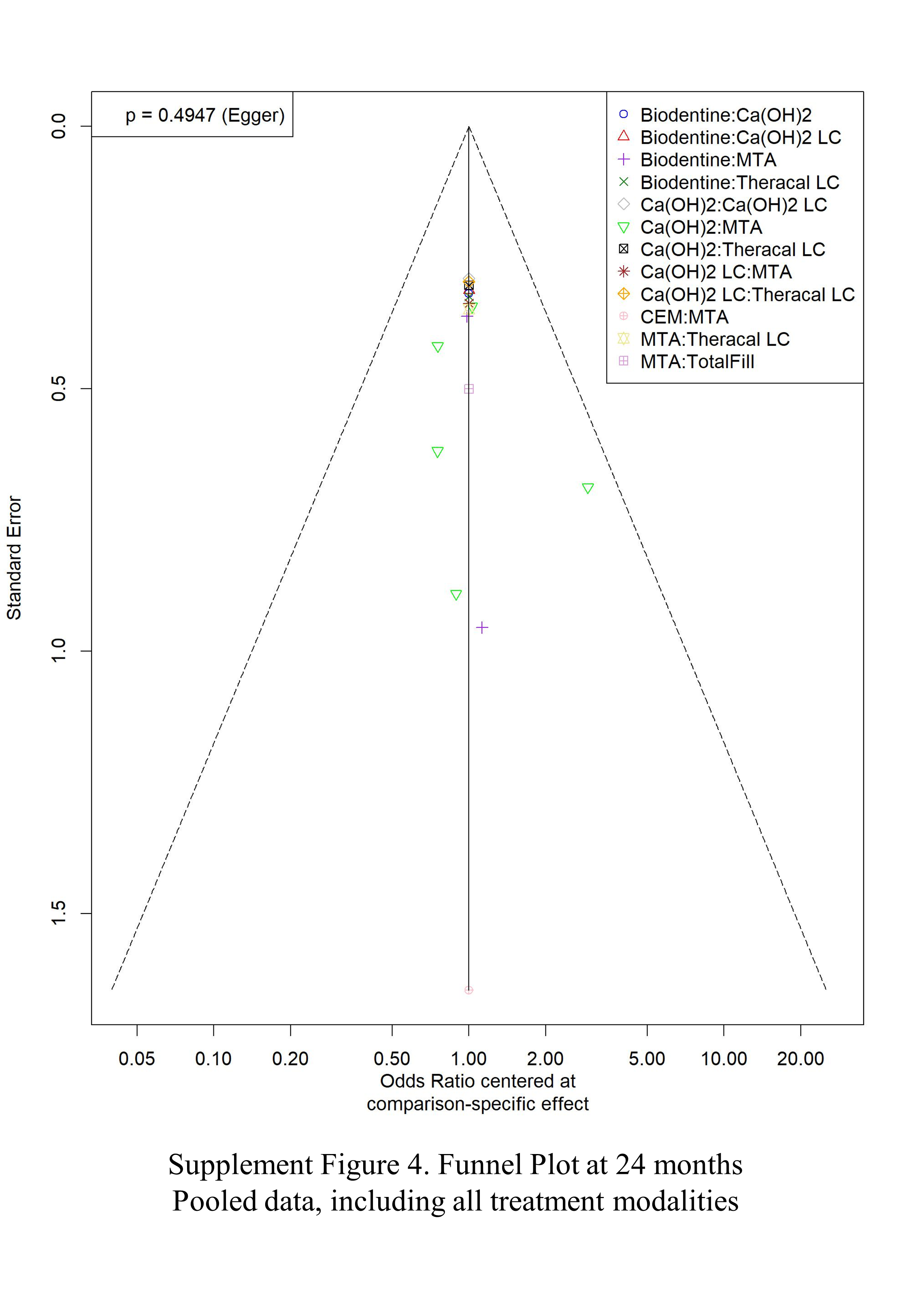

Supplement: Supplementary file 4 — Supplementary Figure 4. [file 41598_2024_69367_MOESM4_ESM.tif]

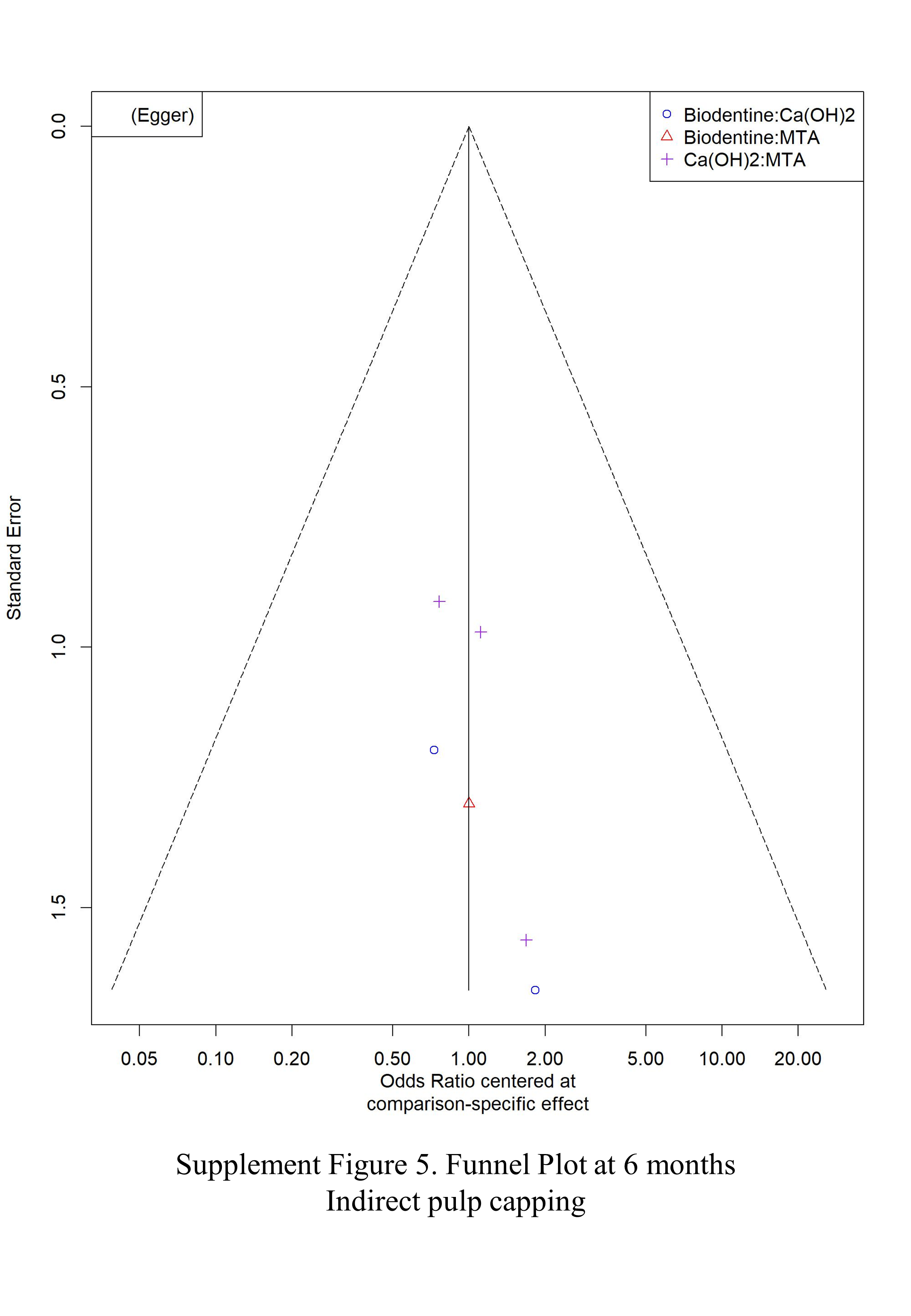

Supplement: Supplementary file 5 — Supplementary Figure 5. [file 41598_2024_69367_MOESM5_ESM.tif]

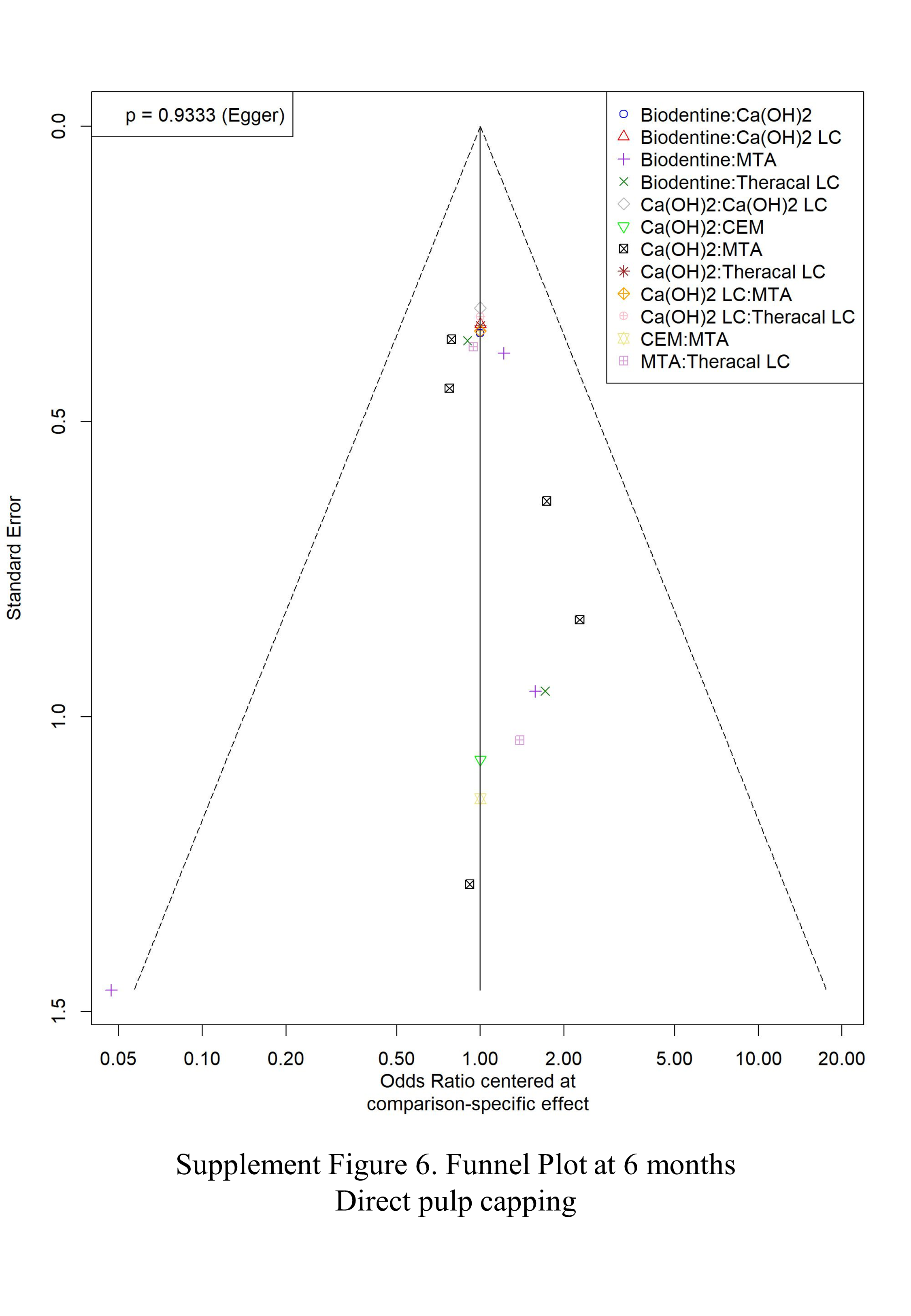

Supplement: Supplementary file 6 — Supplementary Figure 6. [file 41598_2024_69367_MOESM6_ESM.tif]

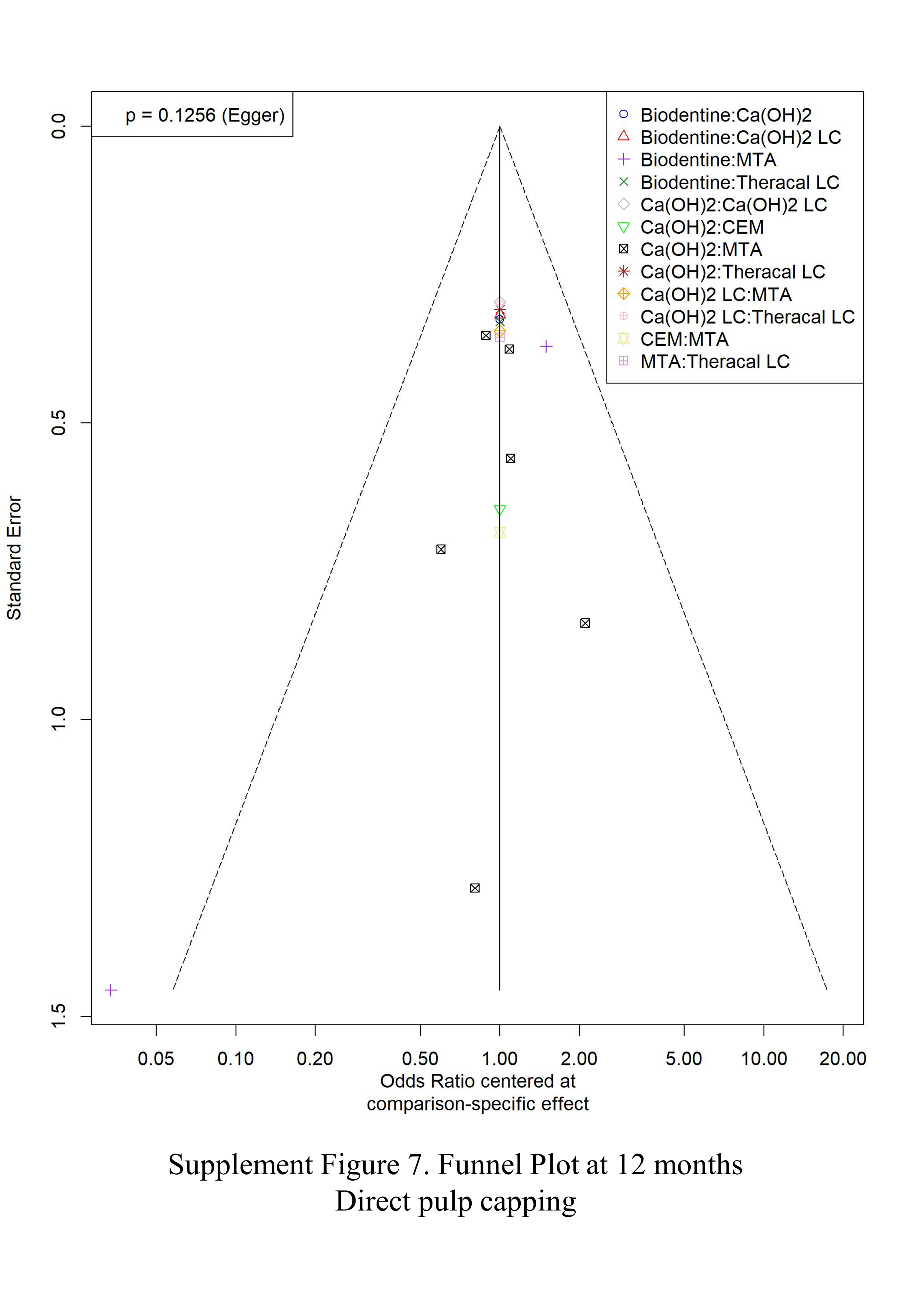

Supplement: Supplementary file 7 — Supplementary Figure 7. [file 41598_2024_69367_MOESM7_ESM.tif]

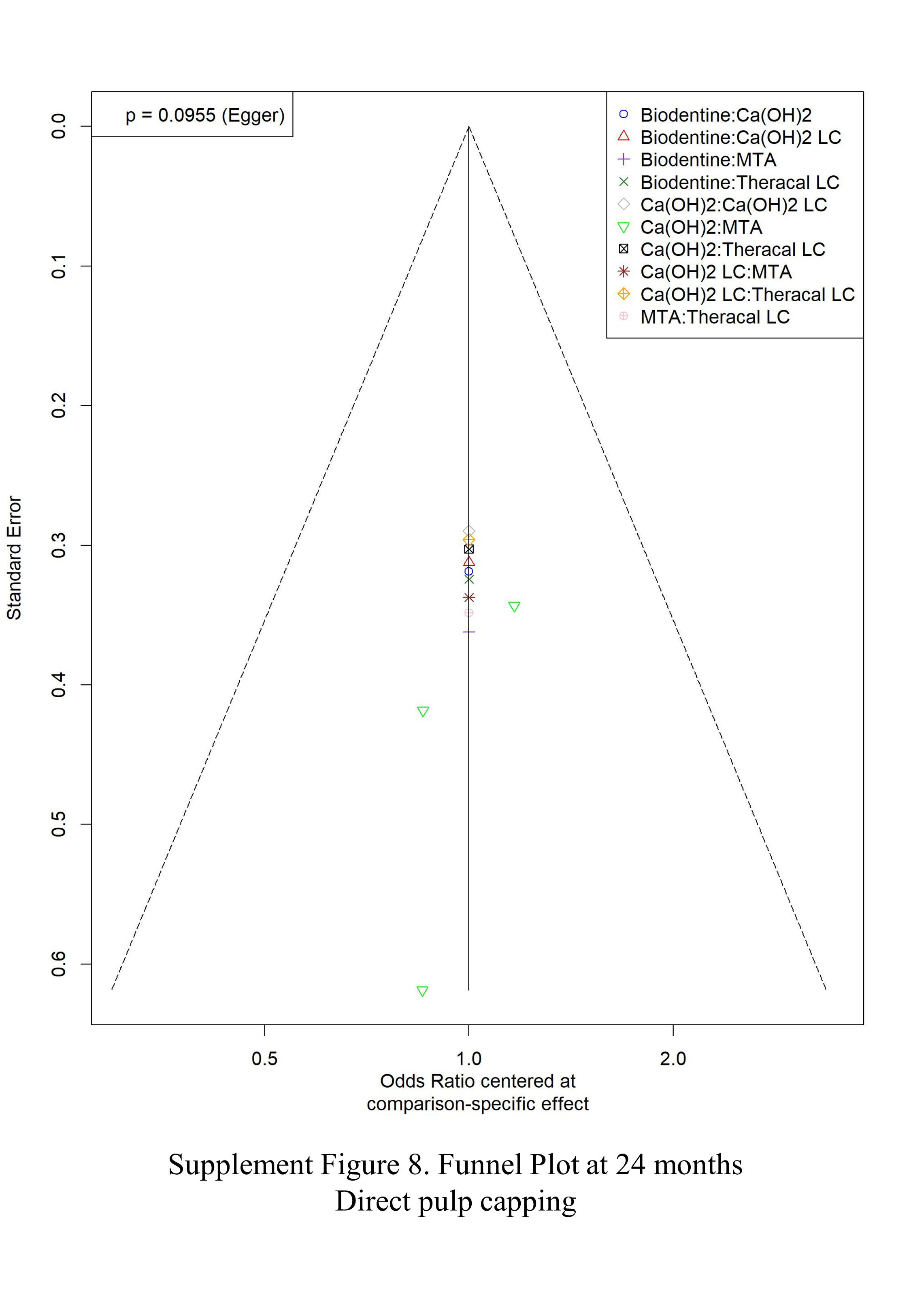

Supplement: Supplementary file 8 — Supplementary Figure 8. [file 41598_2024_69367_MOESM8_ESM.tif]

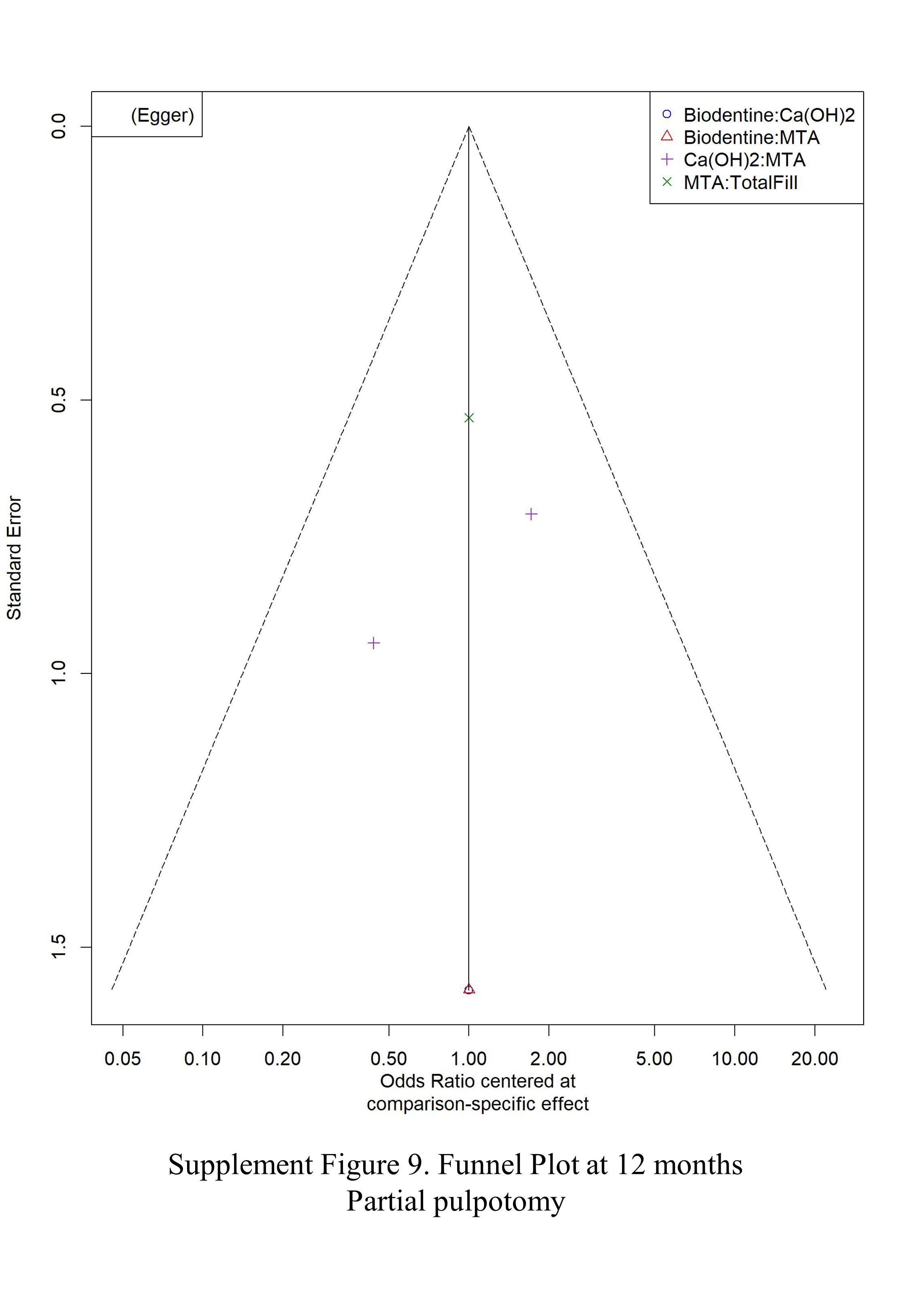

Supplement: Supplementary file 9 — Supplementary Figure 9. [file 41598_2024_69367_MOESM9_ESM.tif]

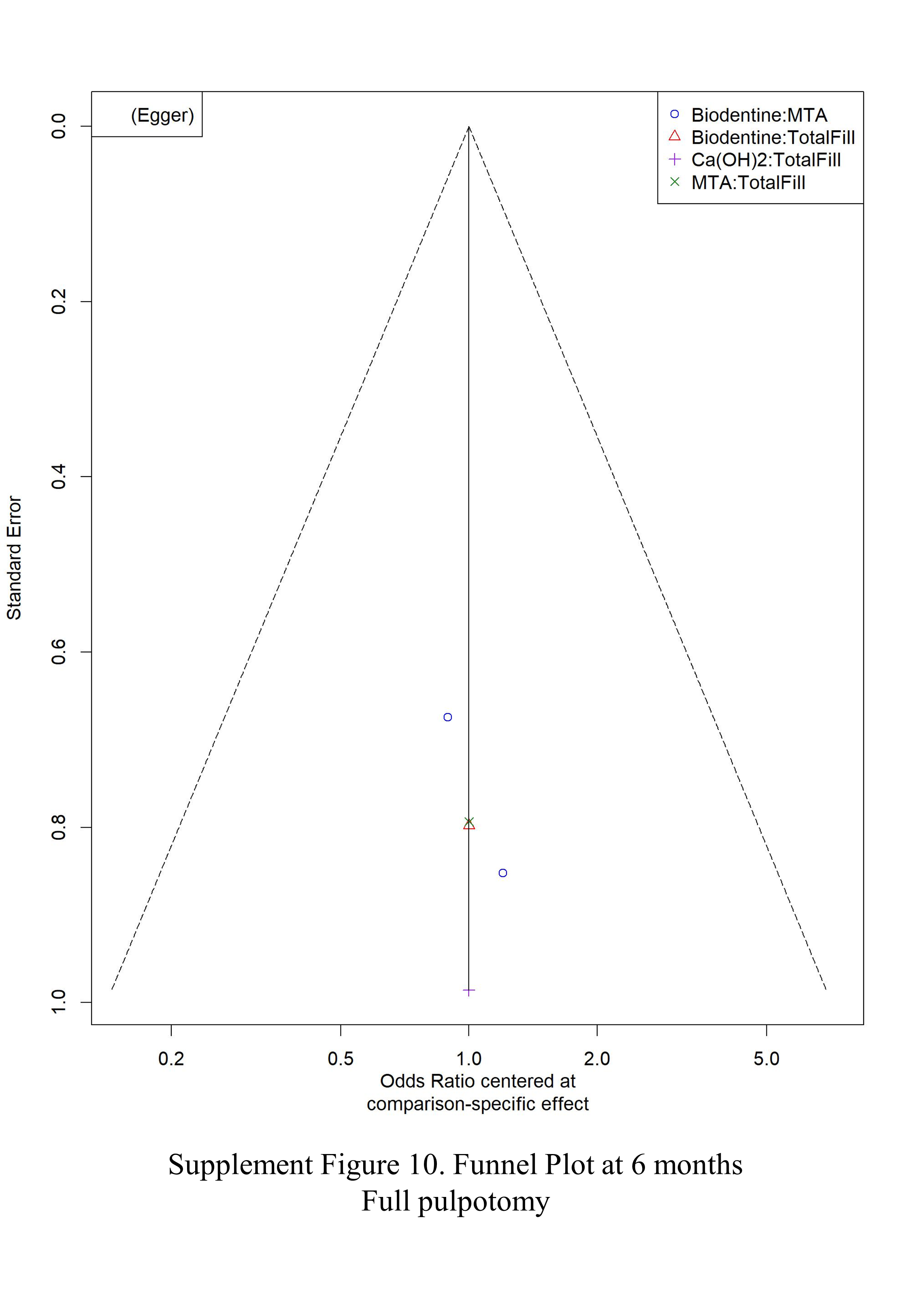

Supplement: Supplementary file 10 — Supplementary Figure 10. [file 41598_2024_69367_MOESM10_ESM.tif]

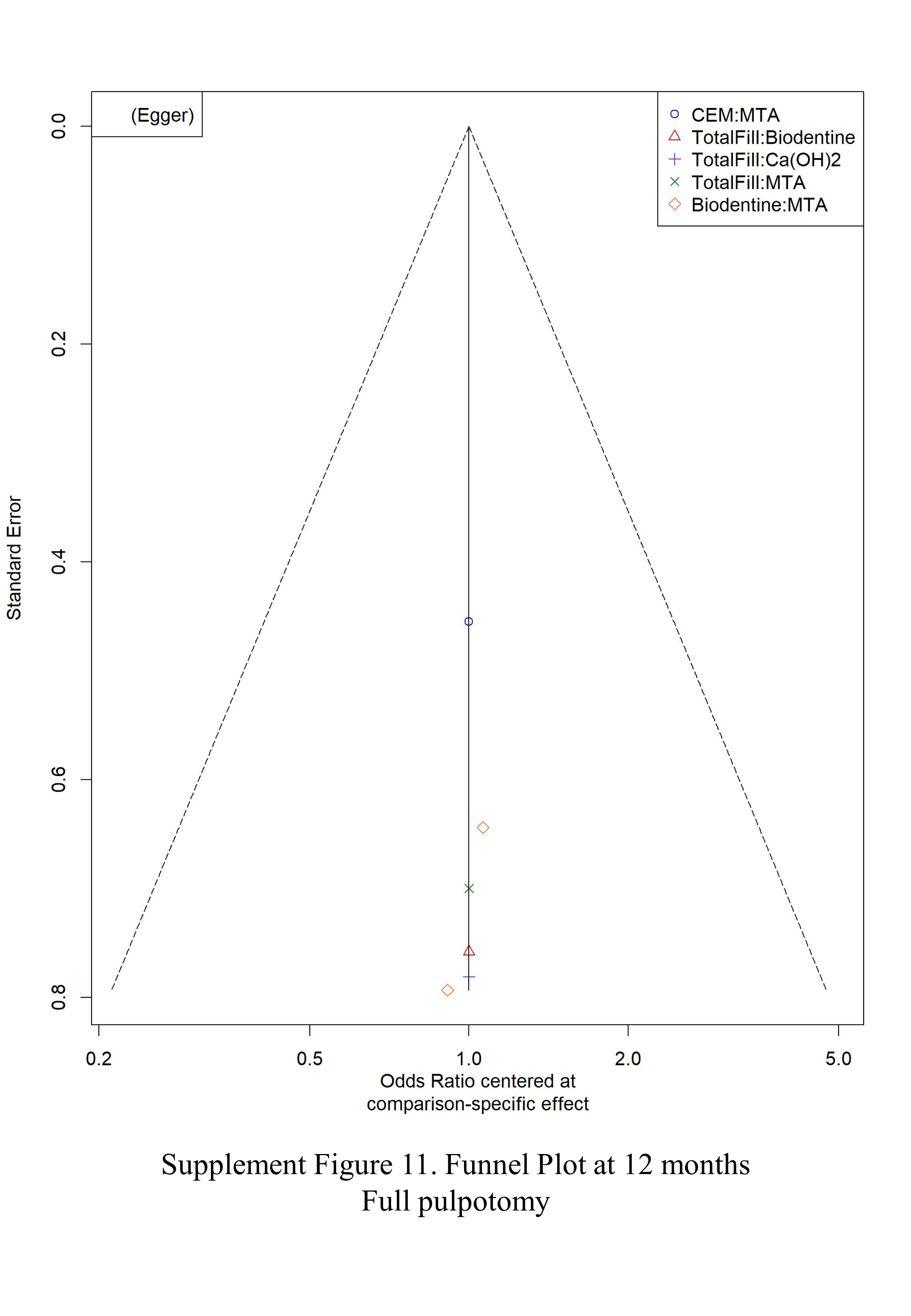

Supplement: Supplementary file 11 — Supplementary Figure 11. [file 41598_2024_69367_MOESM11_ESM.tif]

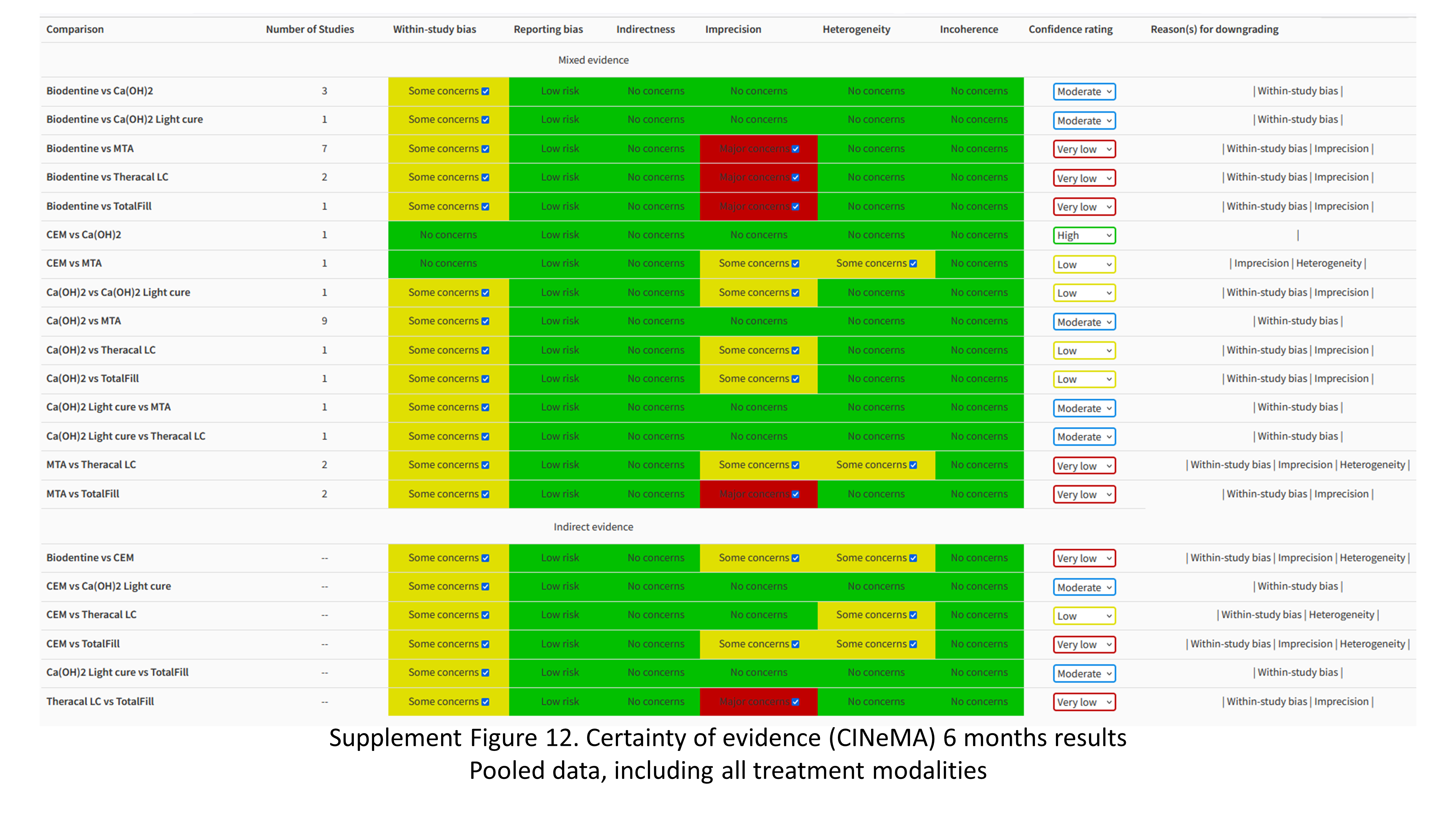

Supplement: Supplementary file 12 — Supplementary Figure 12. [file 41598_2024_69367_MOESM12_ESM.tif]

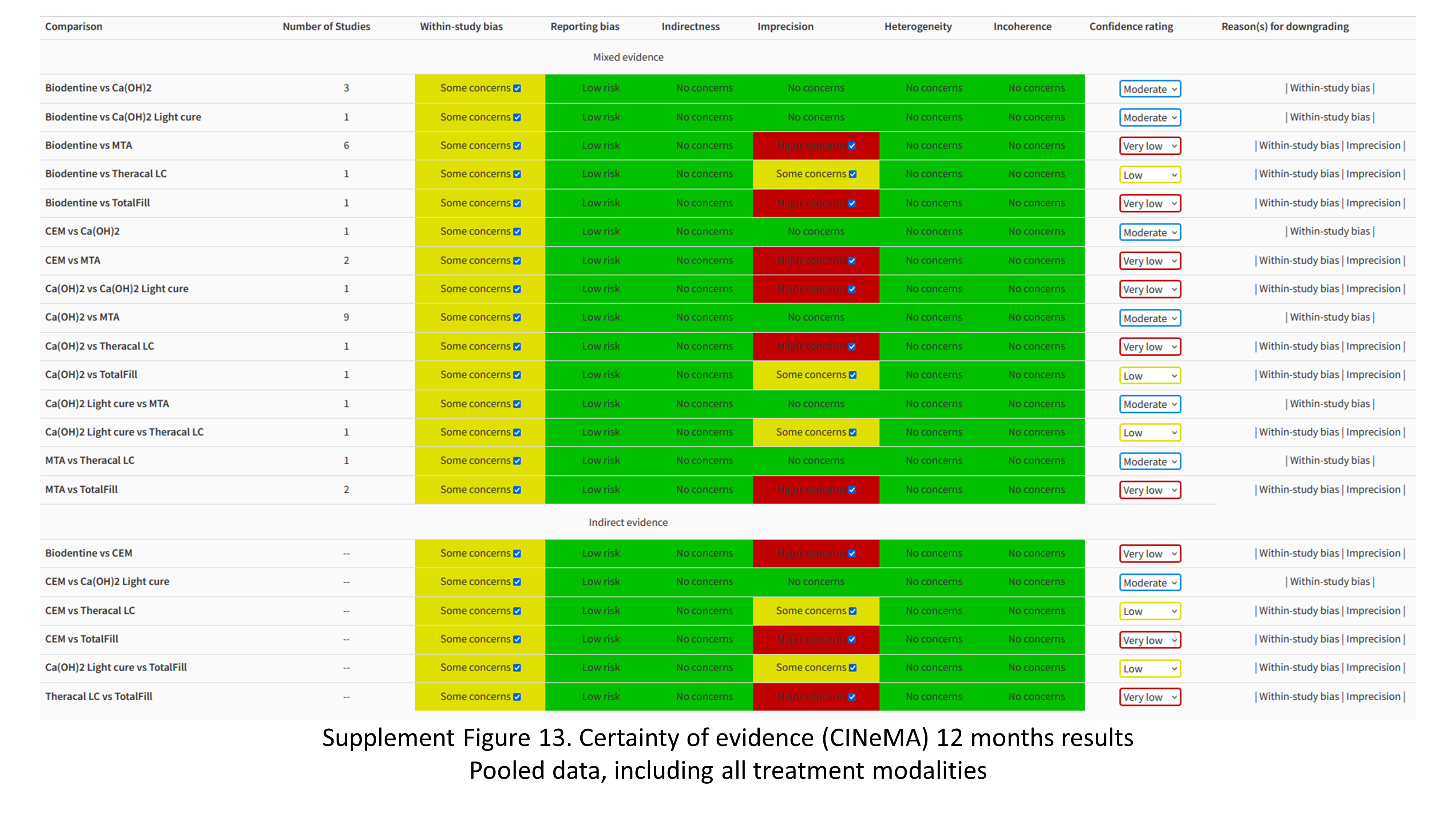

Supplement: Supplementary file 13 — Supplementary Figure 13. [file 41598_2024_69367_MOESM13_ESM.tif]

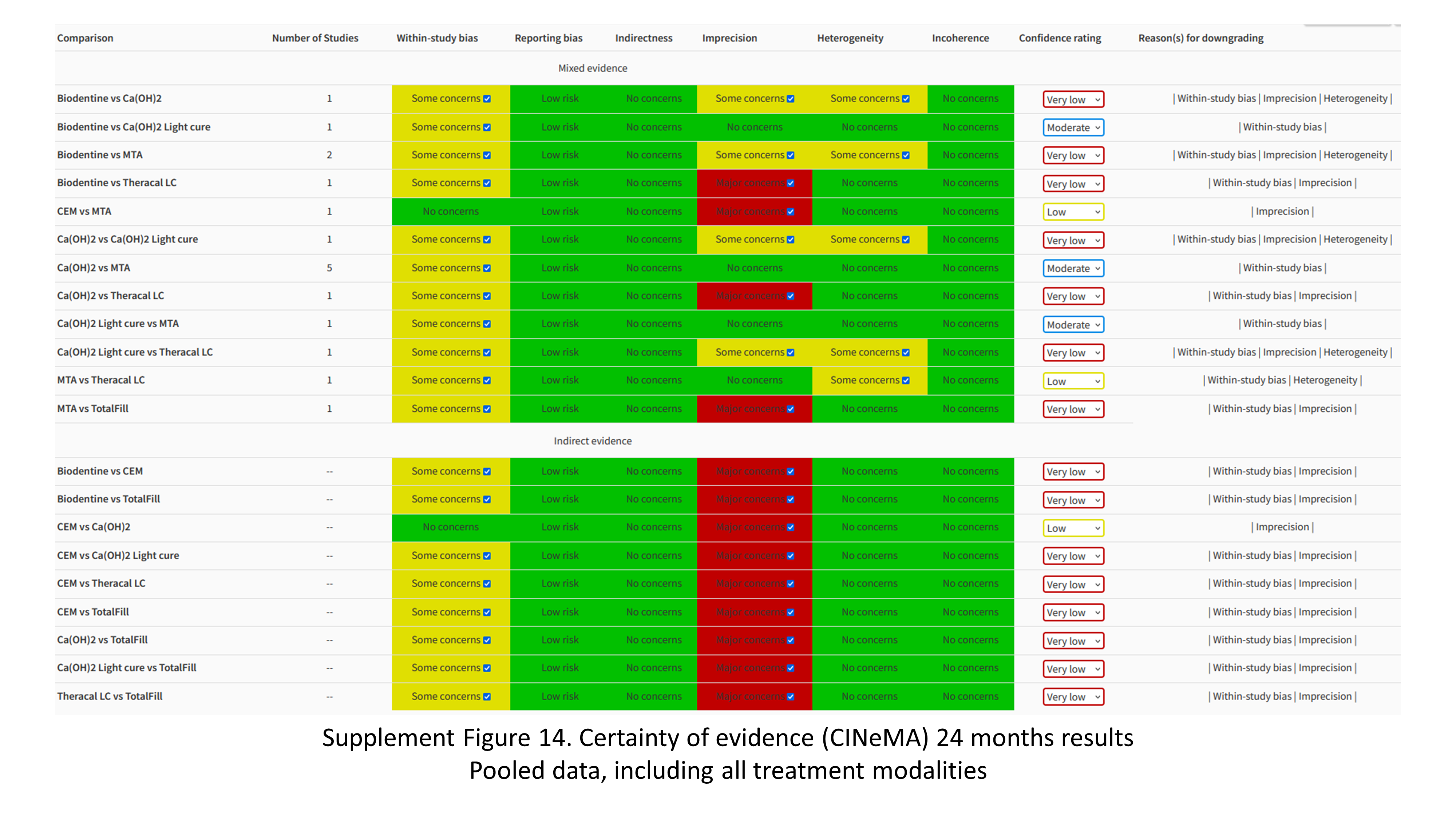

Supplement: Supplementary file 14 — Supplementary Figure 14. [file 41598_2024_69367_MOESM14_ESM.tif]

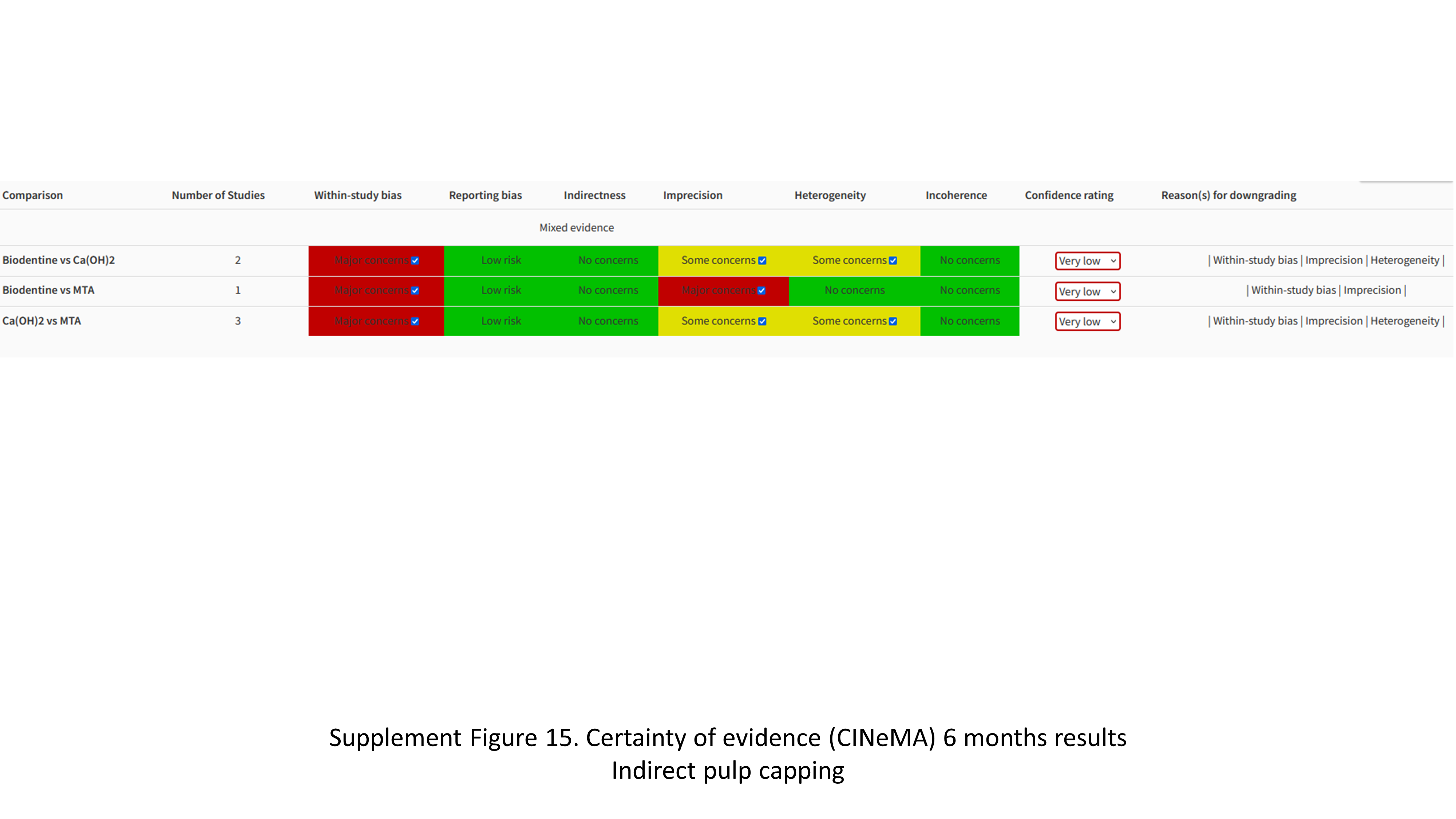

Supplement: Supplementary file 15 — Supplementary Figure 15. [file 41598_2024_69367_MOESM15_ESM.tif]

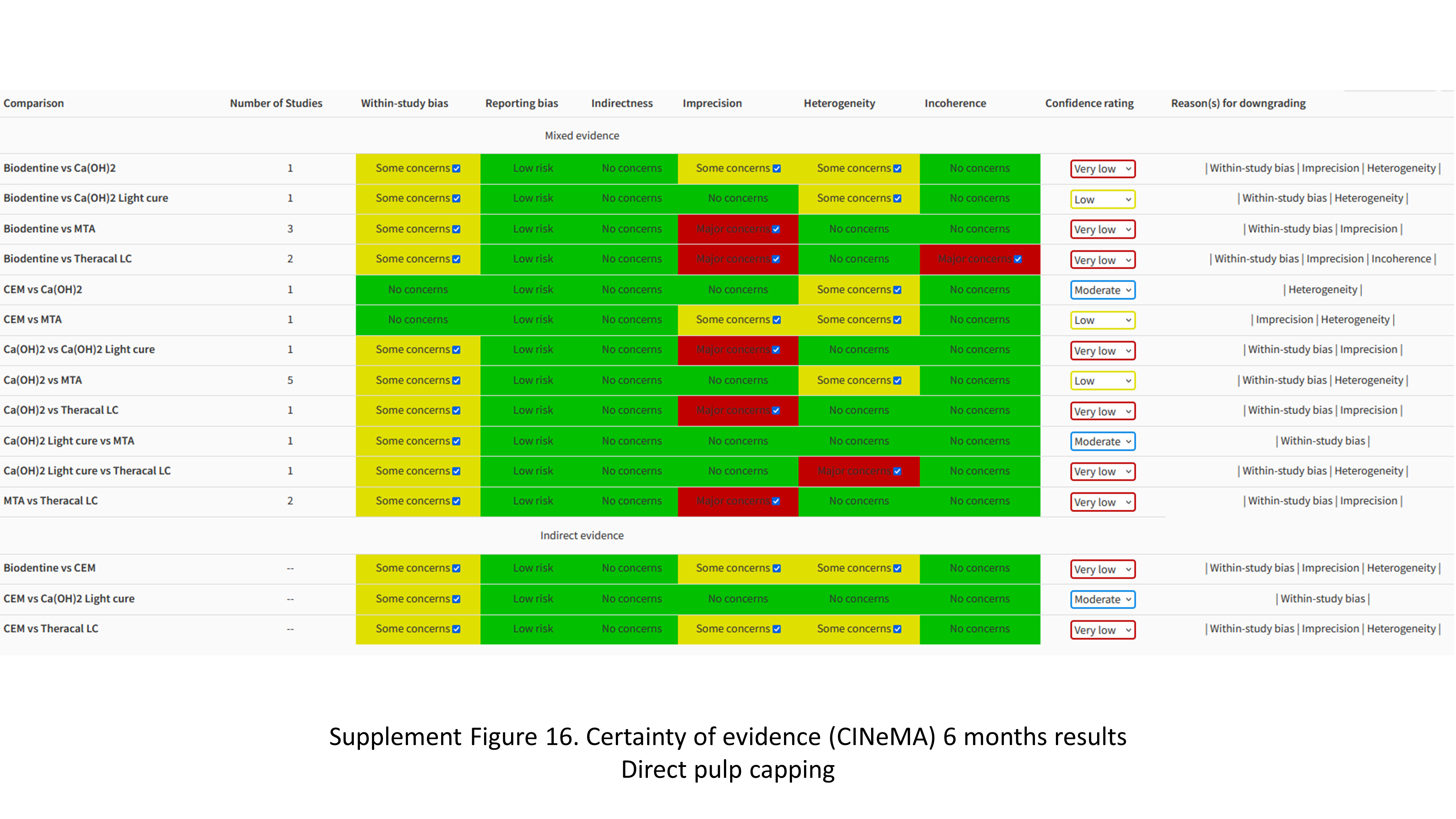

Supplement: Supplementary file 16 — Supplementary Figure 16. [file 41598_2024_69367_MOESM16_ESM.tif]

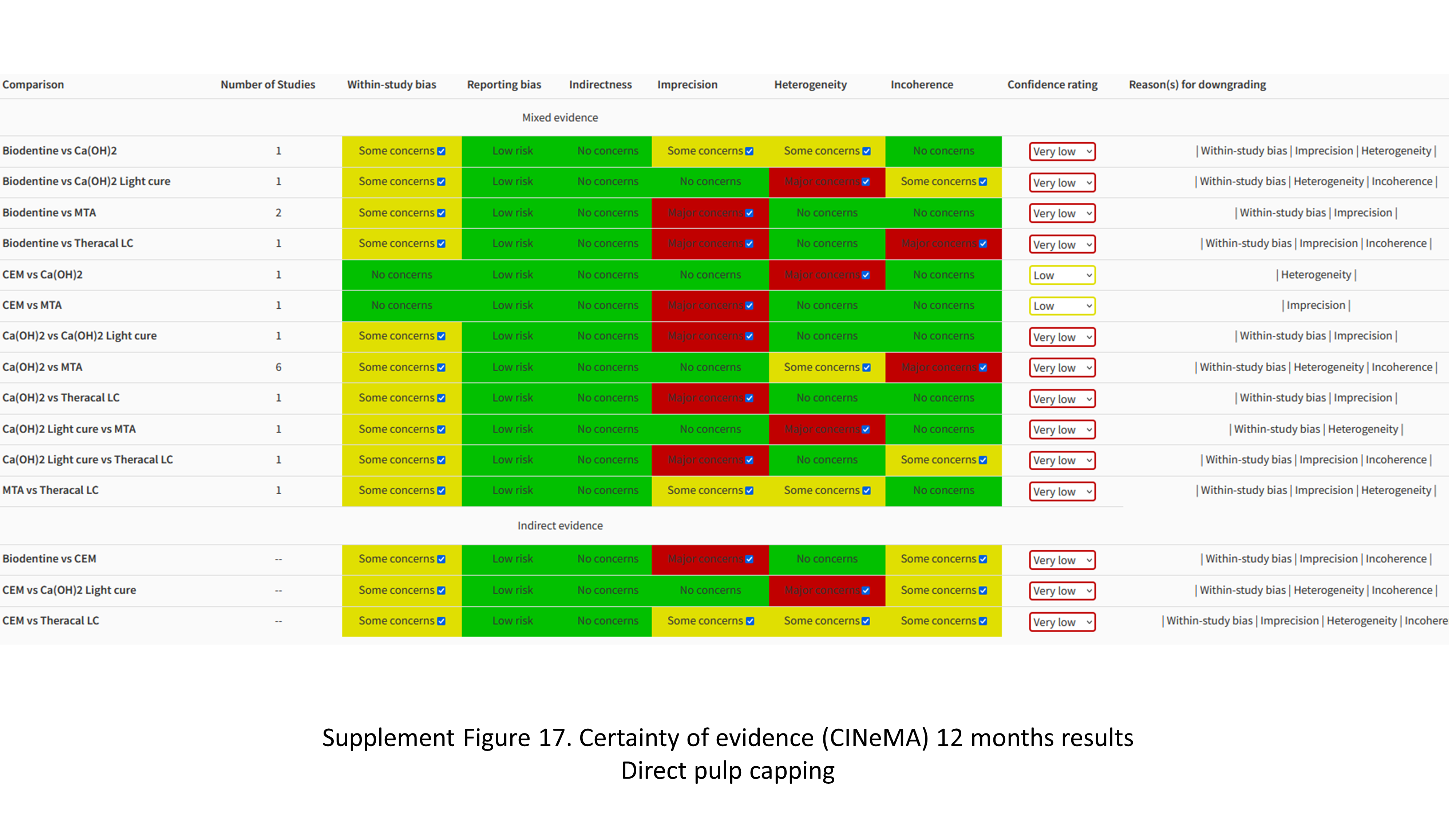

Supplement: Supplementary file 17 — Supplementary Figure 17. [file 41598_2024_69367_MOESM17_ESM.tif]

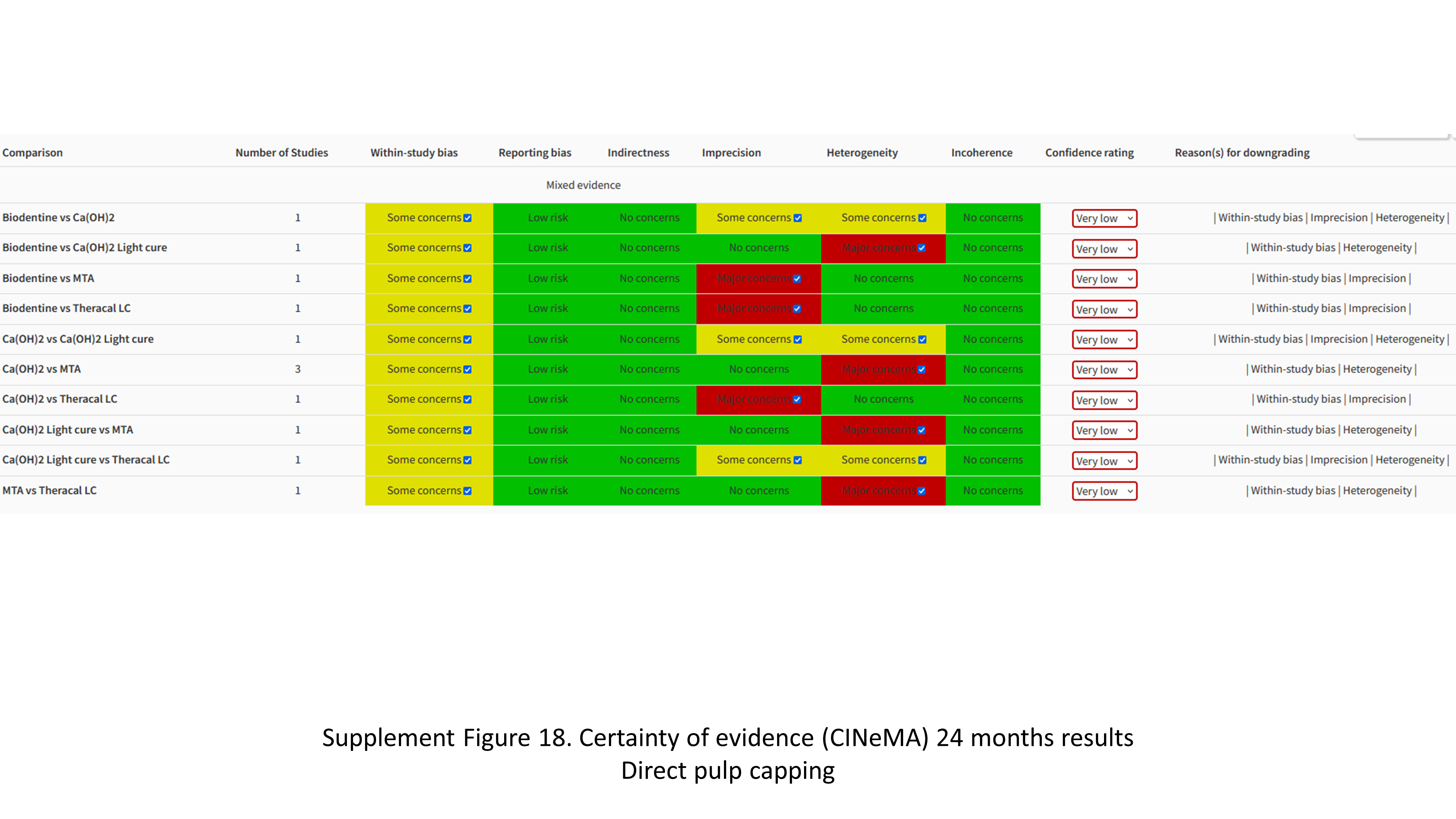

Supplement: Supplementary file 18 — Supplementary Figure 18. [file 41598_2024_69367_MOESM18_ESM.tif]

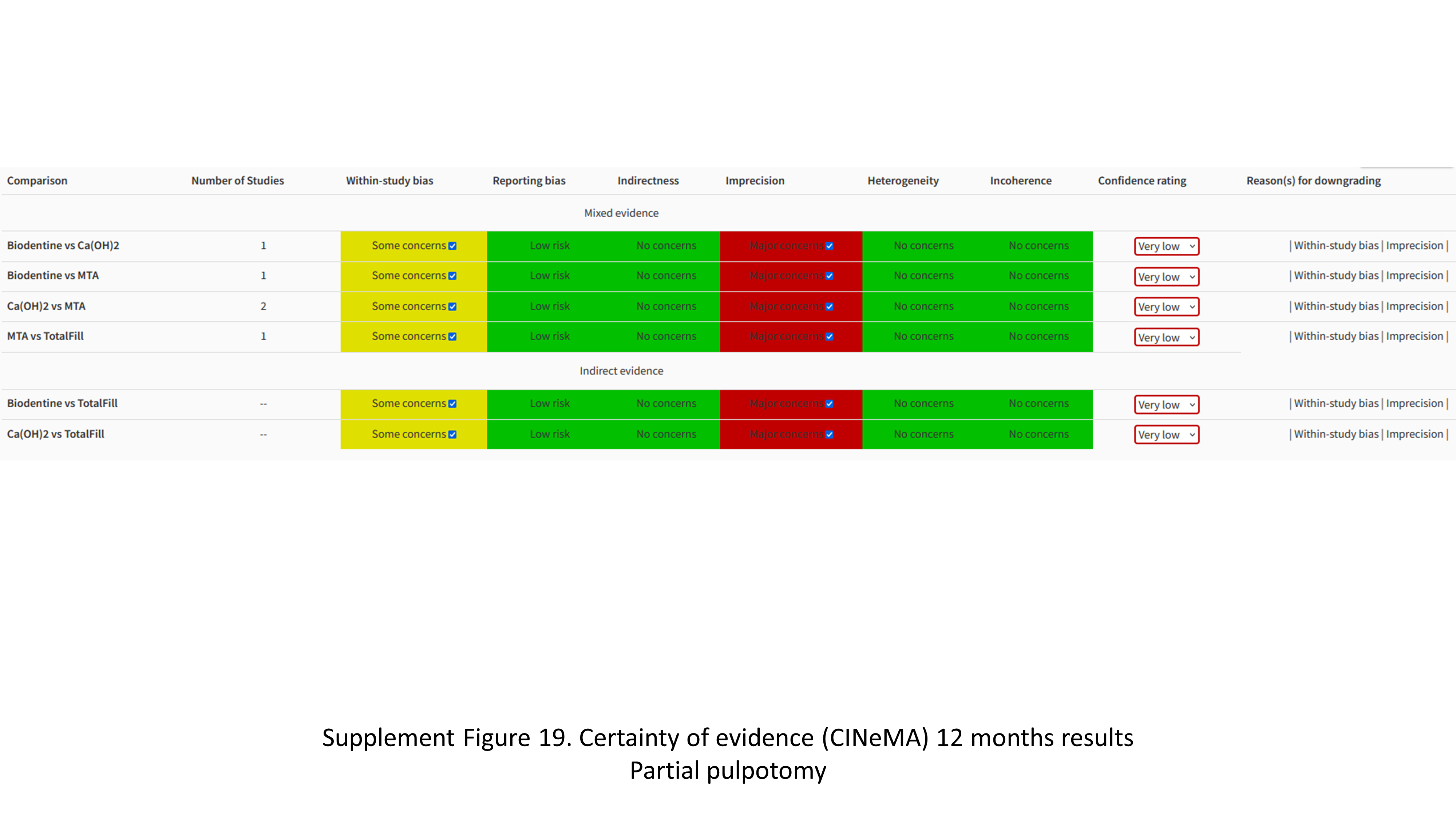

Supplement: Supplementary file 19 — Supplementary Figure 19. [file 41598_2024_69367_MOESM19_ESM.tif]

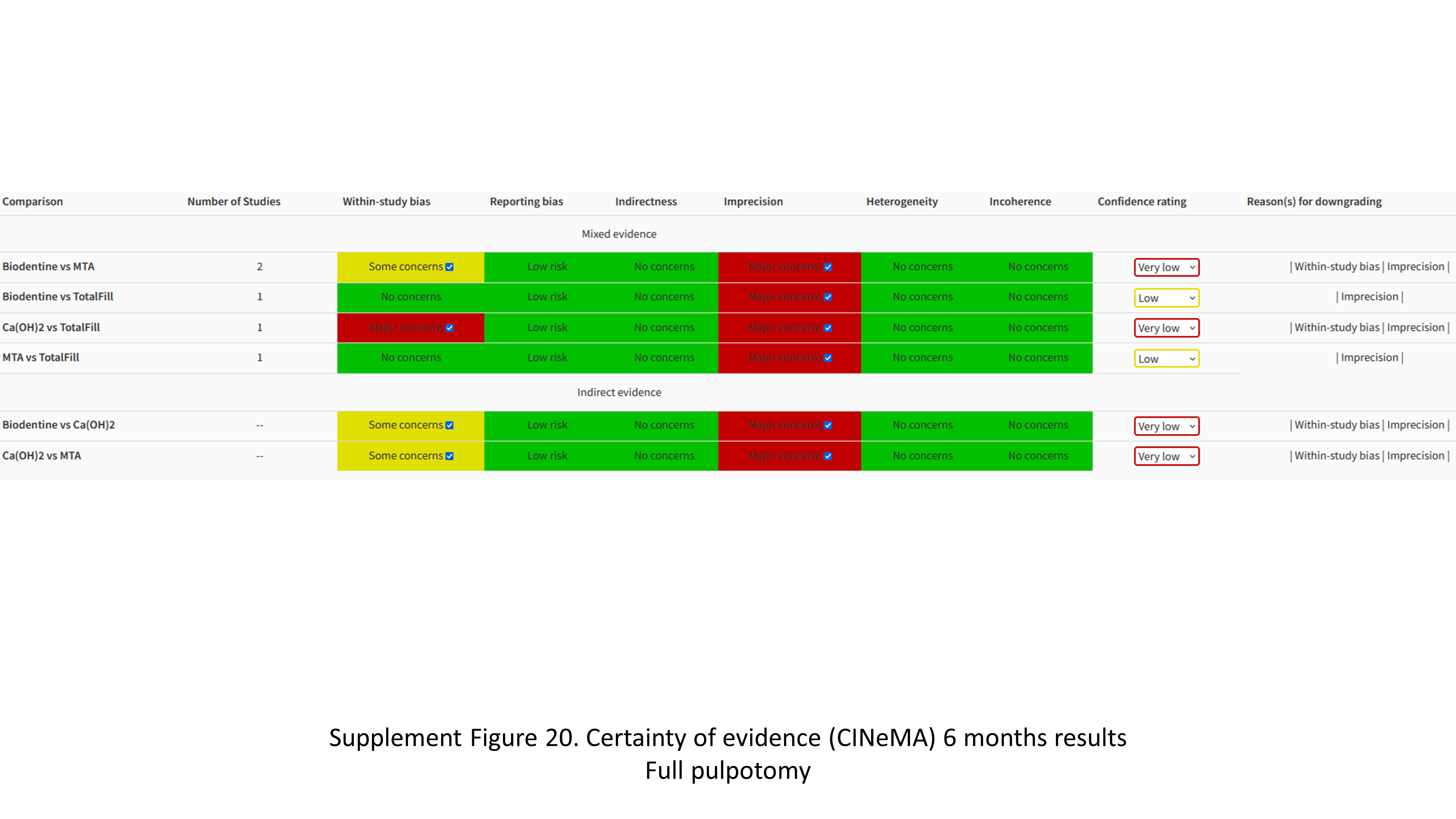

Supplement: Supplementary file 20 — Supplementary Figure 20. [file 41598_2024_69367_MOESM20_ESM.tif]

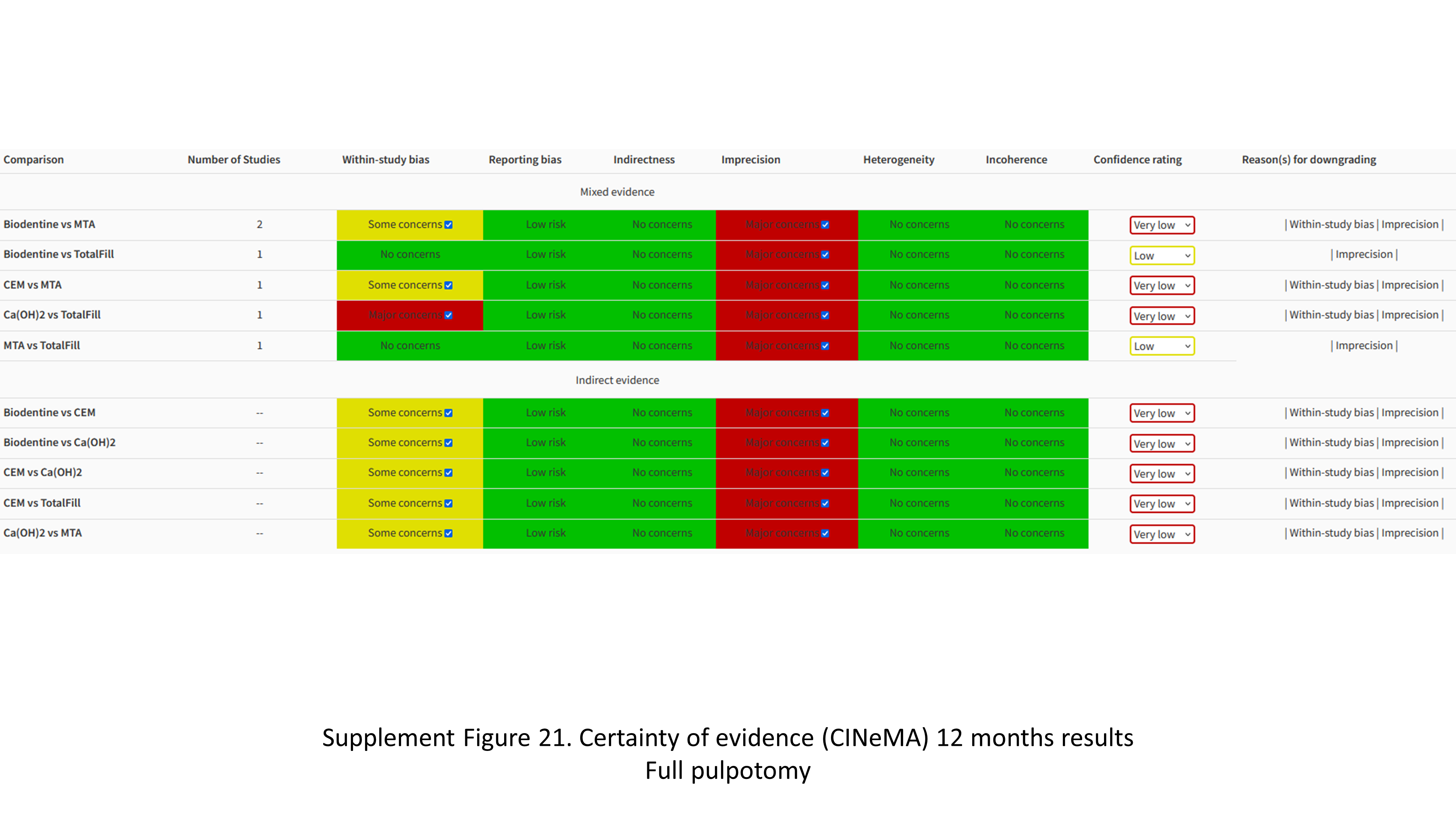

Supplement: Supplementary file 21 — Supplementary Figure 21. [file 41598_2024_69367_MOESM21_ESM.tif]

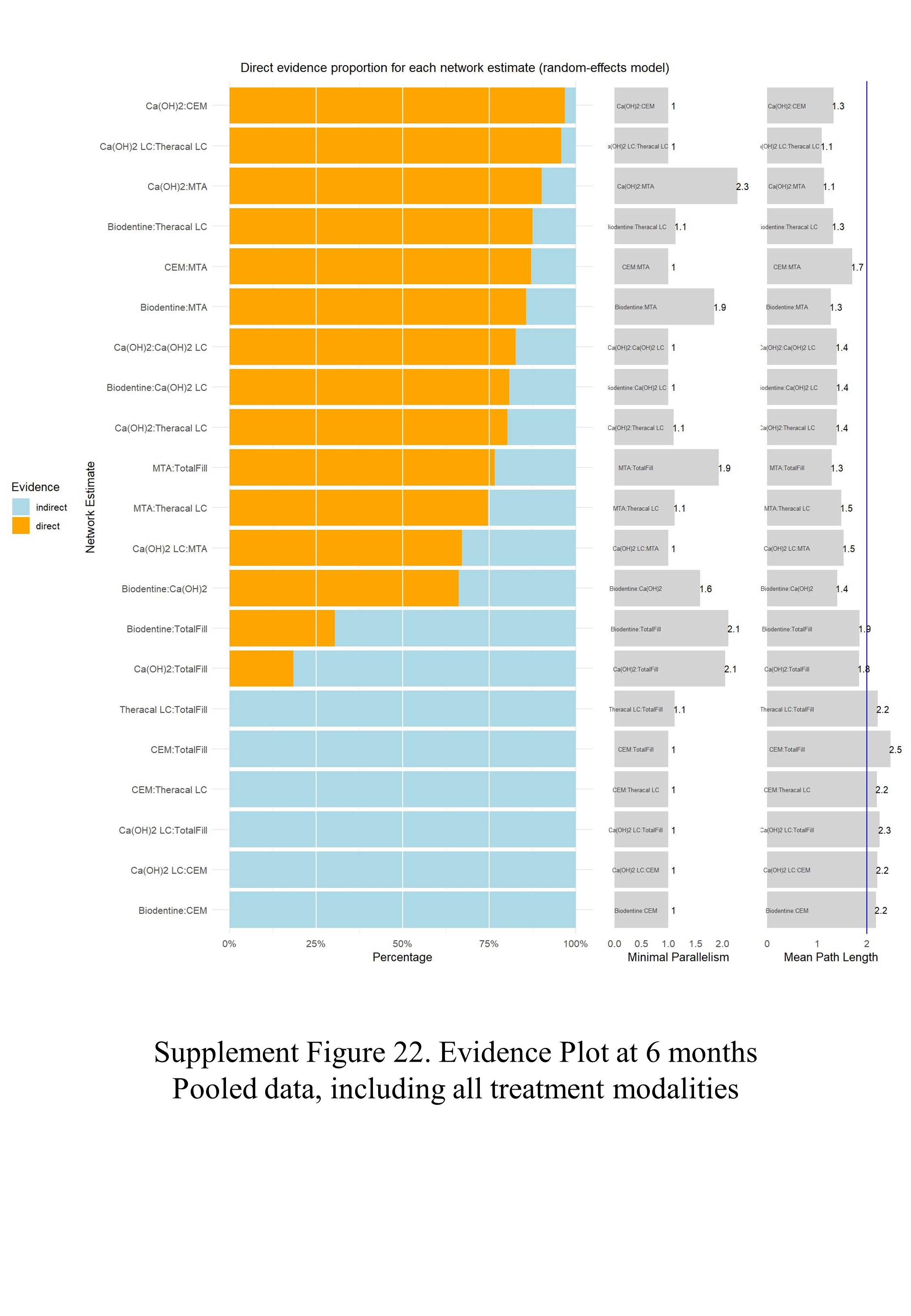

Supplement: Supplementary file 22 — Supplementary Figure 22. [file 41598_2024_69367_MOESM22_ESM.tif]

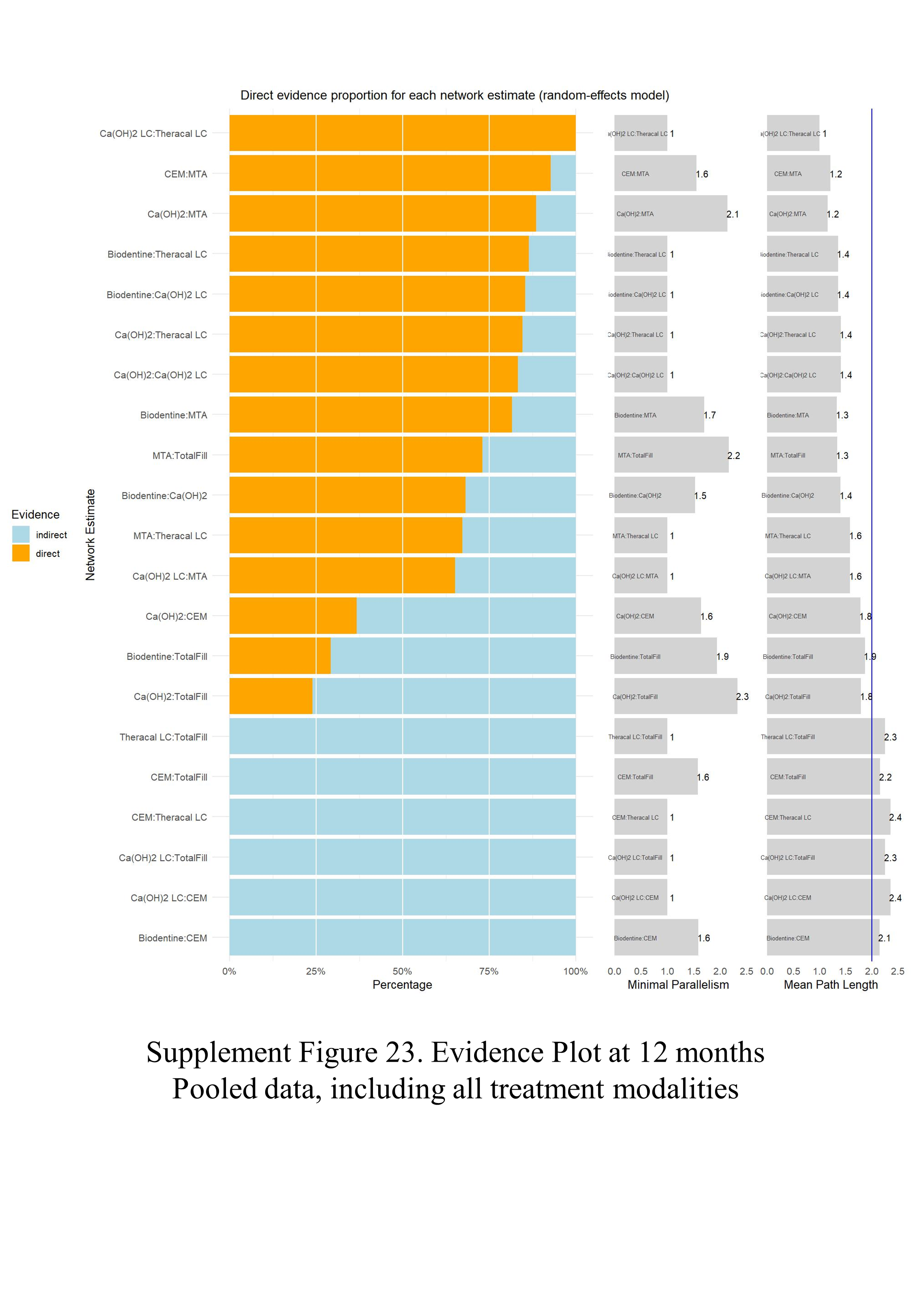

Supplement: Supplementary file 23 — Supplementary Figure 23. [file 41598_2024_69367_MOESM23_ESM.tif]

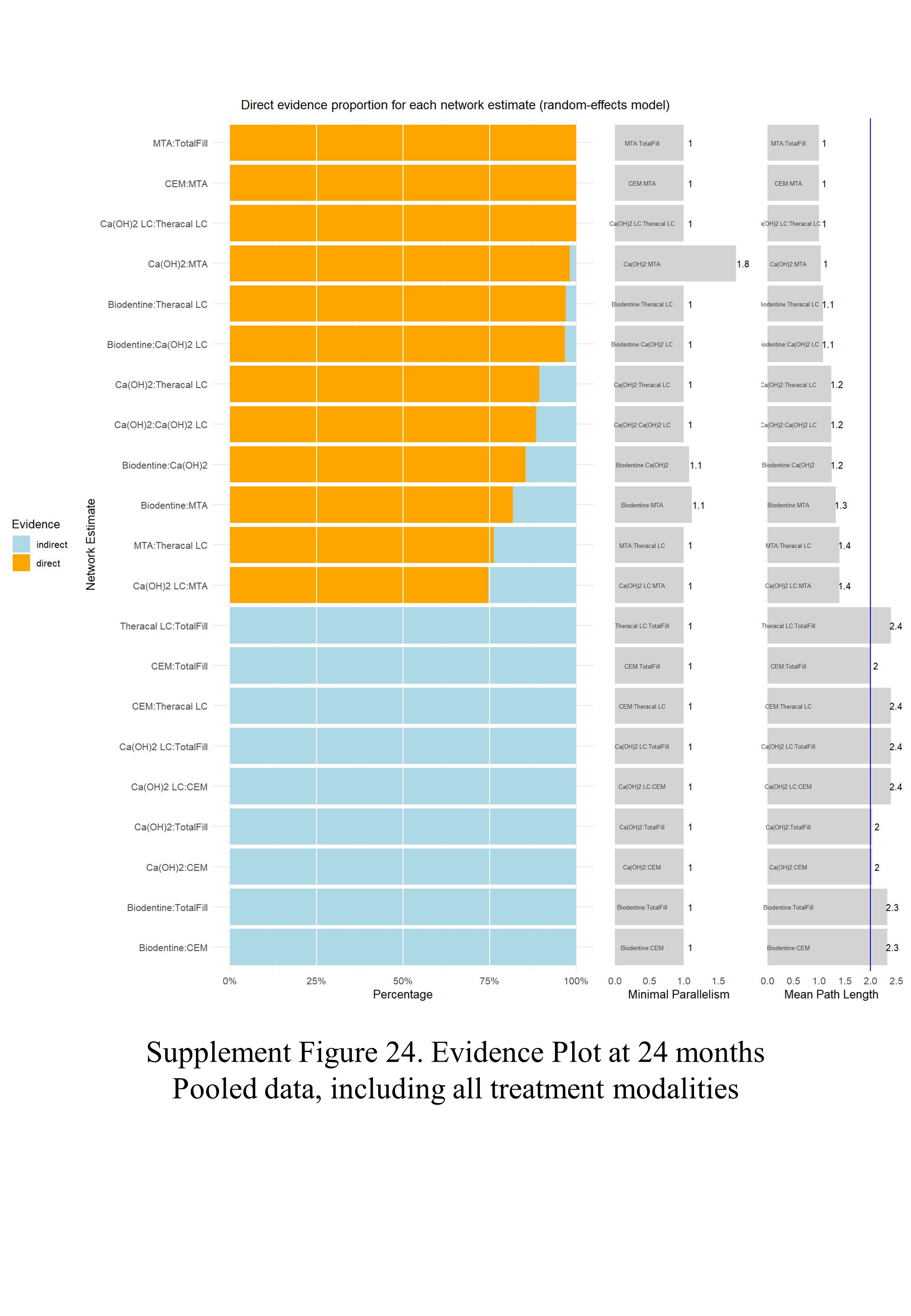

Supplement: Supplementary file 24 — Supplementary Figure 24. [file 41598_2024_69367_MOESM24_ESM.tif]

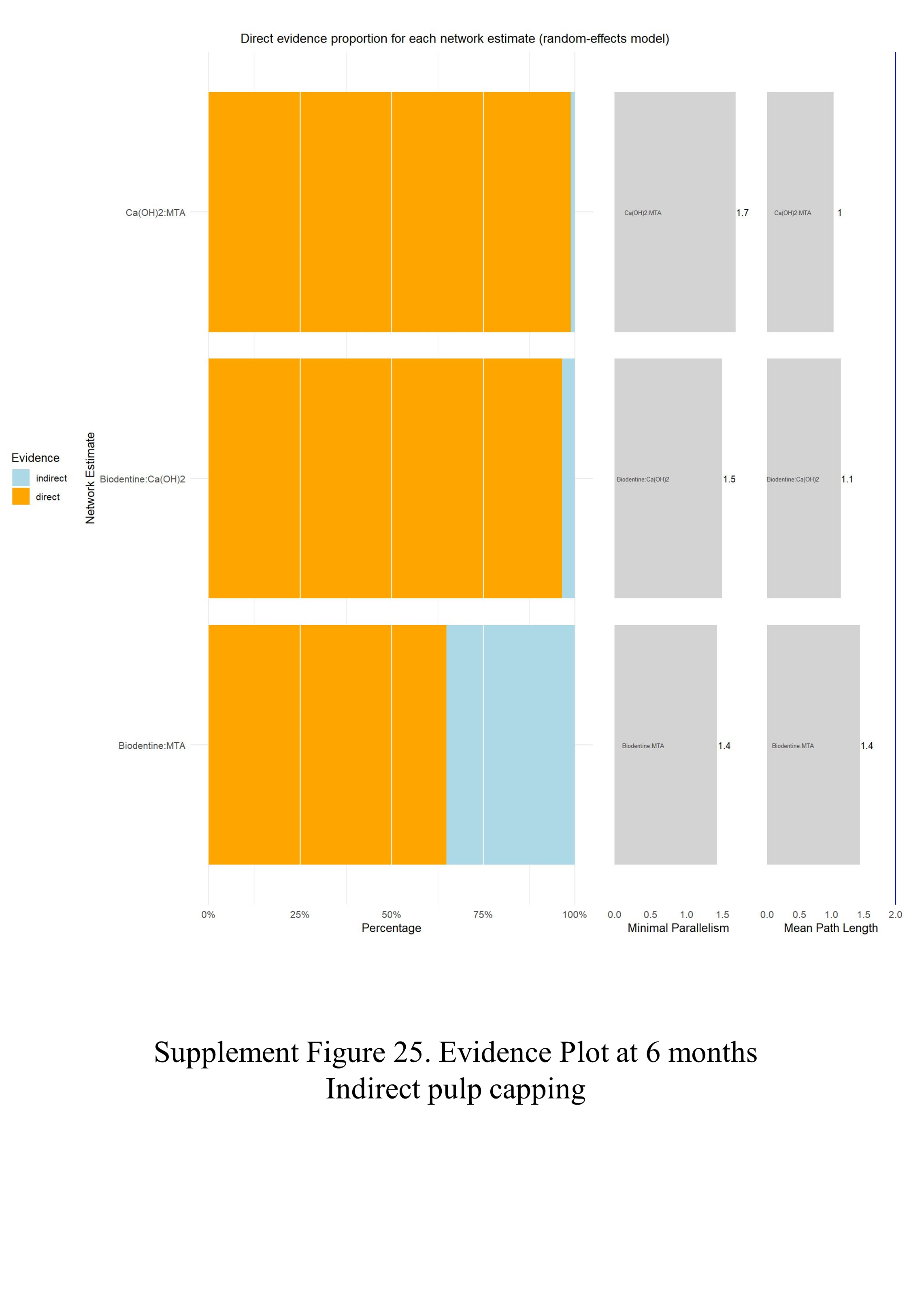

Supplement: Supplementary file 25 — Supplementary Figure 25. [file 41598_2024_69367_MOESM25_ESM.tif]

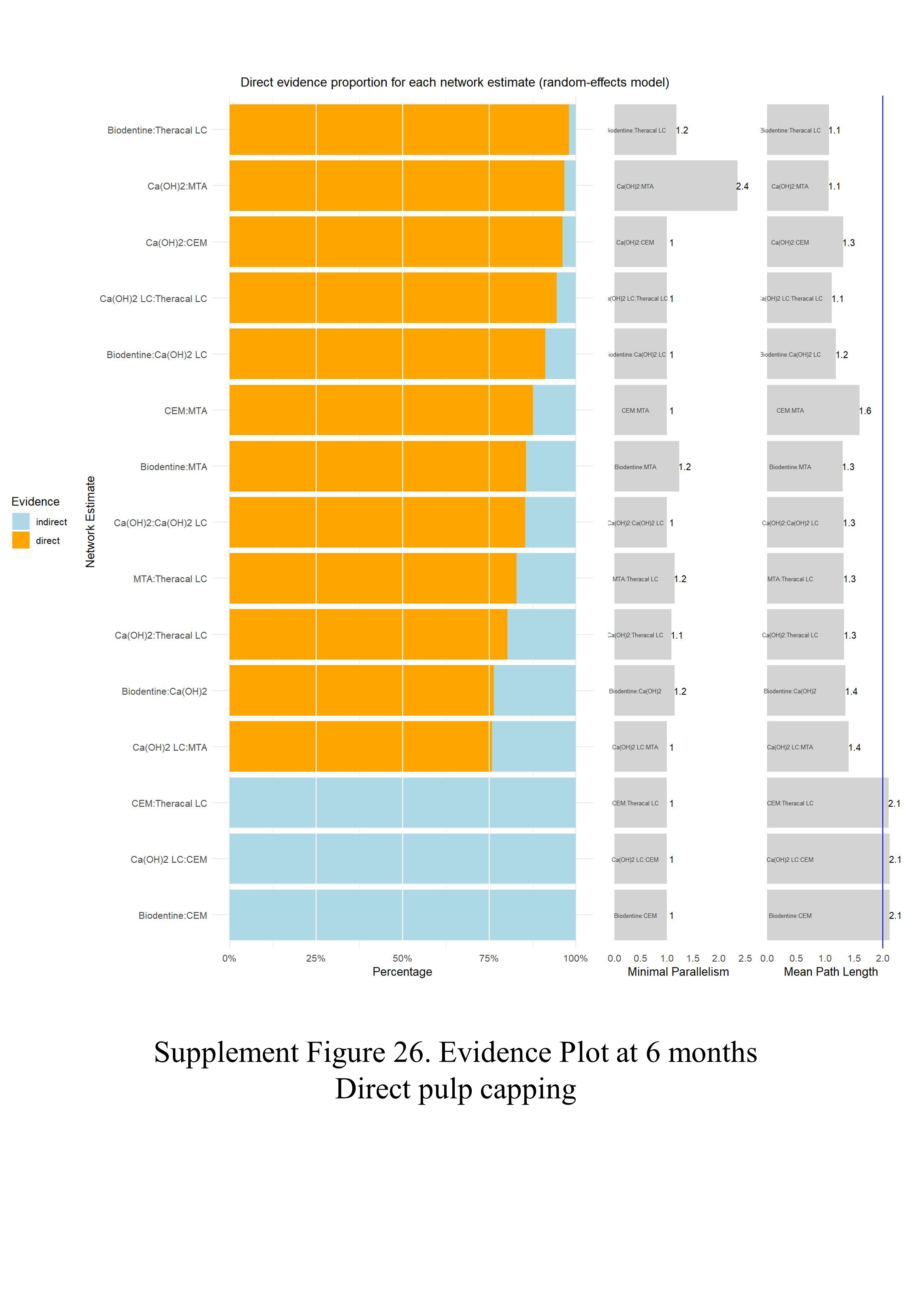

Supplement: Supplementary file 26 — Supplementary Figure 26. [file 41598_2024_69367_MOESM26_ESM.tif]

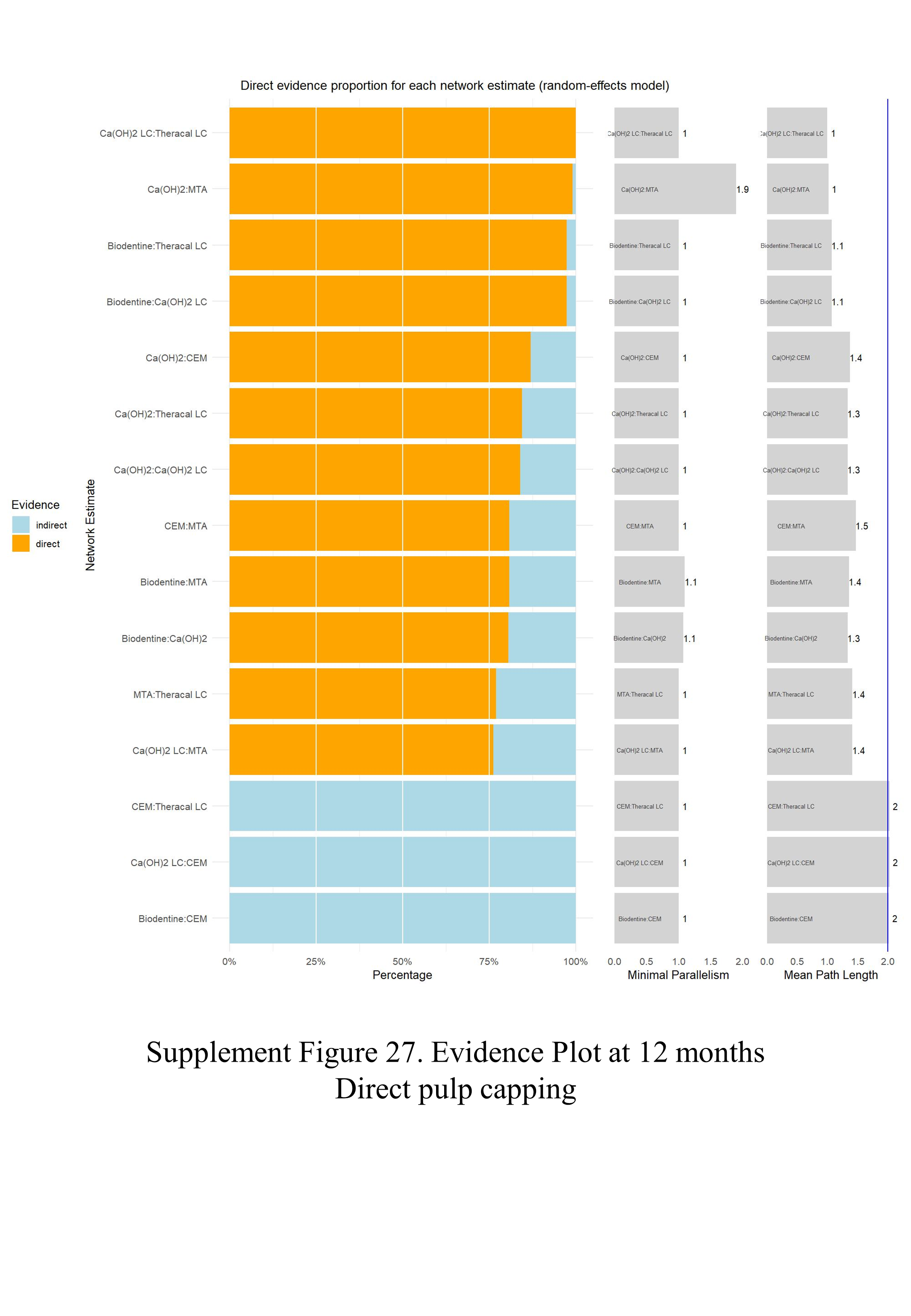

Supplement: Supplementary file 27 — Supplementary Figure 27. [file 41598_2024_69367_MOESM27_ESM.tif]

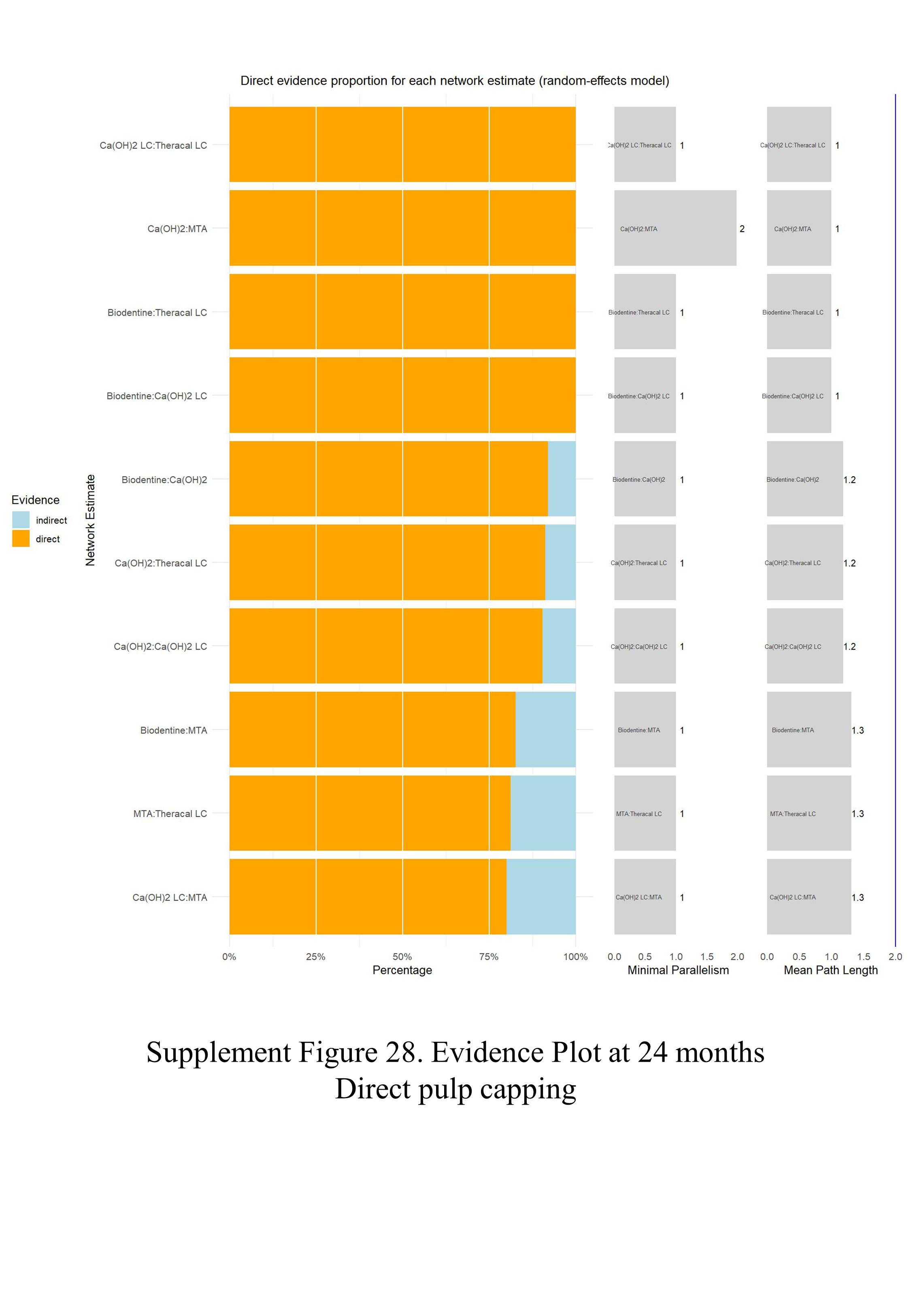

Supplement: Supplementary file 28 — Supplementary Figure 28. [file 41598_2024_69367_MOESM28_ESM.tif]

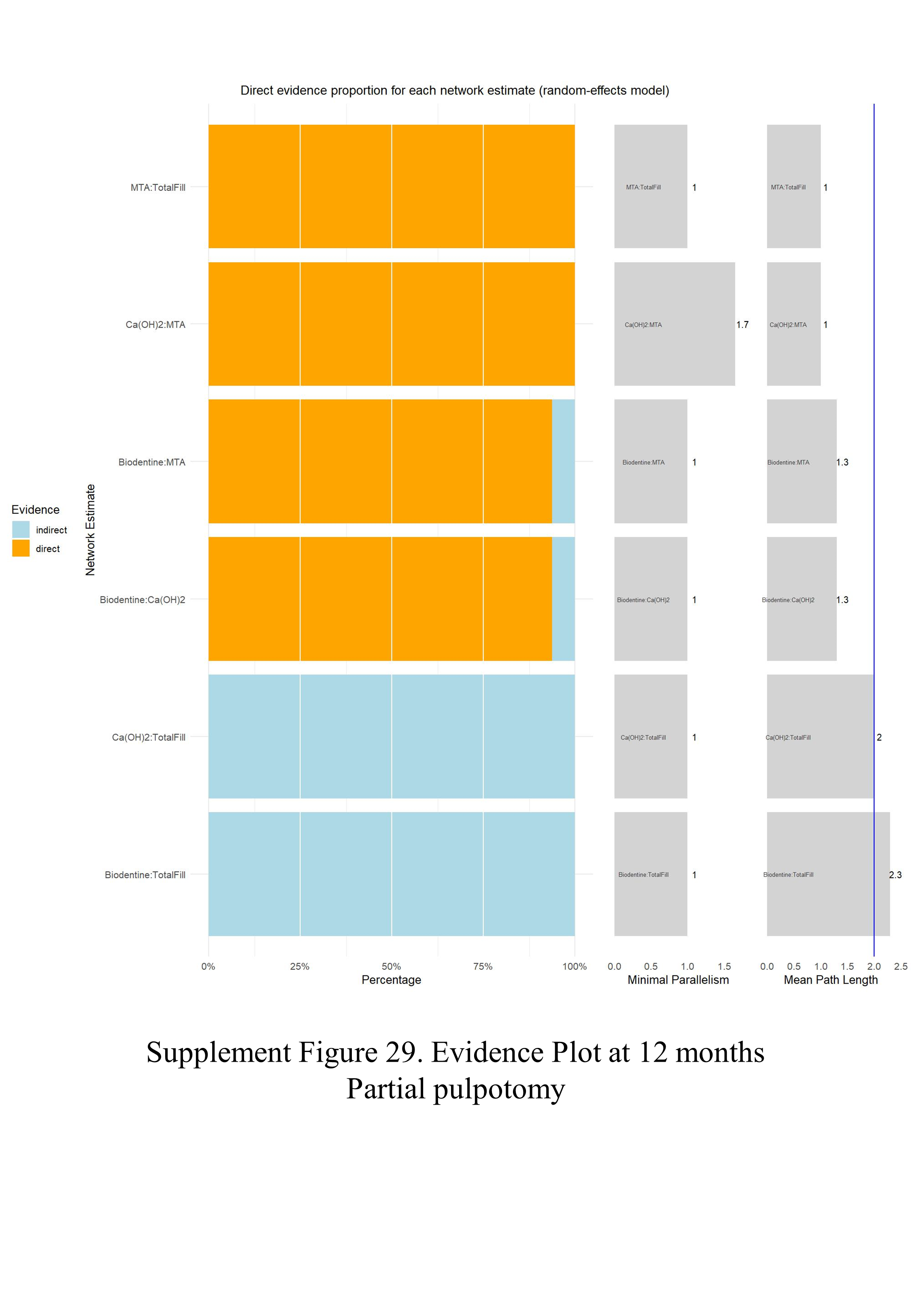

Supplement: Supplementary file 29 — Supplementary Figure 29. [file 41598_2024_69367_MOESM29_ESM.tif]

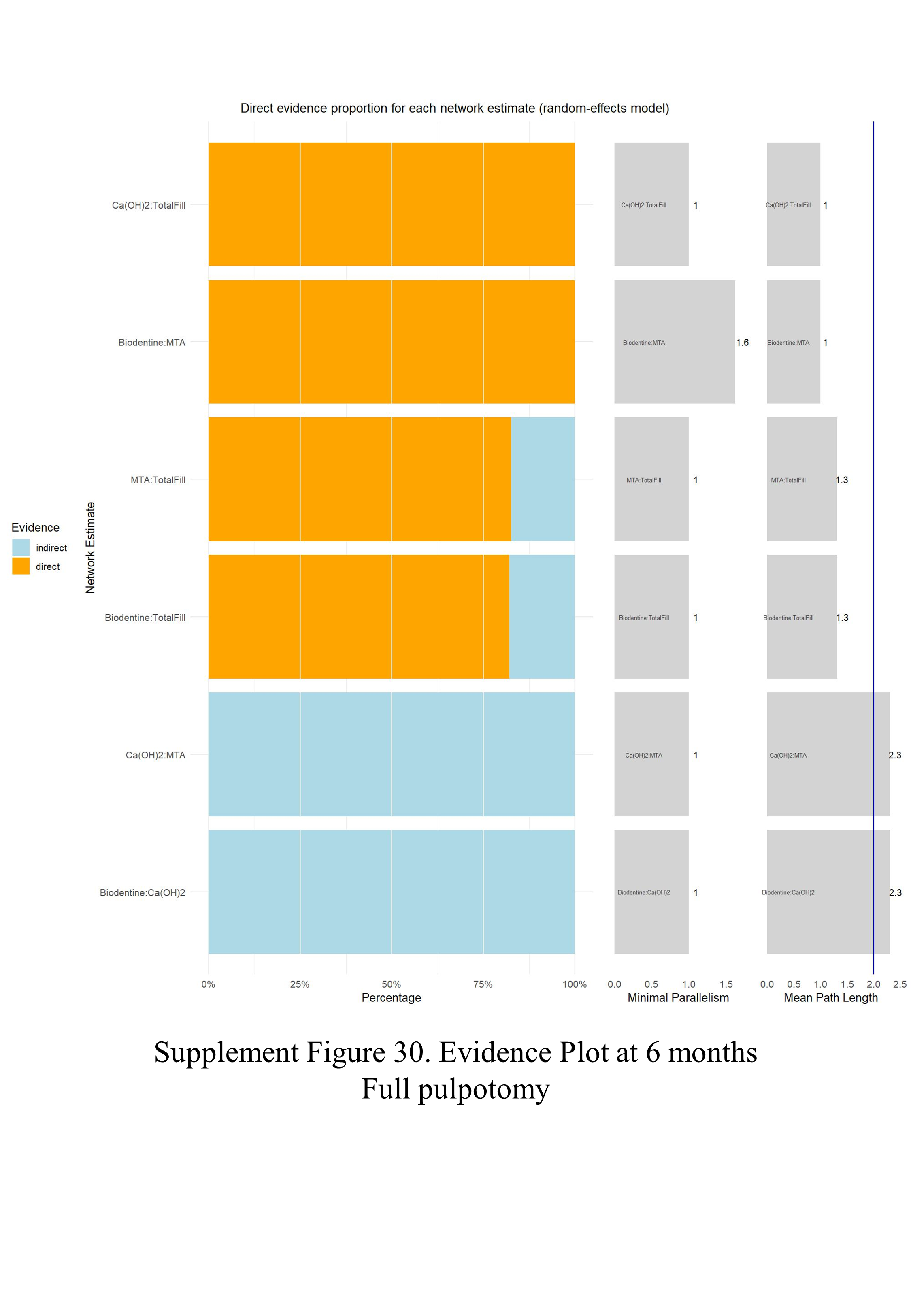

Supplement: Supplementary file 30 — Supplementary Figure 30. [file 41598_2024_69367_MOESM30_ESM.tif]

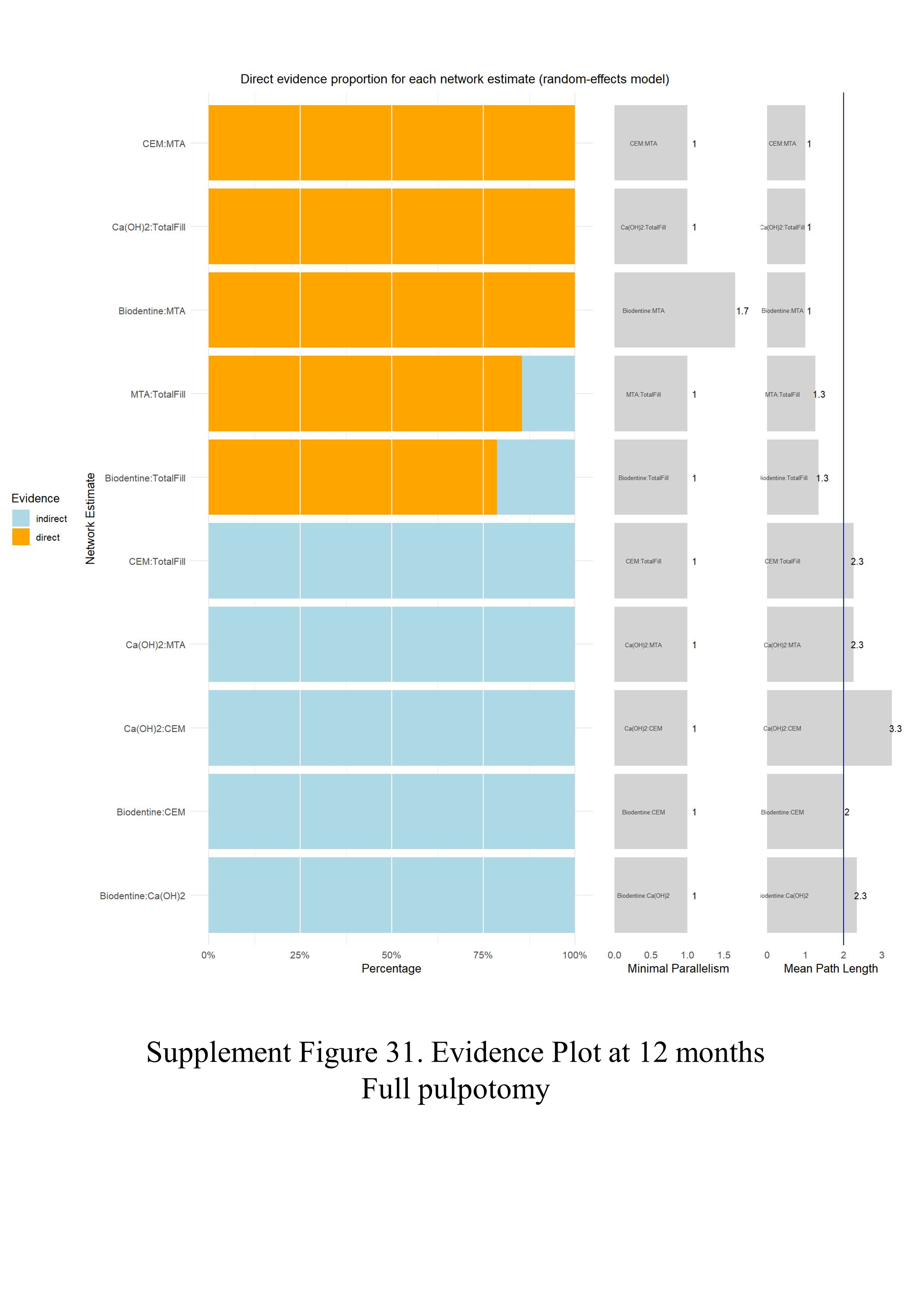

Supplement: Supplementary file 31 — Supplementary Figure 31. [file 41598_2024_69367_MOESM31_ESM.tif]

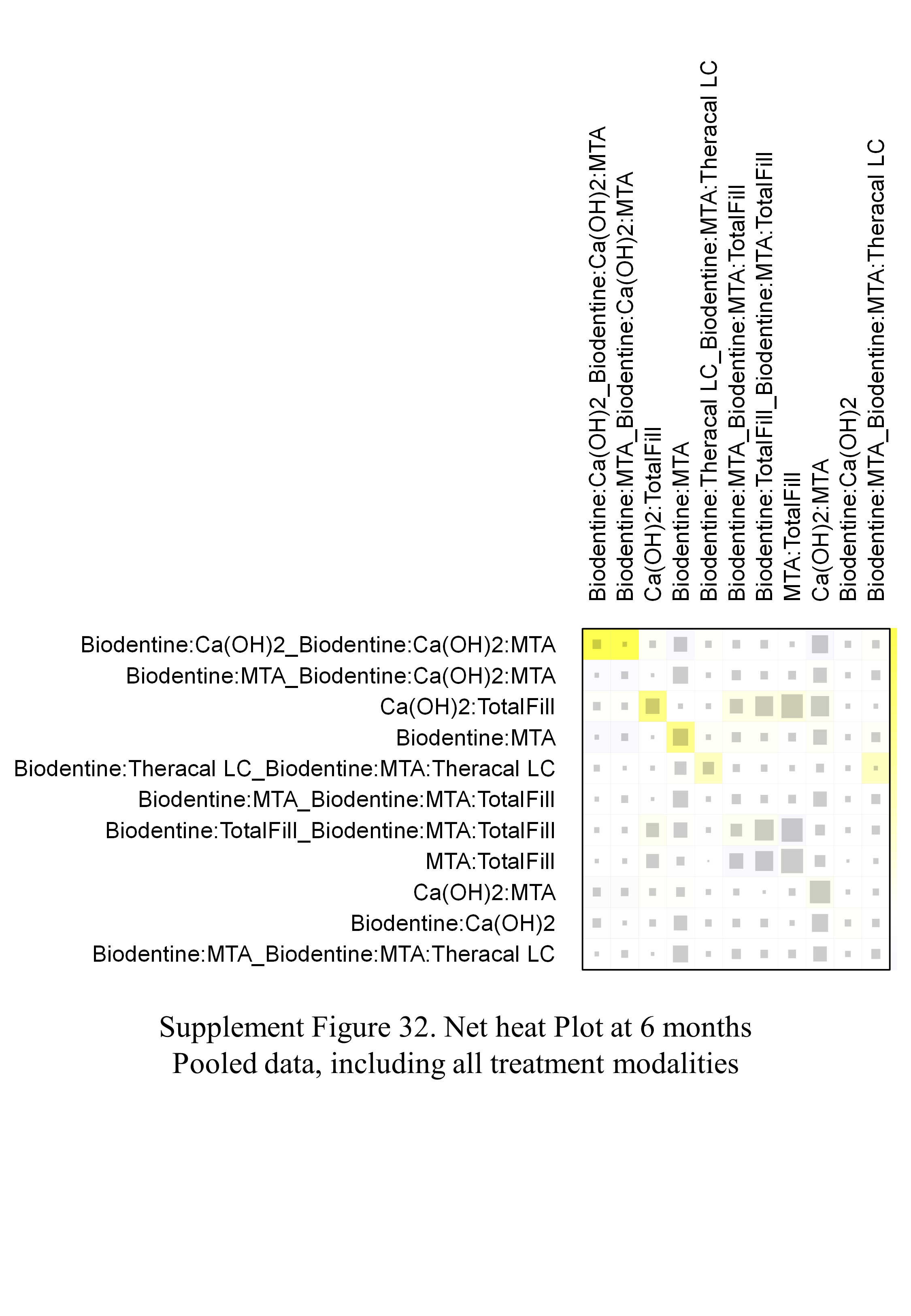

Supplement: Supplementary file 32 — Supplementary Figure 32. [file 41598_2024_69367_MOESM32_ESM.tif]

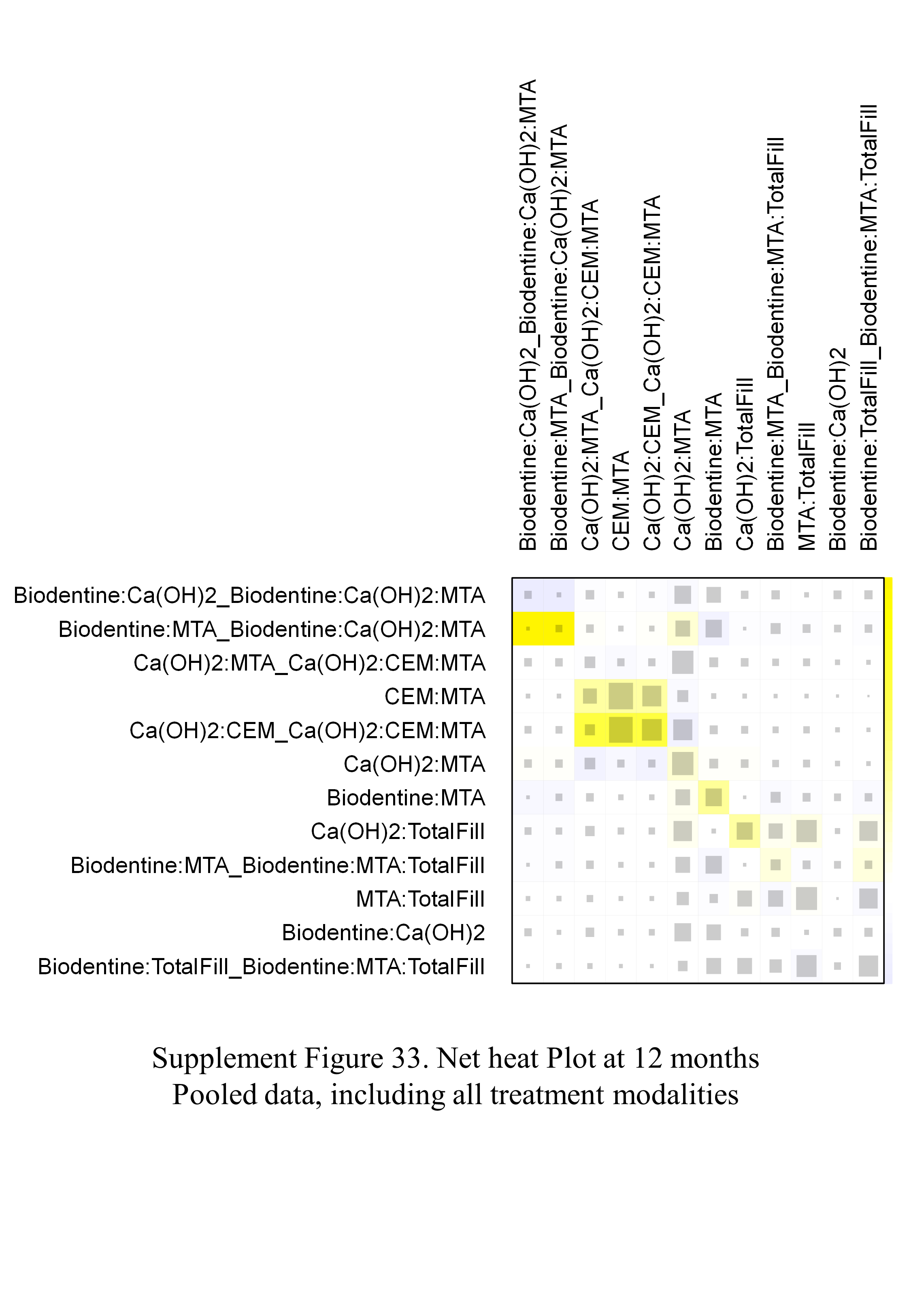

Supplement: Supplementary file 33 — Supplementary Figure 33. [file 41598_2024_69367_MOESM33_ESM.tif]

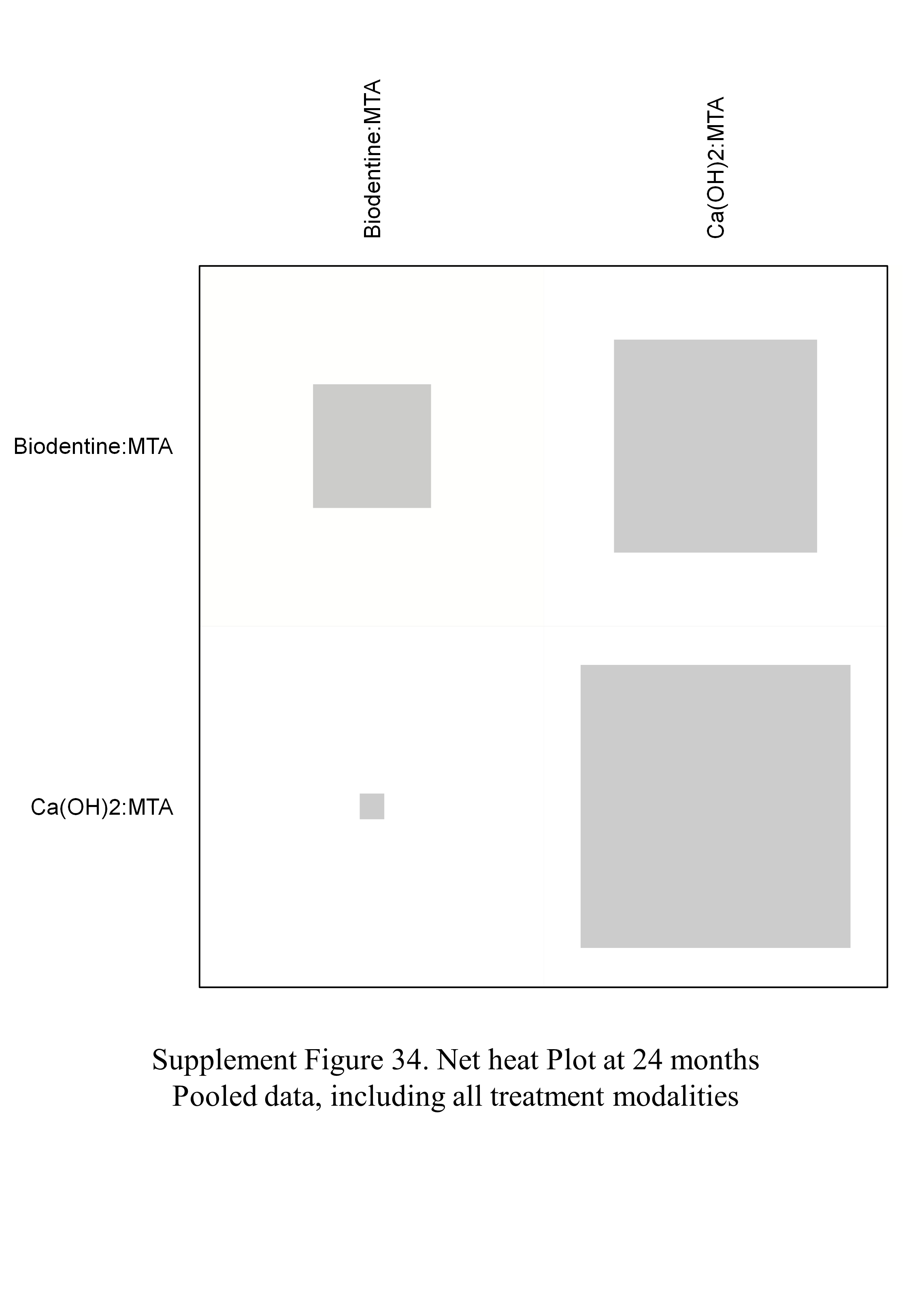

Supplement: Supplementary file 34 — Supplementary Figure 34. [file 41598_2024_69367_MOESM34_ESM.tif]

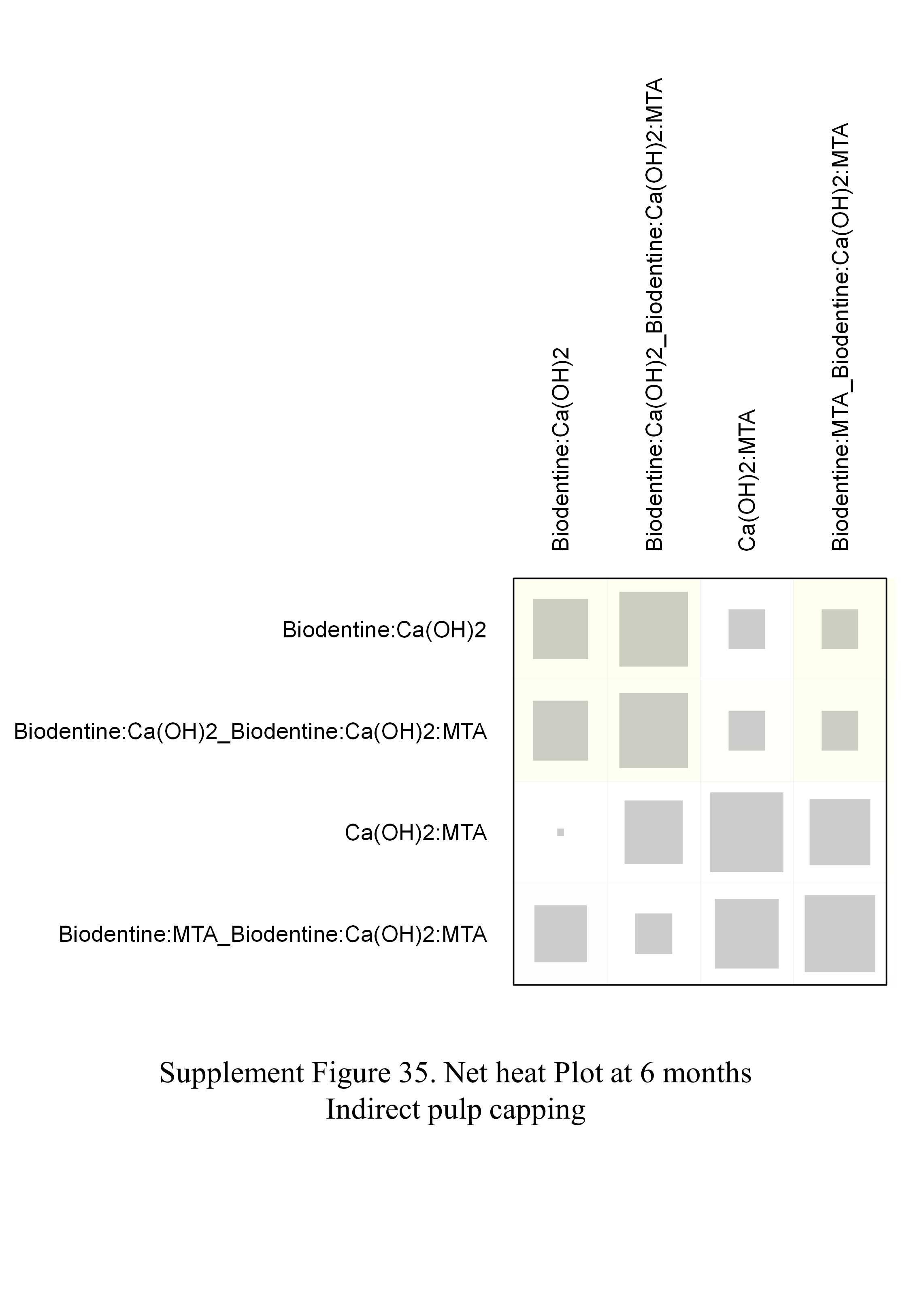

Supplement: Supplementary file 35 — Supplementary Figure 35. [file 41598_2024_69367_MOESM35_ESM.tif]

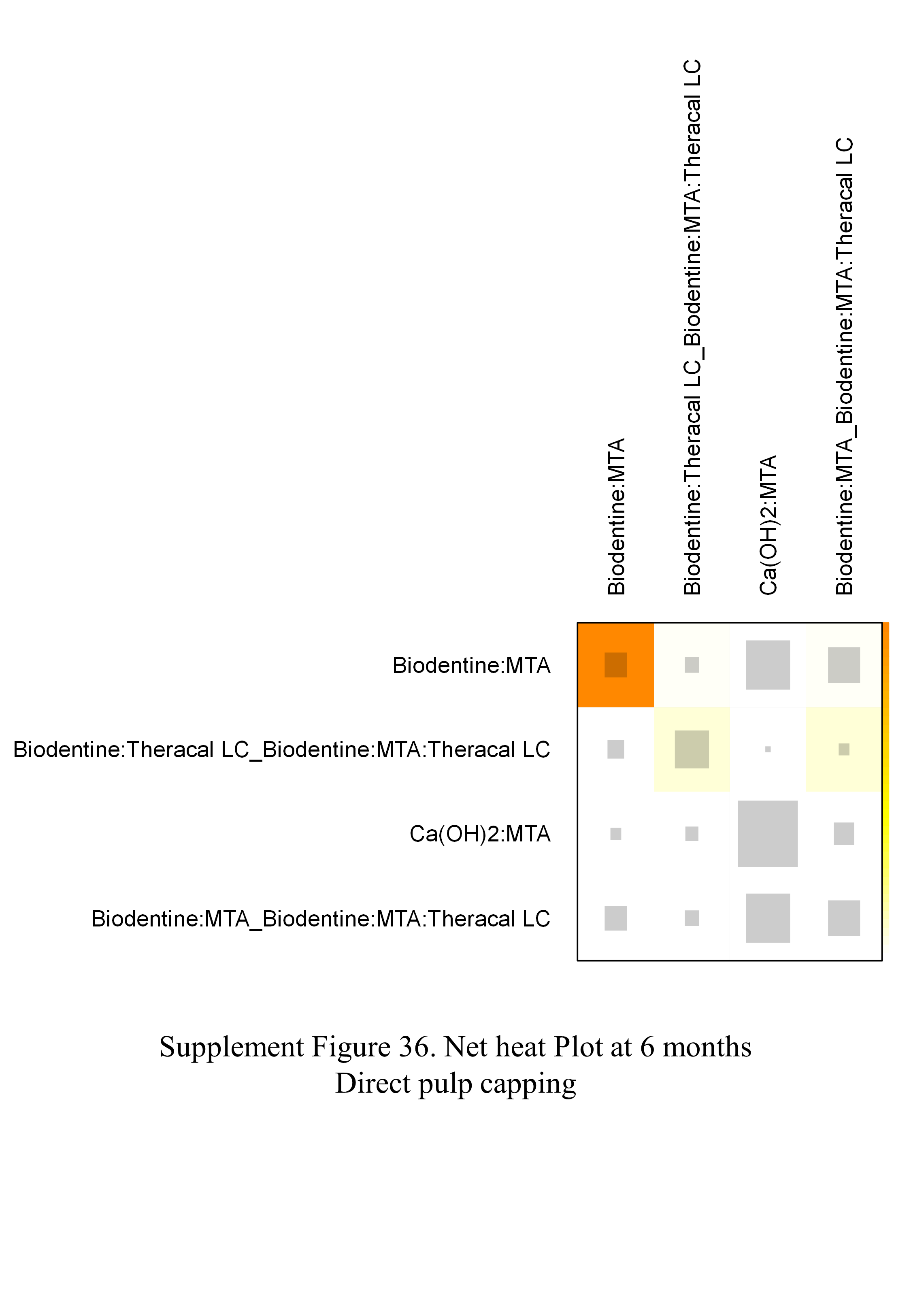

Supplement: Supplementary file 36 — Supplementary Figure 36. [file 41598_2024_69367_MOESM36_ESM.tif]

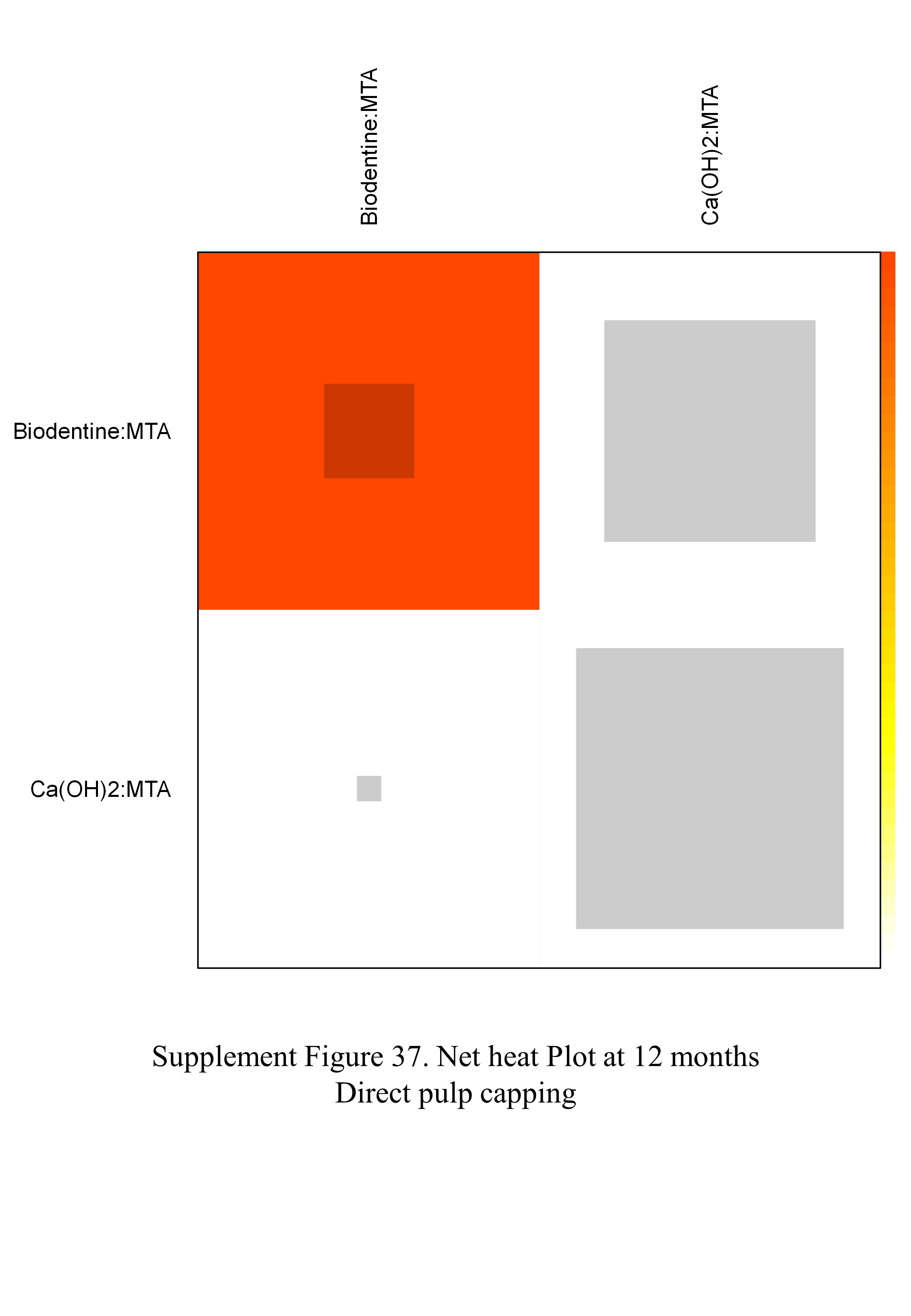

Supplement: Supplementary file 37 — Supplementary Figure 37. [file 41598_2024_69367_MOESM37_ESM.tif]

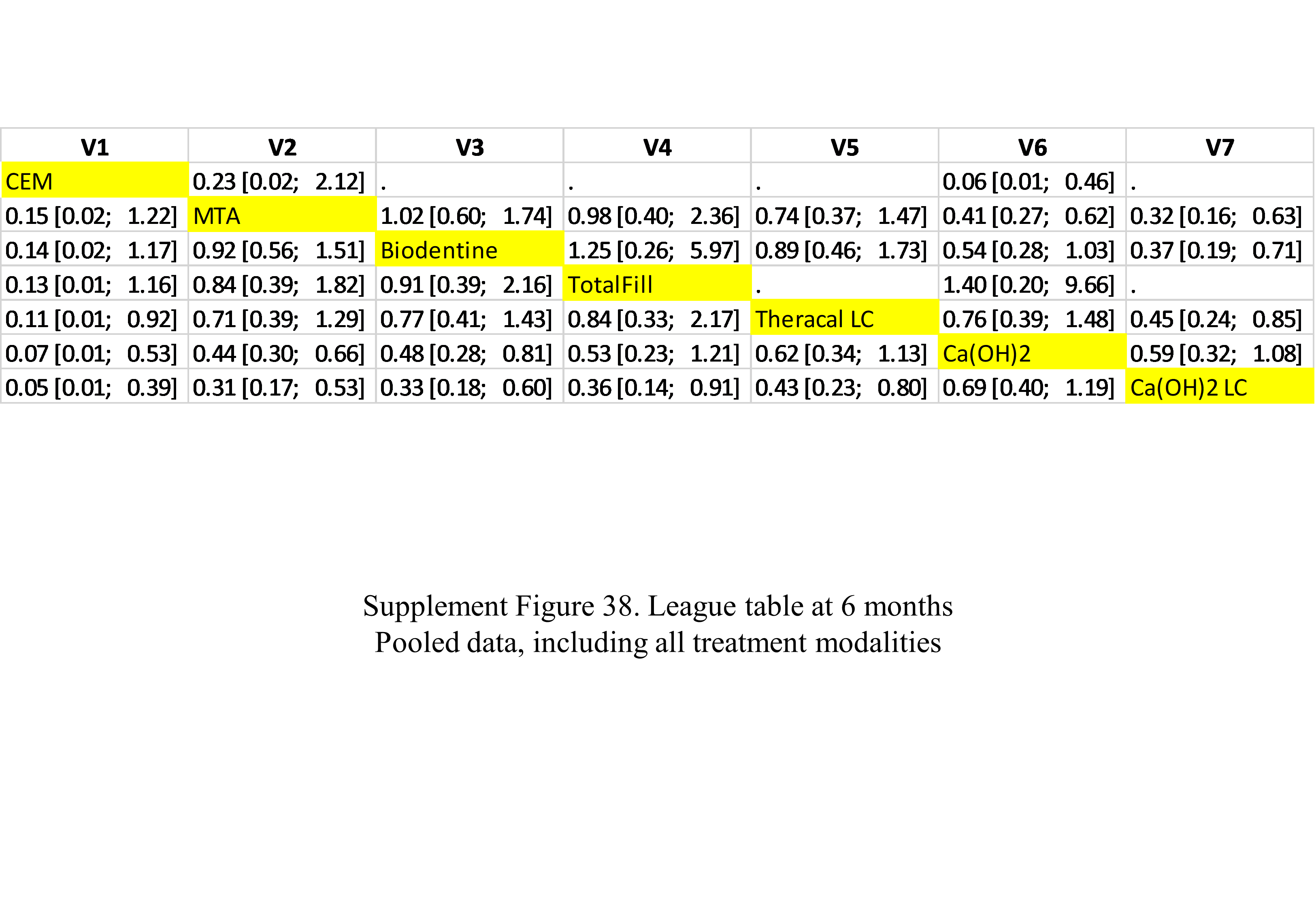

Supplement: Supplementary file 38 — Supplementary Figure 38. [file 41598_2024_69367_MOESM38_ESM.tif]

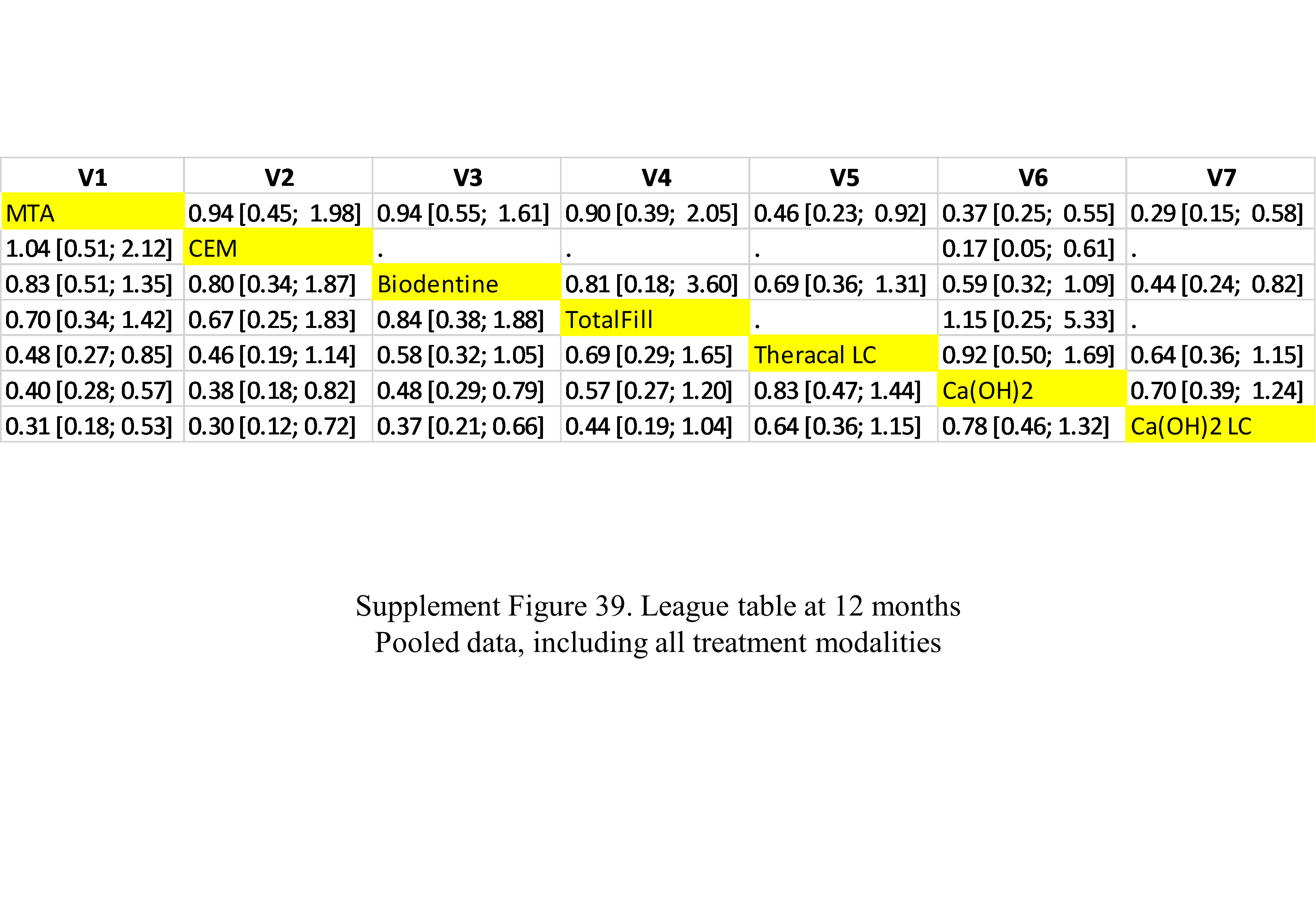

Supplement: Supplementary file 39 — Supplementary Figure 39. [file 41598_2024_69367_MOESM39_ESM.tif]

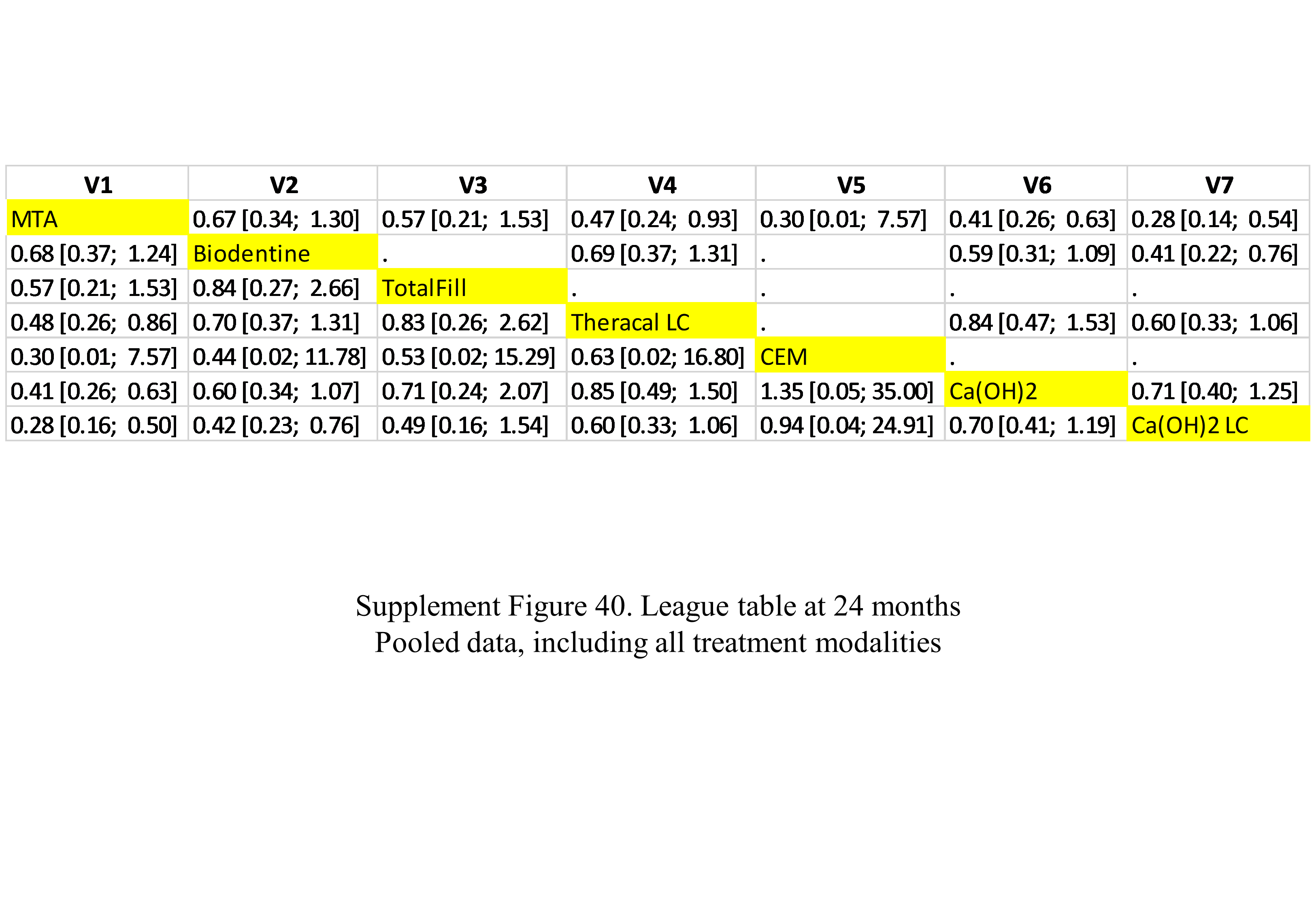

Supplement: Supplementary file 40 — Supplementary Figure 40. [file 41598_2024_69367_MOESM40_ESM.tif]

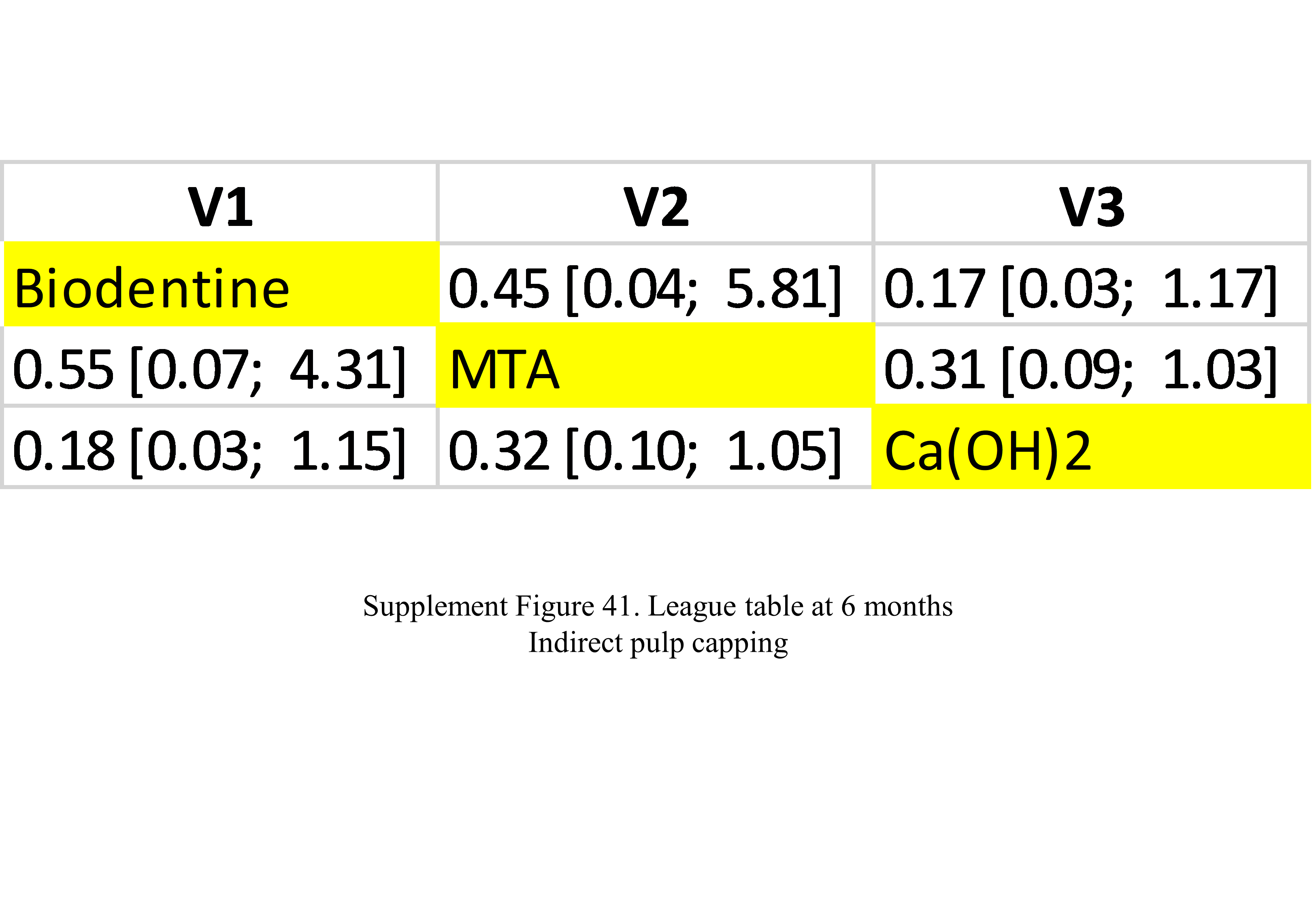

Supplement: Supplementary file 41 — Supplementary Figure 41. [file 41598_2024_69367_MOESM41_ESM.tif]

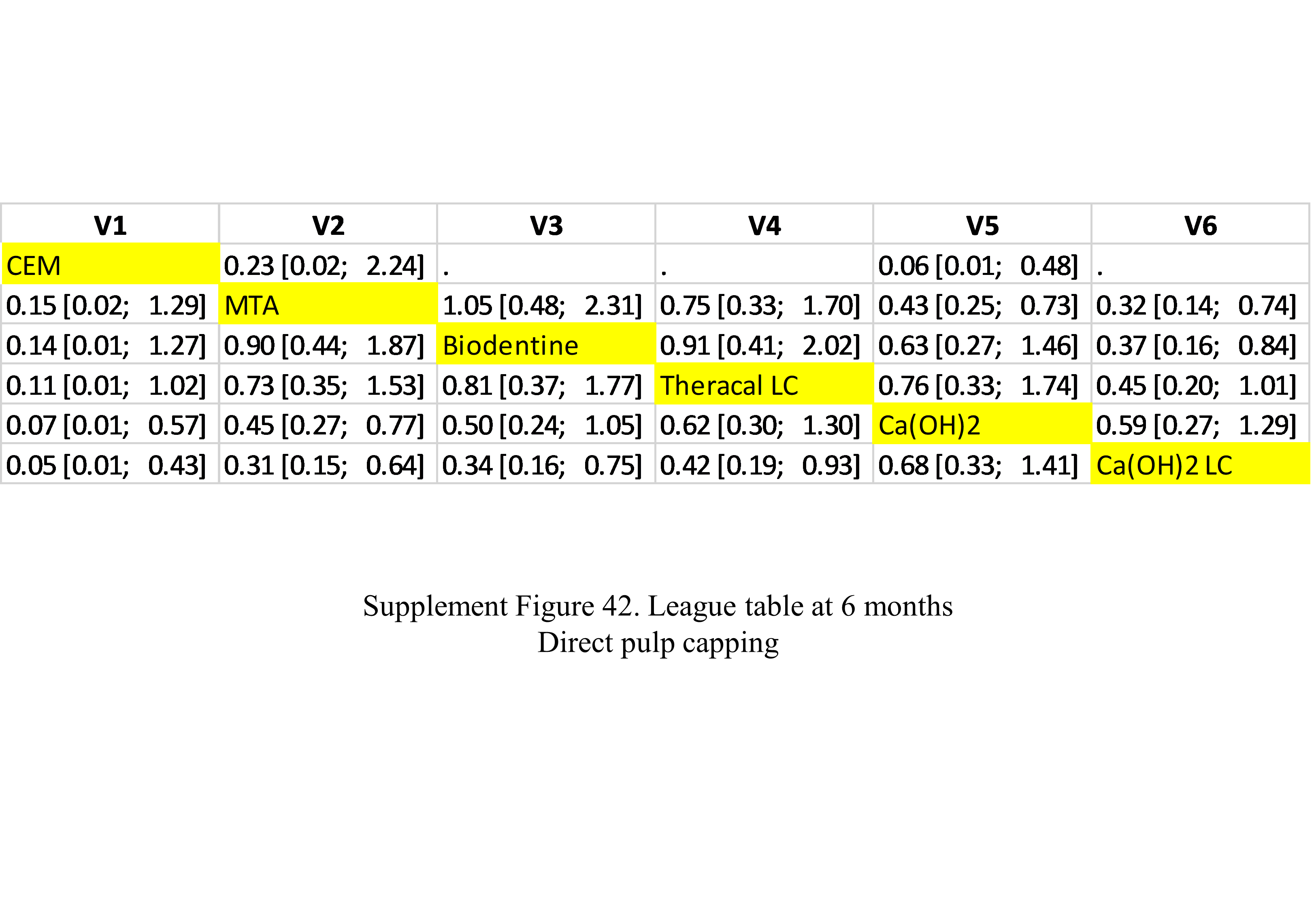

Supplement: Supplementary file 42 — Supplementary Figure 42. [file 41598_2024_69367_MOESM42_ESM.tif]

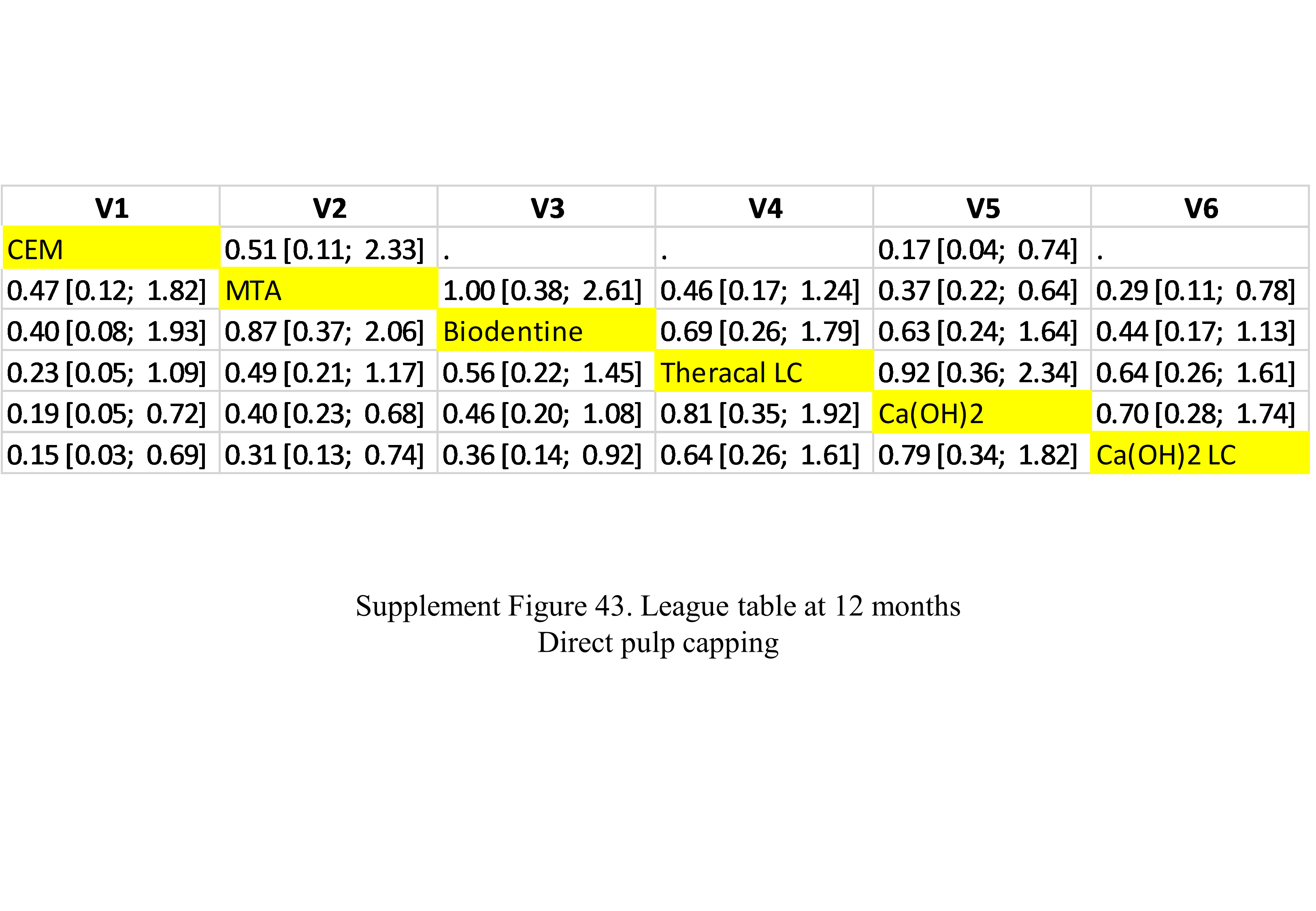

Supplement: Supplementary file 43 — Supplementary Figure 43. [file 41598_2024_69367_MOESM43_ESM.tif]

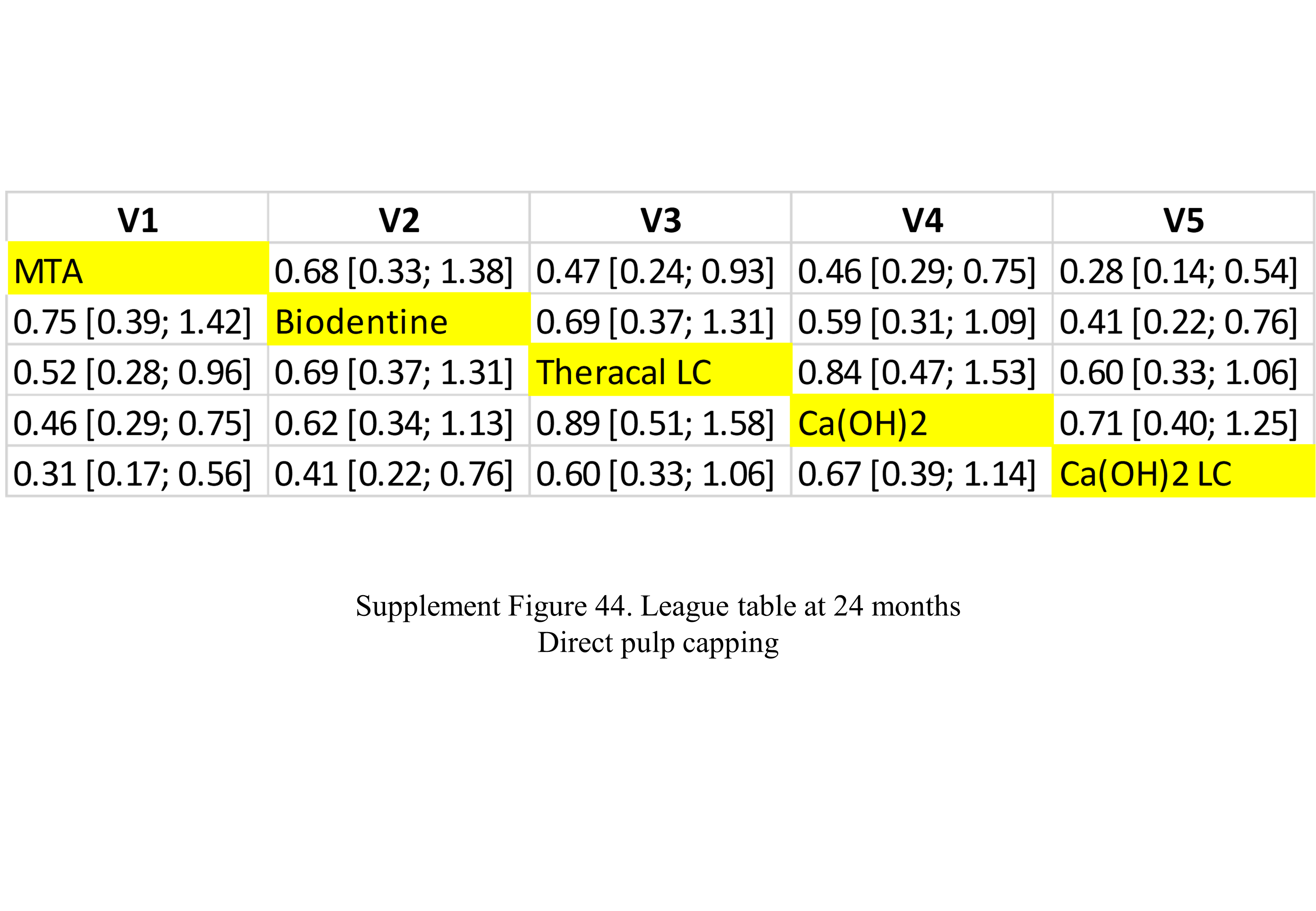

Supplement: Supplementary file 44 — Supplementary Figure 44. [file 41598_2024_69367_MOESM44_ESM.tif]

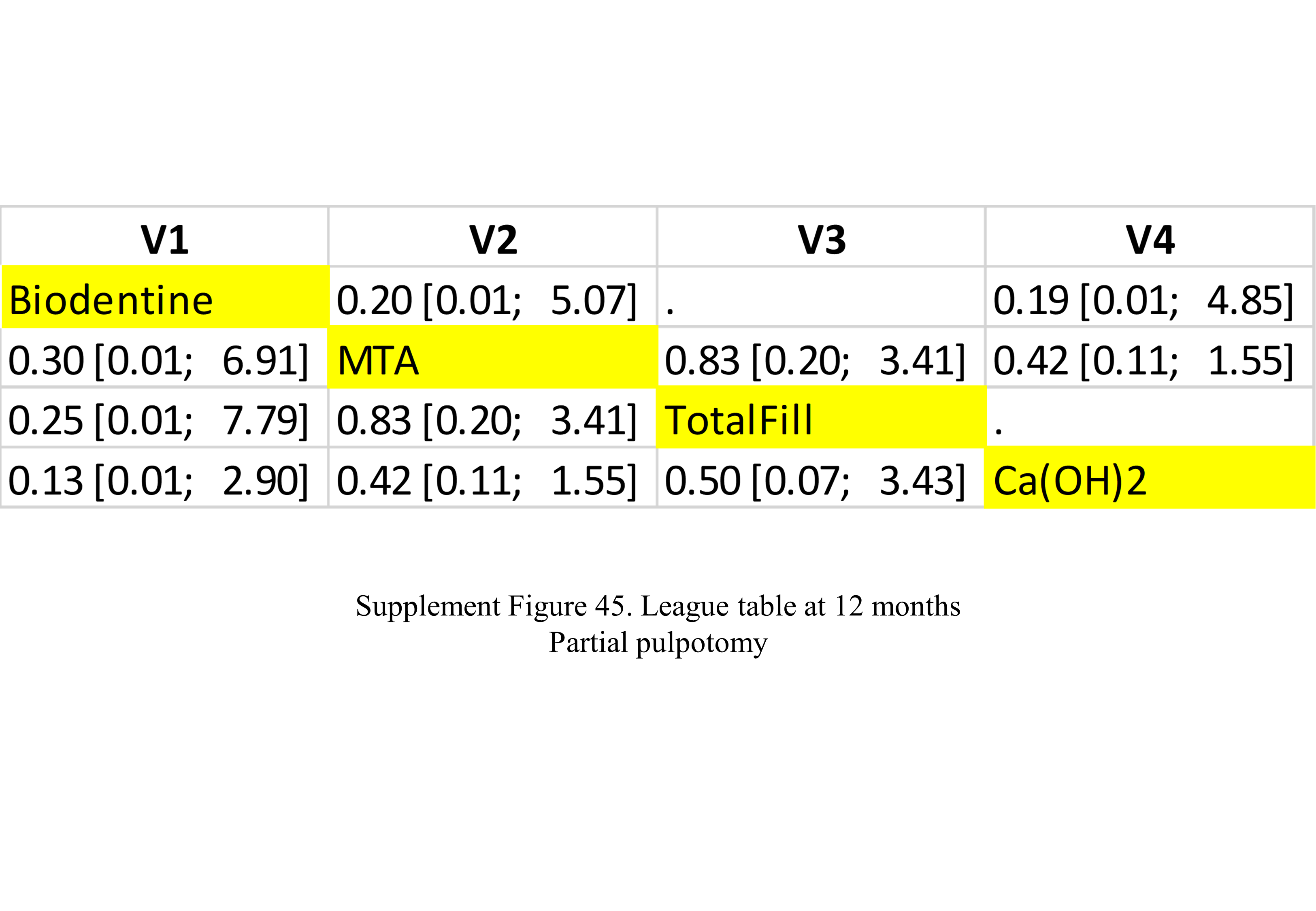

Supplement: Supplementary file 45 — Supplementary Figure 45. [file 41598_2024_69367_MOESM45_ESM.tif]

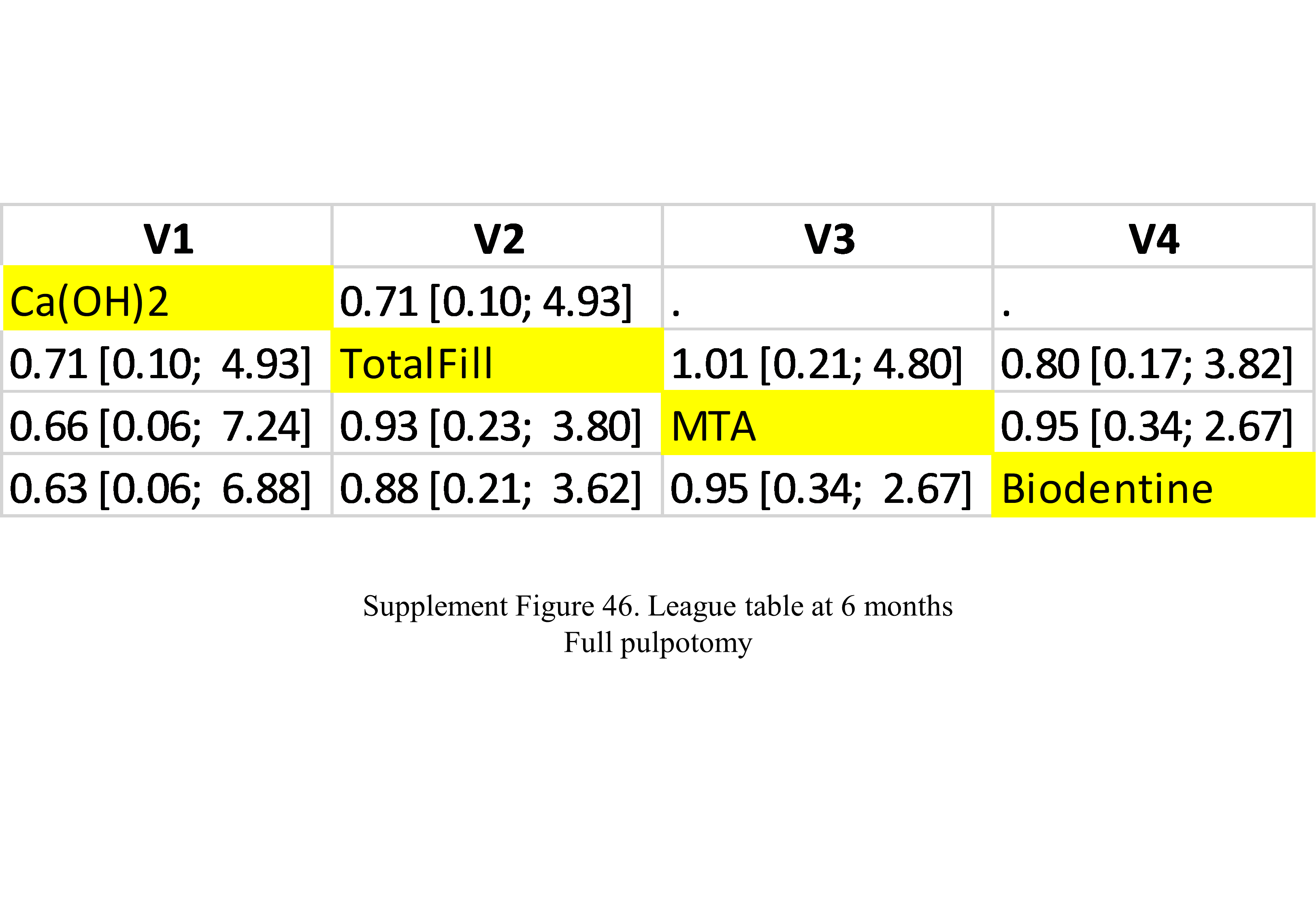

Supplement: Supplementary file 46 — Supplementary Figure 46. [file 41598_2024_69367_MOESM46_ESM.tif]

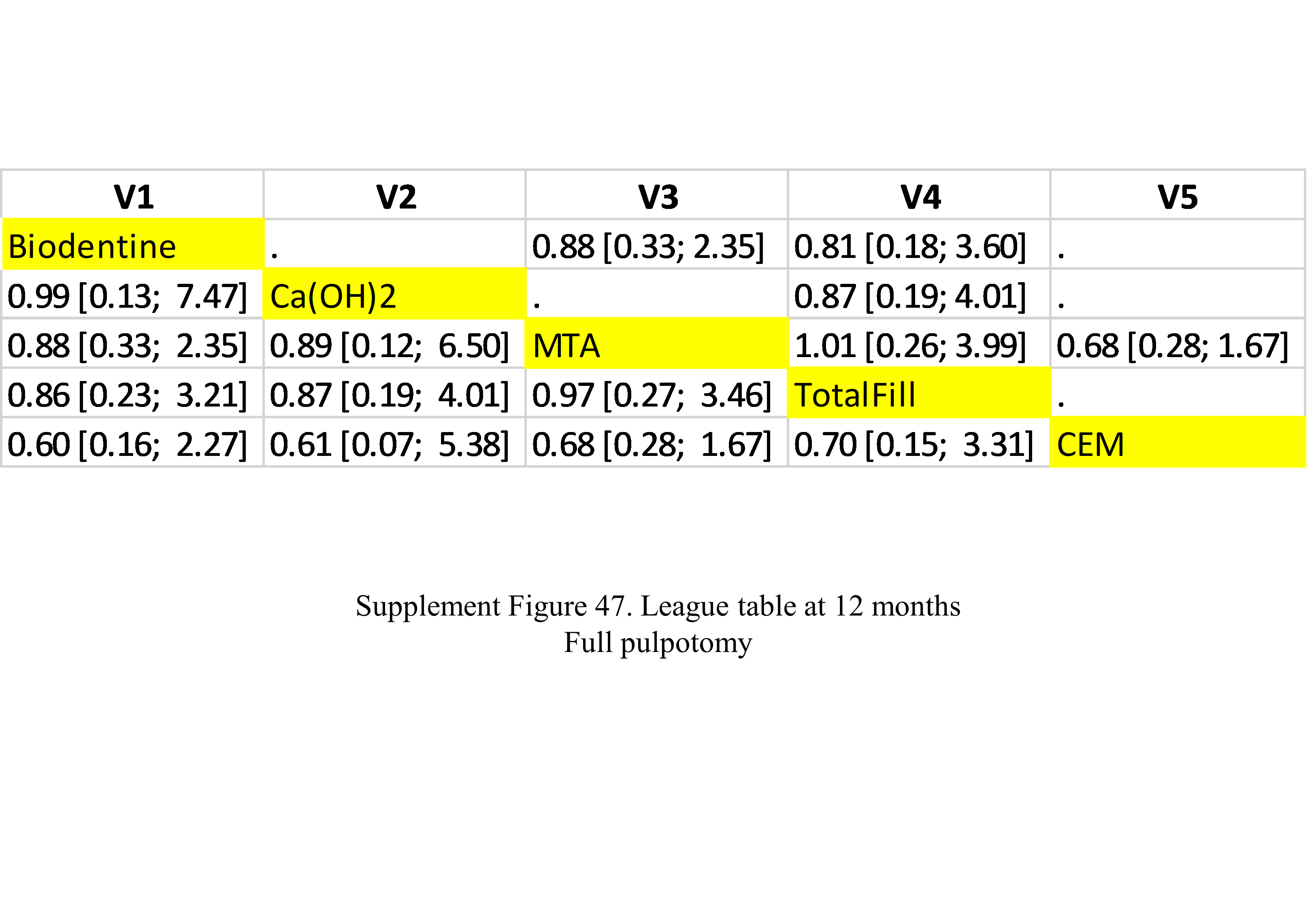

Supplement: Supplementary file 47 — Supplementary Figure 47. [file 41598_2024_69367_MOESM47_ESM.tif]

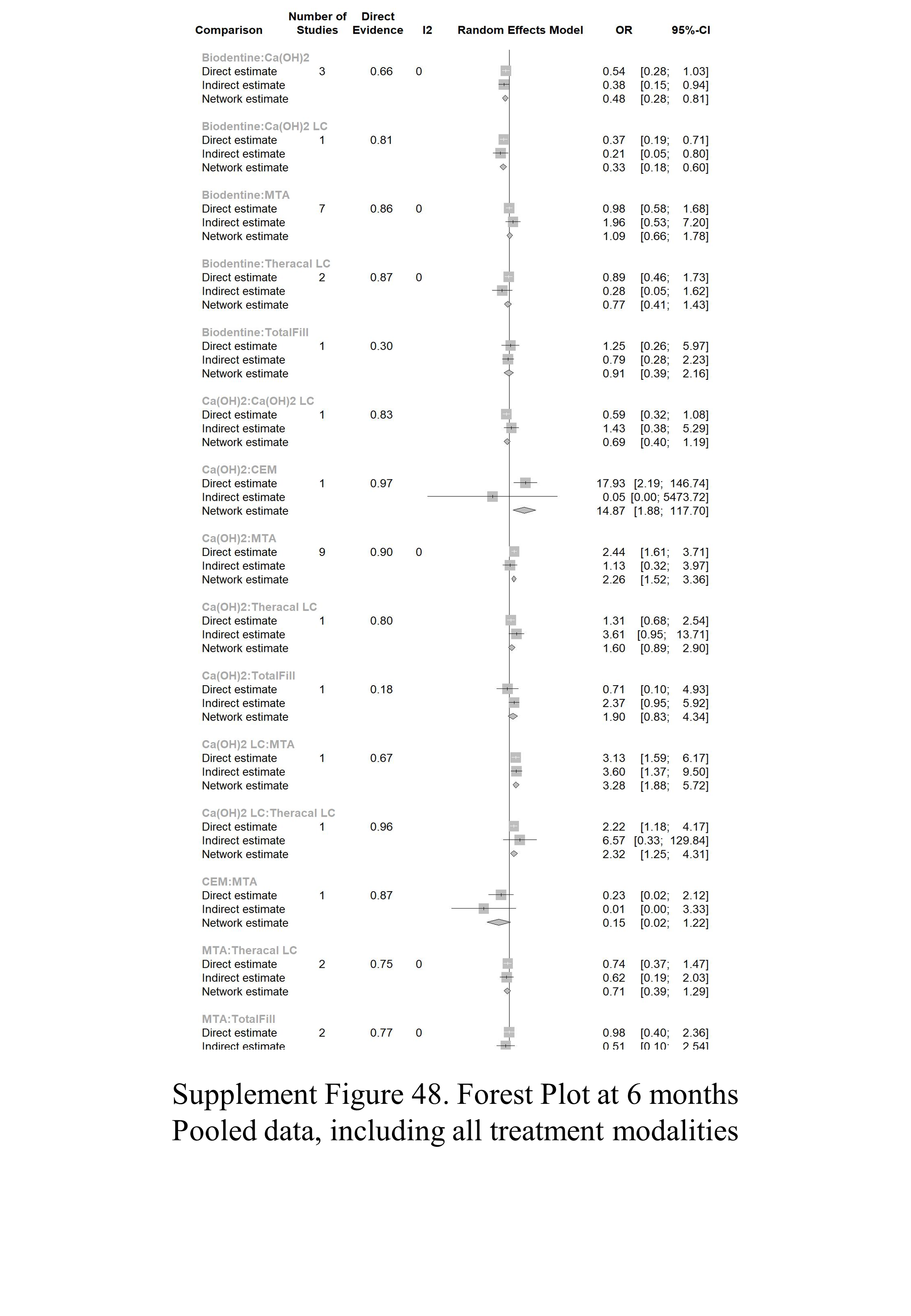

Supplement: Supplementary file 48 — Supplementary Figure 48. [file 41598_2024_69367_MOESM48_ESM.tif]

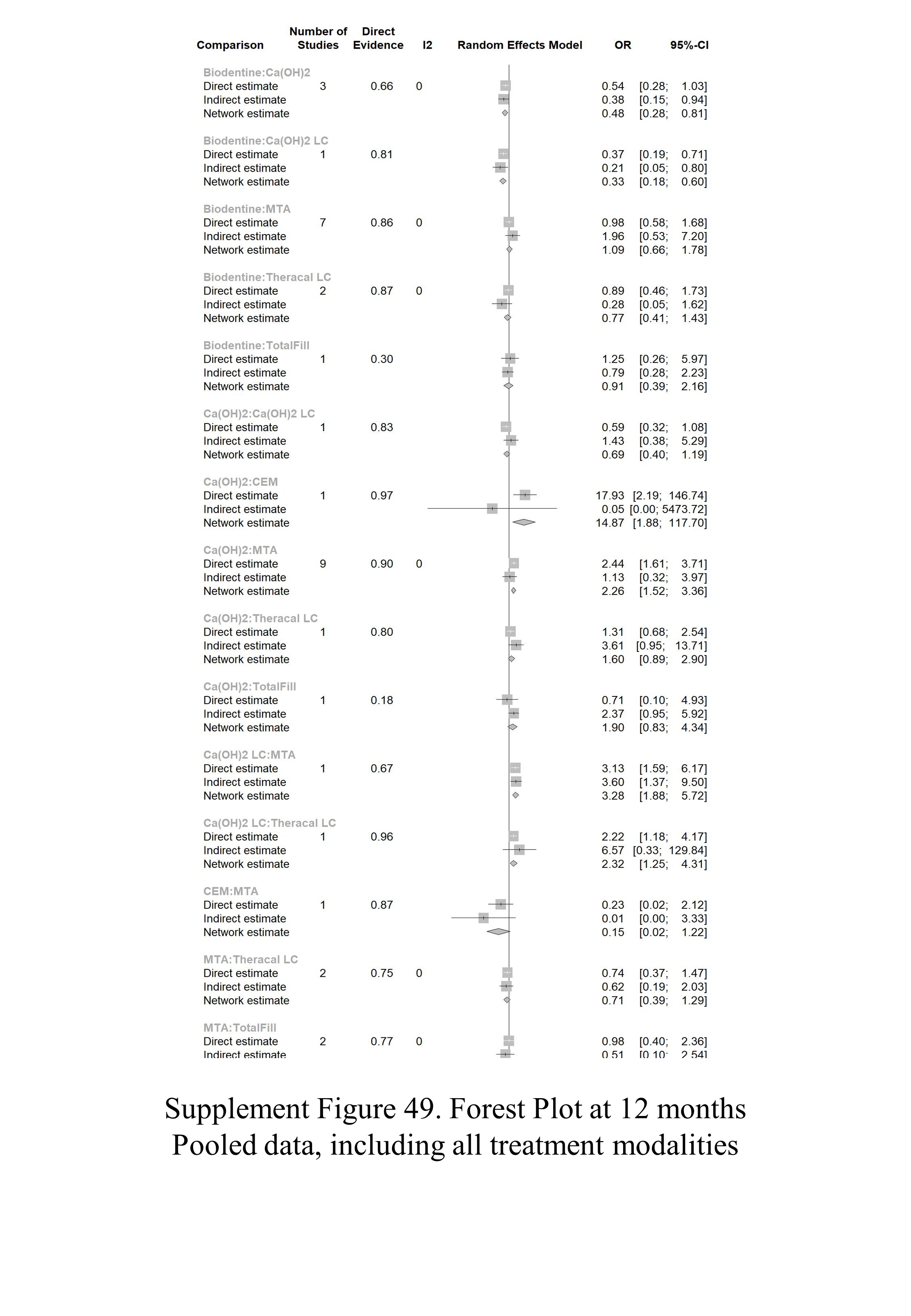

Supplement: Supplementary file 49 — Supplementary Figure 49. [file 41598_2024_69367_MOESM49_ESM.tif]

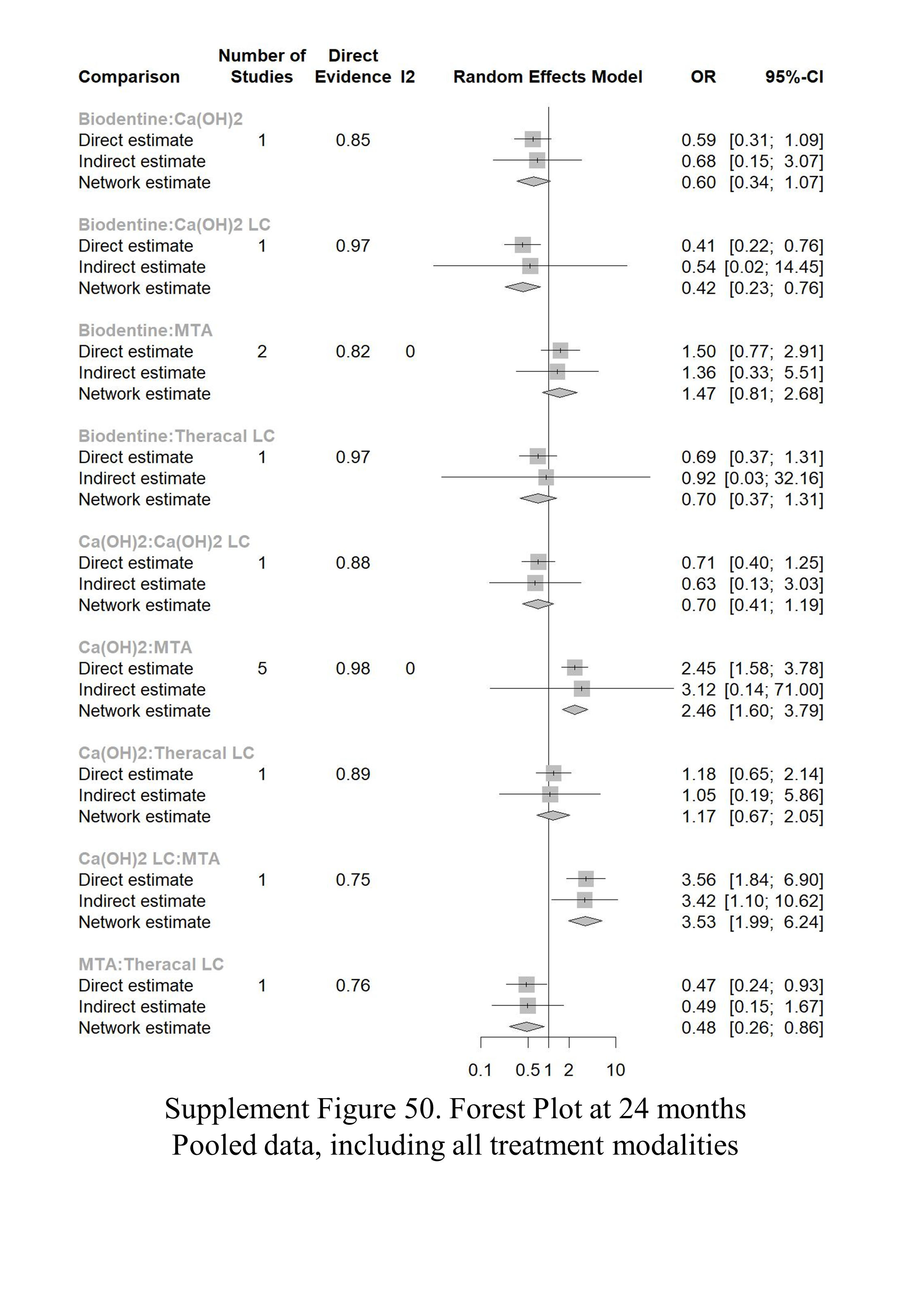

Supplement: Supplementary file 50 — Supplementary Figure 50. [file 41598_2024_69367_MOESM50_ESM.tif]

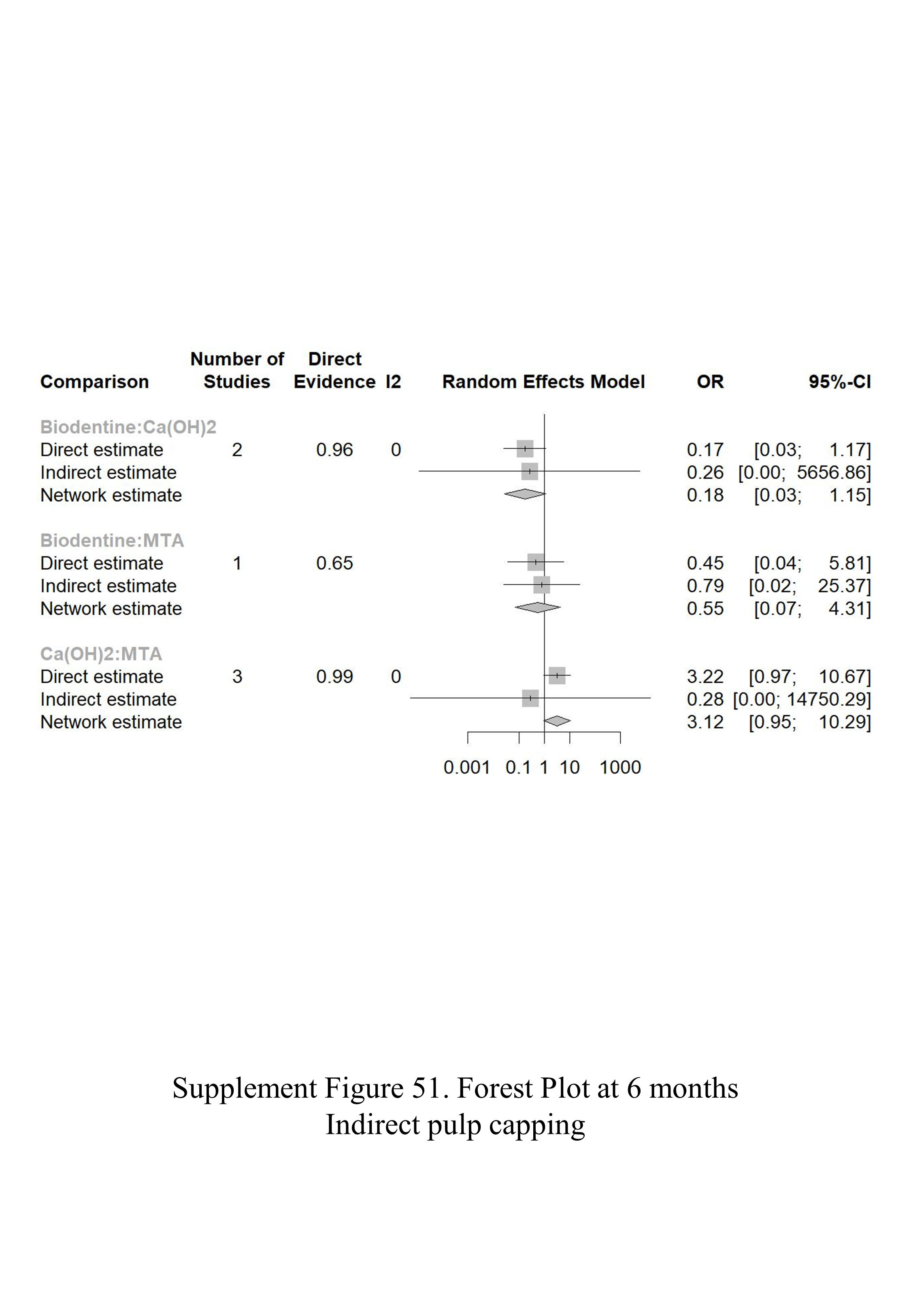

Supplement: Supplementary file 51 — Supplementary Figure 51. [file 41598_2024_69367_MOESM51_ESM.tif]

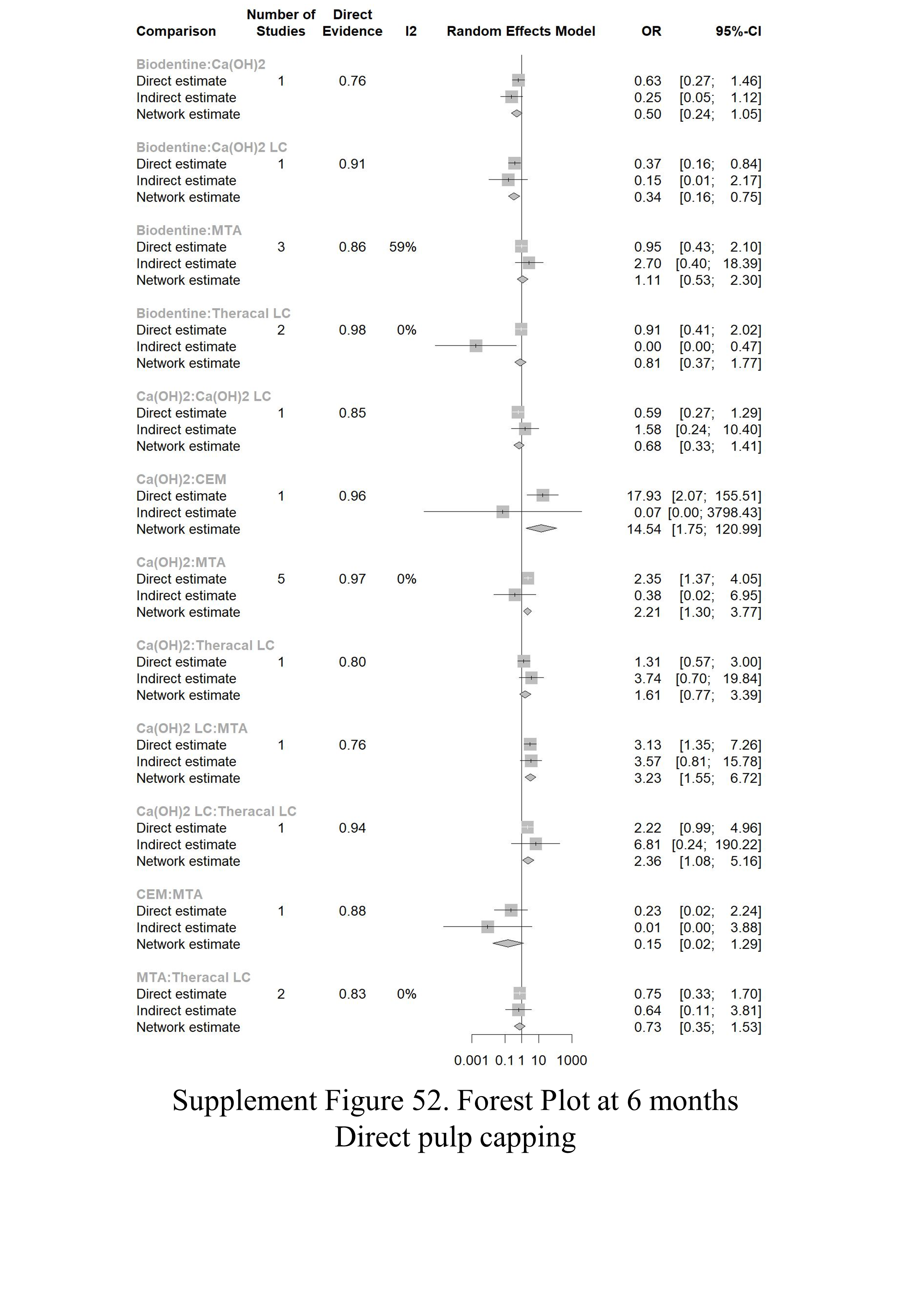

Supplement: Supplementary file 52 — Supplementary Figure 52. [file 41598_2024_69367_MOESM52_ESM.tif]

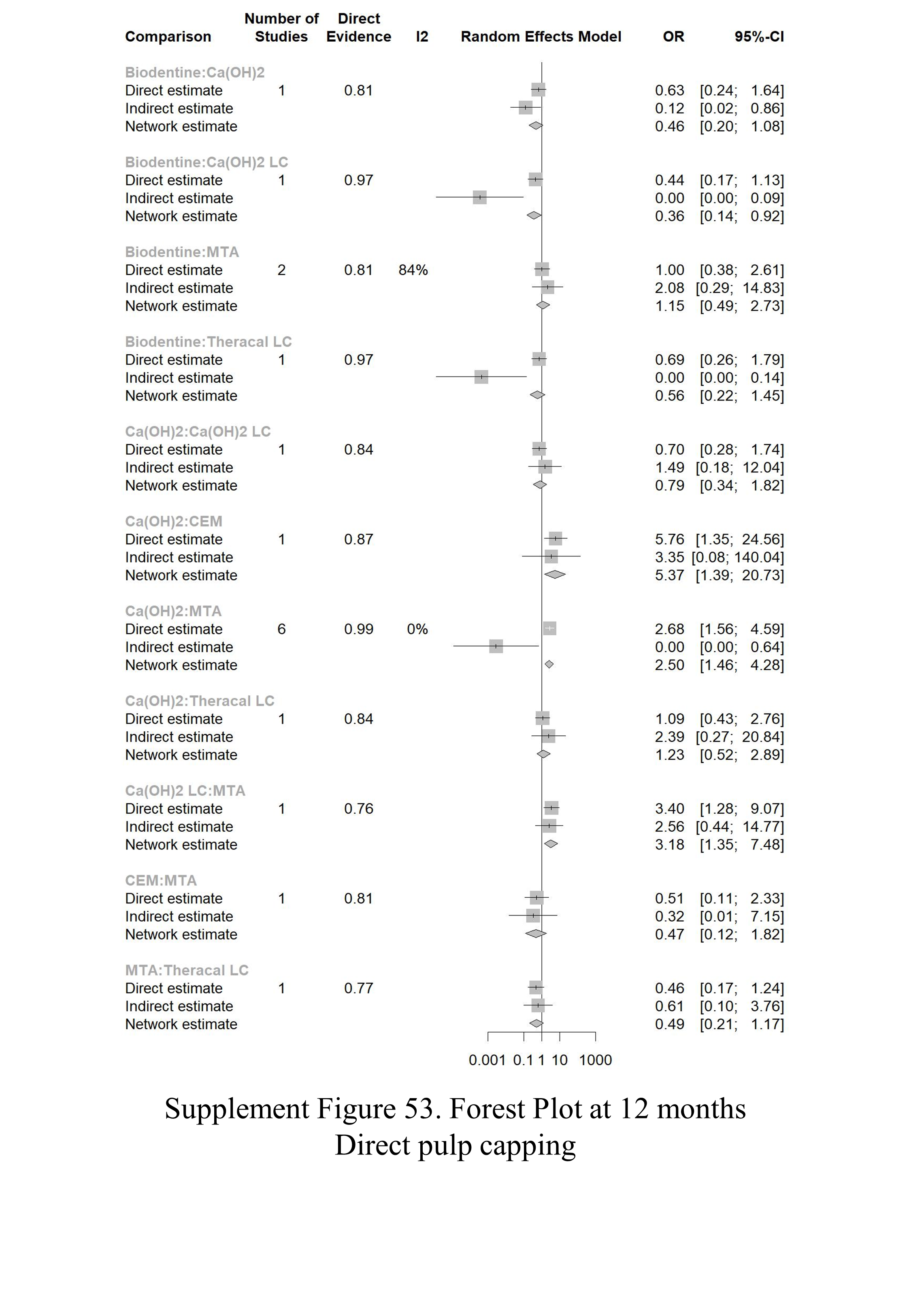

Supplement: Supplementary file 53 — Supplementary Figure 53. [file 41598_2024_69367_MOESM53_ESM.tif]

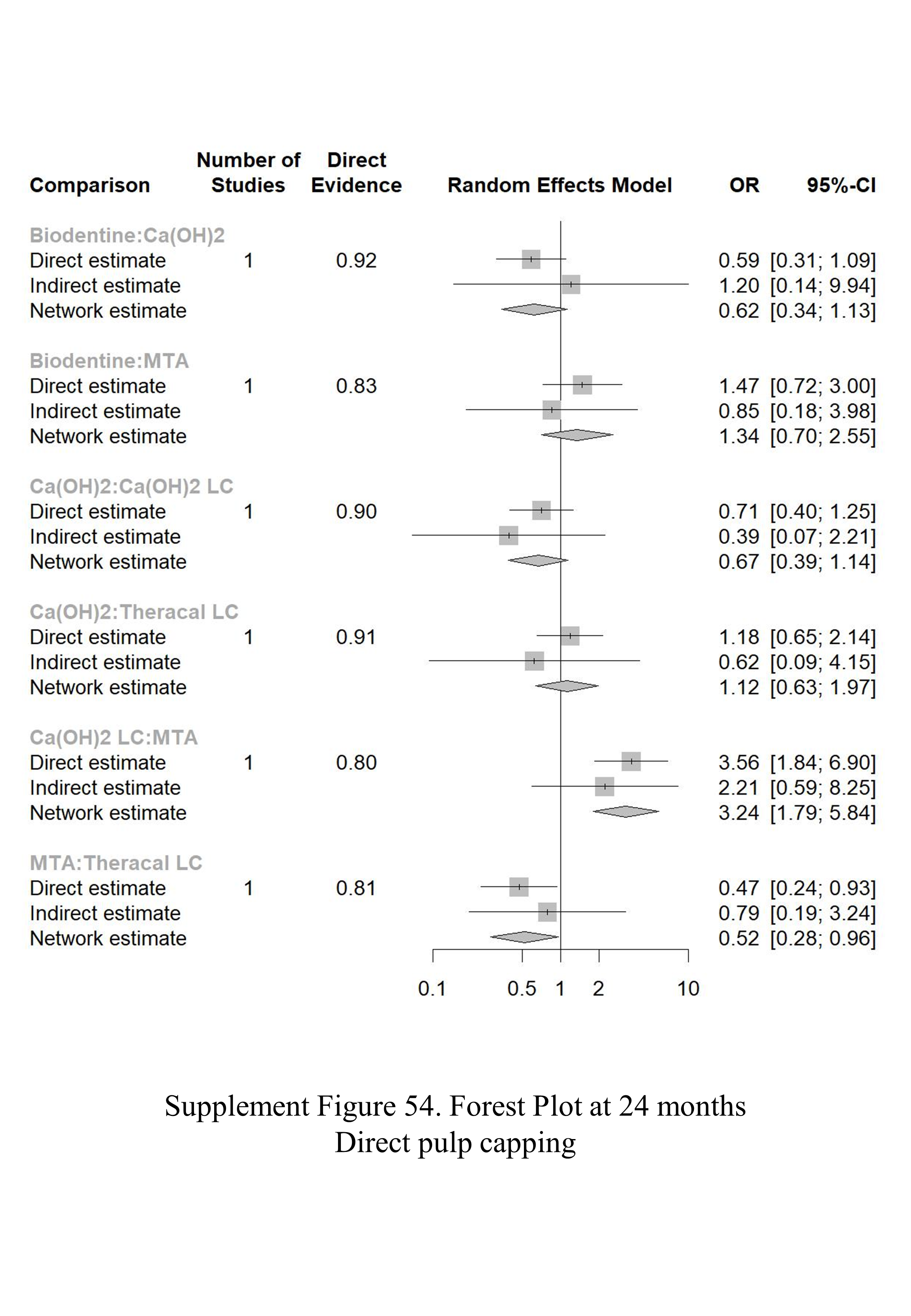

Supplement: Supplementary file 54 — Supplementary Figure 54. [file 41598_2024_69367_MOESM54_ESM.tif]

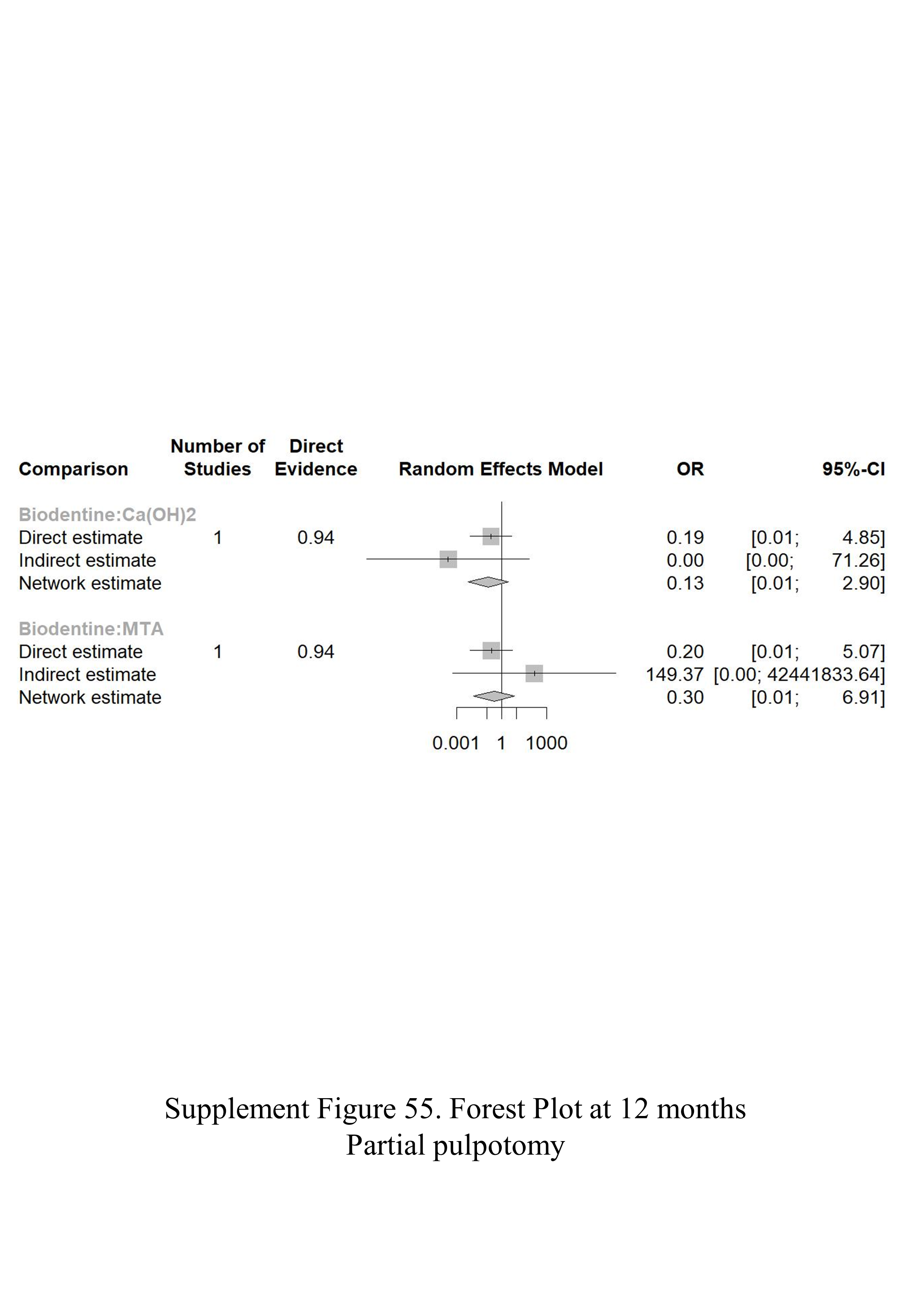

Supplement: Supplementary file 55 — Supplementary Figure 55. [file 41598_2024_69367_MOESM55_ESM.tif]

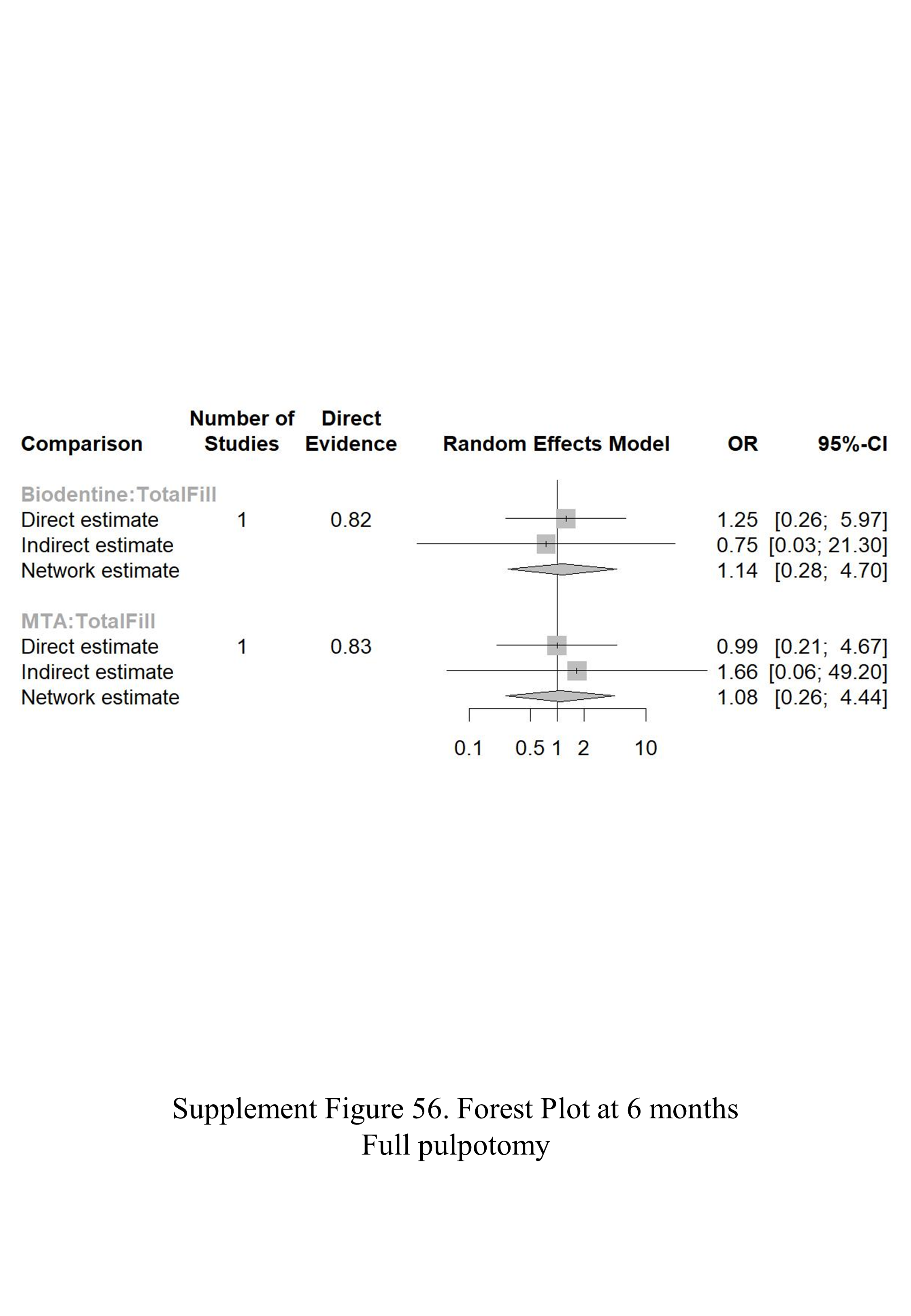

Supplement: Supplementary file 56 — Supplementary Figure 56. [file 41598_2024_69367_MOESM56_ESM.tif]

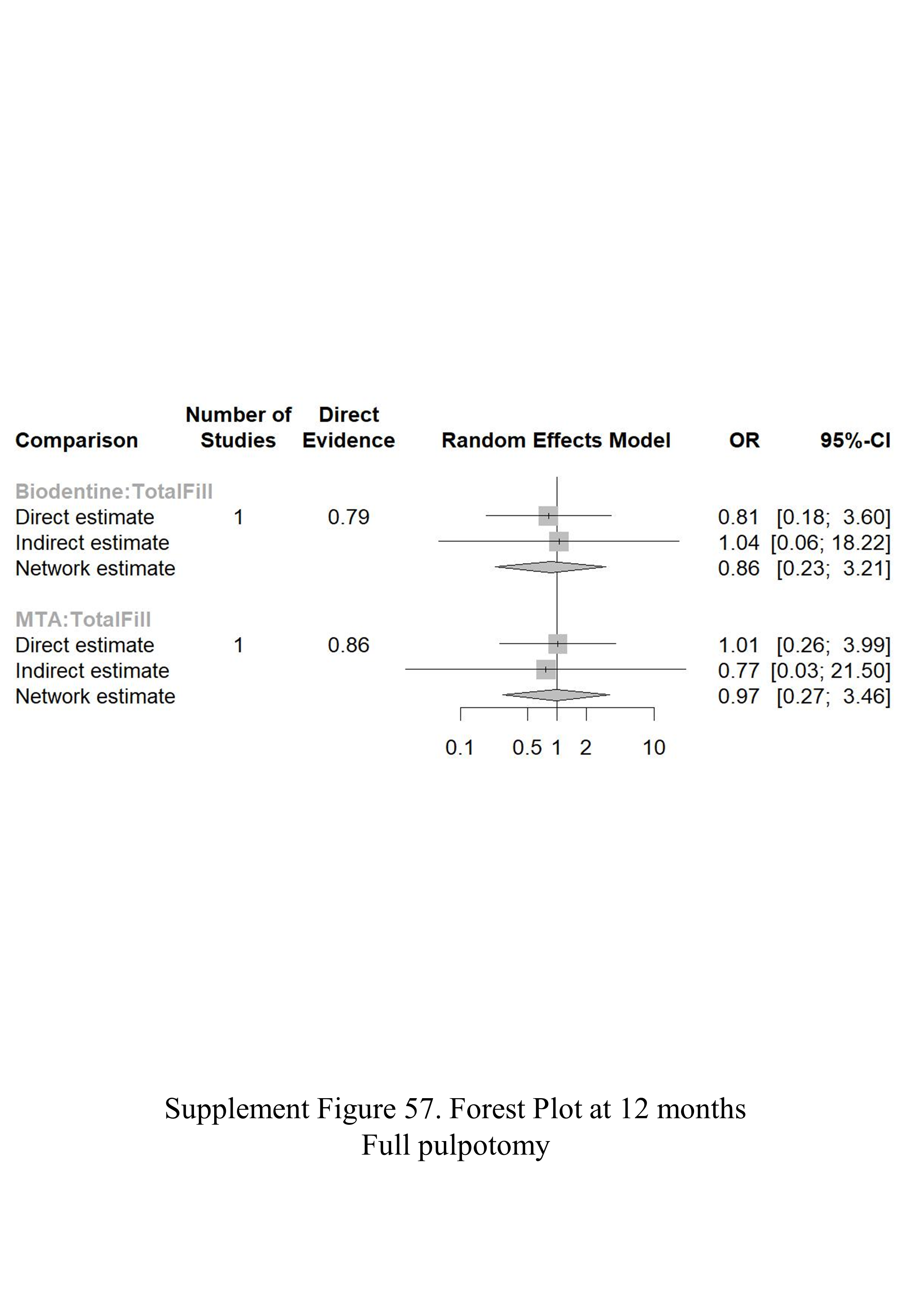

Supplement: Supplementary file 57 — Supplementary Figure 57. [file 41598_2024_69367_MOESM57_ESM.tif]
