# Supplementary material for: Comparison of bioactive material failure rates in vital pulp treatment of permanent matured teeth – a systematic review and network meta-analysis
Source: Sci Rep. 2024 Aug 8;14:18421. doi: 10.1038/s41598-024-69367-7 (PMC11310317; doi:10.1038/s41598-024-69367-7)
Supplement: Supplementary file 58 — Supplementary Legends. [file 41598_2024_69367_MOESM58_ESM.docx]

**Supporting information (after reference)**

**Supplementary Figure 1** Risk of bias. The analysis was conducted using RoB2 Tools. The figure displays five distinct domains.

**Supplementary Figure 2** Funnel Plot 6 at months - pooled data, including all treatment modalities. The funnel plots were used to investigate the small study effect. Each symbol represents a study. The y-axis represents study precision, and the x-axis shows the study's result.

**Supplementary Figure 3** Funnel Plot 12 at months - pooled data, including all treatment modalities. The funnel plots were used to investigate the small study effect. Each symbol represents a study. The y-axis represents study precision, and the x-axis shows the study's result.

**Supplementary Figure 4** Funnel Plot 24 at months - pooled data, including all treatment modalities. The funnel plots were used to investigate the small study effect. Each symbol represents a study. The y-axis represents study precision, and the x-axis shows the study's result.

**Supplementary Figure 5** Funnel Plot at 6 months - indirect pulp capping. The funnel plots were used to investigate the small study effect. Each symbol represents a study. The y-axis represents study precision, and the x-axis shows the study's result.

**Supplementary Figure 6** Funnel Plot at 6 months - direct pulp capping. The funnel plots were used to investigate the small study effect. Each symbol represents a study. The y-axis represents study precision, and the x-axis shows the study's result.

**Supplementary Figure 7** Funnel Plot at 12 months - direct pulp capping. The funnel plots were used to investigate the small study effect. Each symbol represents a study. The y-axis represents study precision, and the x-axis shows the study's result.

**Supplementary Figure 8** Funnel Plot at 24 months - direct pulp capping. The funnel plots were used to investigate the small study effect. Each symbol represents a study. The y-axis represents study precision, and the x-axis shows the study's result.

**Supplementary Figure 9** Funnel Plot at 12 months – partial pulpotomy. The funnel plots were used to investigate the small study effect. Each symbol represents a study. The y-axis represents study precision, and the x-axis shows the study's result.

**Supplementary Figure 10** Funnel Plot at 6 months – full pulpotomy. The funnel plots were used to investigate the small study effect. Each symbol represents a study. The y-axis represents study precision, and the x-axis shows the study's result.

**Supplementary Figure 11** Funnel Plot at 12 months – full pulpotomy. The funnel plots were used to investigate the small study effect. Each symbol represents a study. The y-axis represents study precision, and the x-axis shows the study's result.

**Supplementary Figure 12** Certainty of evidence (CINeMA) 6 months results - pooled data, including all treatment modalities. The analysis included direct and indirect comparisons across six domains. The figure displays confidence ratings and reasons for downgrading.

**Supplementary Figure 13** Certainty of evidence (CINeMA) 12 months results - pooled data, including all treatment modalities. The analysis included direct and indirect comparisons across six domains. The figure displays confidence ratings and reasons for downgrading.

**Supplementary Figure 14** Certainty of evidence (CINeMA) 24 months results - pooled data, including all treatment modalities. The analysis included direct and indirect comparisons across six domains. The figure displays confidence ratings and reasons for downgrading.

**Supplementary Figure 15** Certainty of evidence (CINeMA) 6 months results - indirect pulp capping. The analysis included direct and indirect comparisons across six domains. The figure displays confidence ratings and reasons for downgrading.

**Supplementary Figure 16** Certainty of evidence (CINeMA) 6 months results - direct pulp capping. The analysis included direct and indirect comparisons across six domains. The figure displays confidence ratings and reasons for downgrading.

**Supplementary Figure 17** Certainty of evidence (CINeMA) 12 months results - direct pulp capping. The analysis included direct and indirect comparisons across six domains. The figure displays confidence ratings and reasons for downgrading.

**Supplementary Figure 18** Certainty of evidence (CINeMA) 24 months results - direct pulp capping. The analysis included direct and indirect comparisons across six domains. The figure displays confidence ratings and reasons for downgrading.

**Supplementary Figure 19** Certainty of evidence (CINeMA) 12 months results – partial pulpotomy. The analysis included direct and indirect comparisons across six domains. The figure displays confidence ratings and reasons for downgrading.

**Supplementary Figure 20** Certainty of evidence (CINeMA) 6 months results – full pulpotomy. The analysis included direct and indirect comparisons across six domains. The figure displays confidence ratings and reasons for downgrading.

**Supplementary Figure 21** Certainty of evidence (CINeMA) 12 months results – full pulpotomy. The analysis included direct and indirect comparisons across six domains. The figure displays confidence ratings and reasons for downgrading.

**Supplementary Figure 22** Evidence Plot at 6 months - pooled data, including all treatment modalities. The evidence plot displays the percentage of direct and indirect evidence used for each comparison, along with two additional metrics: minimal parallelism and mean path length. Minimal parallelism refers to the minimum number of independent paths that contribute to estimating an effect on an aggregated level. A mean path length above two warrants caution when interpreting comparison estimates.

**Supplementary Figure 23** Evidence Plot at 12 months - pooled data, including all treatment modalities. The evidence plot displays the percentage of direct and indirect evidence used for each comparison, along with two additional metrics: minimal parallelism and mean path length. Minimal parallelism refers to the minimum number of independent paths that contribute to estimating an effect on an aggregated level. A mean path length above two warrants caution when interpreting comparison estimates.

**Supplementary Figure 24** Evidence Plot at 24 months - pooled data, including all treatment modalities. The evidence plot displays the percentage of direct and indirect evidence used for each comparison, along with two additional metrics: minimal parallelism and mean path length. Minimal parallelism refers to the minimum number of independent paths that contribute to estimating an effect on an aggregated level. A mean path length above two warrants caution when interpreting comparison estimates.

**Supplementary Figure 25** Evidence Plot at 6 months – indirect pulp capping. The evidence plot displays the percentage of direct and indirect evidence used for each comparison, along with two additional metrics: minimal parallelism and mean path length. Minimal parallelism refers to the minimum number of independent paths that contribute to estimating an effect on an aggregated level. A mean path length above two warrants caution when interpreting comparison estimates.

**Supplementary Figure 26** Evidence Plot at 6 months – direct pulp capping. The evidence plot displays the percentage of direct and indirect evidence used for each comparison, along with two additional metrics: minimal parallelism and mean path length. Minimal parallelism refers to the minimum number of independent paths that contribute to estimating an effect on an aggregated level. A mean path length above two warrants caution when interpreting comparison estimates.

**Supplementary Figure 27** Evidence Plot at 12 months – direct pulp capping. The evidence plot displays the percentage of direct and indirect evidence used for each comparison, along with two additional metrics: minimal parallelism and mean path length. Minimal parallelism refers to the minimum number of independent paths that contribute to estimating an effect on an aggregated level. A mean path length above two warrants caution when interpreting comparison estimates.

**Supplementary Figure 28** Evidence Plot at 24 months – direct pulp capping. The evidence plot displays the percentage of direct and indirect evidence used for each comparison, along with two additional metrics: minimal parallelism and mean path length. Minimal parallelism refers to the minimum number of independent paths that contribute to estimating an effect on an aggregated level. A mean path length above two warrants caution when interpreting comparison estimates.

**Supplementary Figure 29** Evidence Plot at 12 months – partial pulpotomy. The evidence plot displays the percentage of direct and indirect evidence used for each comparison, along with two additional metrics: minimal parallelism and mean path length. Minimal parallelism refers to the minimum number of independent paths that contribute to estimating an effect on an aggregated level. A mean path length above two warrants caution when interpreting comparison estimates.

**Supplementary Figure 30** Evidence Plot at 6 months – full pulpotomy. The evidence plot displays the percentage of direct and indirect evidence used for each comparison, along with two additional metrics: minimal parallelism and mean path length. Minimal parallelism refers to the minimum number of independent paths that contribute to estimating an effect on an aggregated level. A mean path length above two warrants caution when interpreting comparison estimates.

**Supplementary Figure 31** Evidence Plot at 12 months – full pulpotomy. The evidence plot displays the percentage of direct and indirect evidence used for each comparison, along with two additional metrics: minimal parallelism and mean path length. Minimal parallelism refers to the minimum number of independent paths that contribute to estimating an effect on an aggregated level. A mean path length above two warrants caution when interpreting comparison estimates.

**Supplementary Figure 32** Net heat Plot at 6 months - pooled data, including all treatment modalities. Net-heat plots display direct estimate contributions to network estimates, and inconsistency changes when removing one comparison. Grey square size displays direct evidence contribution (column) to network evidence (row). Colors show a change in inconsistency between direct and indirect evidence (row). Cold colors mean increased inconsistency and warm colors mean decreased.

**Supplementary Figure 33** Net heat Plot at 12 months - pooled data, including all treatment modalities. Net-heat plots display direct estimate contributions to network estimates, and inconsistency changes when removing one comparison. Grey square size displays direct evidence contribution (column) to network evidence (row). Colors show a change in inconsistency between direct and indirect evidence (row). Cold colors mean increased inconsistency and warm colors mean decreased.

**Supplementary Figure 34** Net heat Plot at 24 months - pooled data, including all treatment modalities. Net-heat plots display direct estimate contributions to network estimates, and inconsistency changes when removing one comparison. Grey square size displays direct evidence contribution (column) to network evidence (row). Colors show a change in inconsistency between direct and indirect evidence (row). Cold colors mean increased inconsistency and warm colors mean decreased.

**Supplementary Figure 35** Net heat Plot at 6 months – indirect pulp capping. Net-heat plots display direct estimate contributions to network estimates, and inconsistency changes when removing one comparison. Grey square size displays direct evidence contribution (column) to network evidence (row). Colors show a change in inconsistency between direct and indirect evidence (row). Cold colors mean increased inconsistency and warm colors mean decreased.

**Supplementary Figure 36** Net heat Plot at 6 months – direct pulp capping. Net-heat plots display direct estimate contributions to network estimates, and inconsistency changes when removing one comparison. Grey square size displays direct evidence contribution (column) to network evidence (row). Colors show a change in inconsistency between direct and indirect evidence (row). Cold colors mean increased inconsistency and warm colors mean decreased.

**Supplementary Figure 37** Net heat Plot at 12 months – direct pulp capping. Net-heat plots display direct estimate contributions to network estimates, and inconsistency changes when removing one comparison. Grey square size displays direct evidence contribution (column) to network evidence (row). Colors show a change in inconsistency between direct and indirect evidence (row). Cold colors mean increased inconsistency and warm colors mean decreased.

**Supplementary Figure 38** League table at 6 months - pooled data, including all treatment modalities. A league table in network meta-analysis displays all pairwise comparisons, including treatment estimates and confidence intervals. The numbers below the material names are the combined results of direct and indirect comparisons and above are the direct comparisons.

**Supplementary Figure 39** League table at 12 months - pooled data, including all treatment modalities. A league table in network meta-analysis displays all pairwise comparisons, including treatment estimates and confidence intervals. The numbers below the material names are the combined results of direct and indirect comparisons and above are the direct comparisons.

**Supplementary Figure 40** League table at 24 months - pooled data, including all treatment modalities. A league table in network meta-analysis displays all pairwise comparisons, including treatment estimates and confidence intervals. The numbers below the material names are the combined results of direct and indirect comparisons and above are the direct comparisons.

**Supplementary Figure 41** League table at 6 months – indirect pulp capping. A league table in network meta-analysis displays all pairwise comparisons, including treatment estimates and confidence intervals. The numbers below the material names are the combined results of direct and indirect comparisons and above are the direct comparisons.

**Supplementary Figure 42** League table at 6 months – direct pulp capping. A league table in network meta-analysis displays all pairwise comparisons, including treatment estimates and confidence intervals. The numbers below the material names are the combined results of direct and indirect comparisons and above are the direct comparisons.

**Supplementary Figure 43** League table at 12 months – direct pulp capping. A league table in network meta-analysis displays all pairwise comparisons, including treatment estimates and confidence intervals. The numbers below the material names are the combined results of direct and indirect comparisons and above are the direct comparisons.

**Supplementary Figure 44** League table at 24 months – direct pulp capping. A league table in network meta-analysis displays all pairwise comparisons, including treatment estimates and confidence intervals. The numbers below the material names are the combined results of direct and indirect comparisons and above are the direct comparisons.

**Supplementary Figure 45** League table at 12 months – partial pulpotomy. A league table in network meta-analysis displays all pairwise comparisons, including treatment estimates and confidence intervals. The numbers below the material names are the combined results of direct and indirect comparisons and above are the direct comparisons.

**Supplementary Figure 46** League table at 6 months – full pulpotomy. A league table in network meta-analysis displays all pairwise comparisons, including treatment estimates and confidence intervals. The numbers below the material names are the combined results of direct and indirect comparisons and above are the direct comparisons.

**Supplementary Figure 47** League table at 12 months – full pulpotomy. A league table in network meta-analysis displays all pairwise comparisons, including treatment estimates and confidence intervals. The numbers below the material names are the combined results of direct and indirect comparisons and above are the direct comparisons.

**Supplementary Figure 48** Forest Plot at 6 months - pooled data, including all treatment modalities. The forest plot represents the compared materials. The grey squares represent the odds ratio (OR), and the length of the black lines shows 95% (CI) confidence intervals in direct and indirect comparisons. The grey diamonds represent the odds ratio (OR), and the length of the diamond shows 95% (CI) confidence intervals in network estimates (combined direct and indirect comparisons). The squares and diamonds on the left side of the zero effect represent that the first material is favorable, and on the right side, the second is favorable.

**Supplementary Figure 49** Forest Plot at 12 months - pooled data, including all treatment modalities. The forest plot represents the compared materials. The grey squares represent the odds ratio (OR), and the length of the black lines shows 95% (CI) confidence intervals in direct and indirect comparisons. The grey diamonds represent the odds ratio (OR), and the length of the diamond shows 95% (CI) confidence intervals in network estimates (combined direct and indirect comparisons). The squares and diamonds on the left side of the zero effect represent that the first material is favorable, and on the right side, the second is favorable.

**Supplementary Figure 50** Forest Plot at 24 months - pooled data, including all treatment modalities. The forest plot represents the compared materials. The grey squares represent the odds ratio (OR), and the length of the black lines shows 95% (CI) confidence intervals in direct and indirect comparisons. The grey diamonds represent the odds ratio (OR), and the length of the diamond shows 95% (CI) confidence intervals in network estimates (combined direct and indirect comparisons). The squares and diamonds on the left side of the zero effect represent that the first material is favorable, and on the right side, the second is favorable.

**Supplementary Figure 51** Forest Plot at 6 months – indirect pulp capping. The forest plot represents the compared materials. The grey squares represent the odds ratio (OR), and the length of the black lines shows 95% (CI) confidence intervals in direct and indirect comparisons. The grey diamonds represent the odds ratio (OR), and the length of the diamond shows 95% (CI) confidence intervals in network estimates (combined direct and indirect comparisons). The squares and diamonds on the left side of the zero effect represent that the first material is favorable, and on the right side, the second is favorable.

**Supplementary Figure 52** Forest Plot at 6 months – direct pulp capping. The forest plot represents the compared materials. The grey squares represent the odds ratio (OR), and the length of the black lines shows 95% (CI) confidence intervals in direct and indirect comparisons. The grey diamonds represent the odds ratio (OR), and the length of the diamond shows 95% (CI) confidence intervals in network estimates (combined direct and indirect comparisons). The squares and diamonds on the left side of the zero effect represent that the first material is favorable, and on the right side, the second is favorable.

**Supplementary Figure 53** Forest Plot at 12 months – direct pulp capping. The forest plot represents the compared materials. The grey squares represent the odds ratio (OR), and the length of the black lines shows 95% (CI) confidence intervals in direct and indirect comparisons. The grey diamonds represent the odds ratio (OR), and the length of the diamond shows 95% (CI) confidence intervals in network estimates (combined direct and indirect comparisons). The squares and diamonds on the left side of the zero effect represent that the first material is favorable, and on the right side, the second is favorable.

**Supplementary Figure 54** Forest Plot at 24 months – direct pulp capping. The forest plot represents the compared materials. The grey squares represent the odds ratio (OR), and the length of the black lines shows 95% (CI) confidence intervals in direct and indirect comparisons. The grey diamonds represent the odds ratio (OR), and the length of the diamond shows 95% (CI) confidence intervals in network estimates (combined direct and indirect comparisons). The squares and diamonds on the left side of the zero effect represent that the first material is favorable, and on the right side, the second is favorable.

**Supplementary Figure 55** Forest Plot at 12 months – partial pulpotomy. The forest plot represents the compared materials. The grey squares represent the odds ratio (OR), and the length of the black lines shows 95% (CI) confidence intervals in direct and indirect comparisons. The grey diamonds represent the odds ratio (OR), and the length of the diamond shows 95% (CI) confidence intervals in network estimates (combined direct and indirect comparisons). The squares and diamonds on the left side of the zero effect represent that the first material is favorable, and on the right side, the second is favorable.

**Supplementary Figure 56** Forest Plot at 6 months – full pulpotomy. The forest plot represents the compared materials. The grey squares represent the odds ratio (OR), and the length of the black lines shows 95% (CI) confidence intervals in direct and indirect comparisons. The grey diamonds represent the odds ratio (OR), and the length of the diamond shows 95% (CI) confidence intervals in network estimates (combined direct and indirect comparisons). The squares and diamonds on the left side of the zero effect represent that the first material is favorable, and on the right side, the second is favorable.

**Supplementary Figure 57** Forest Plot at 12 months – full pulpotomy. The forest plot represents the compared materials. The grey squares represent the odds ratio (OR), and the length of the black lines shows 95% (CI) confidence intervals in direct and indirect comparisons. The grey diamonds represent the odds ratio (OR), and the length of the diamond shows 95% (CI) confidence intervals in network estimates (combined direct and indirect comparisons). The squares and diamonds on the left side of the zero effect represent that the first material is favorable, and on the right side, the second is favorable.
