# Supplementary material for: Comparison of bioactive material failure rates in vital pulp treatment of permanent matured teeth – a systematic review and network meta-analysis
Source: Sci Rep. 2024 Aug 8;14:18421. doi: 10.1038/s41598-024-69367-7 (PMC11310317; doi:10.1038/s41598-024-69367-7)
Supplement: Supplementary file 3 [file 41598_2024_69367_MOESM59_ESM.docx]

Supplementary table 1. Excluded full text articles

Title	Author	Date	DOI	ISSN	Clinical Trials	Reason for exlusion
Randomized control trial comparing calcium hydroxide and mineral trioxide aggregate for partial pulpotomies in cariously exposed pulps of permanent molars	Chailertvanitkul et al.	2014	10.1111/iej.12225			The outcome did not match the selection criteria
Capping carious exposures in adults: a randomized controlled trial investigating mineral trioxide aggregate versus calcium hydroxide	Kundzina et al	2017	10.1111/iej.12719			The outcome did not match the selection criteria
Clinical evaluation of mineral trioxide aggregate and biodentine as direct pulp capping agents in carious teeth	Hegde et al.	2017	10.4103/0972-0707.212243			The outcome did not match the selection criteria
Partial pulpotomy with two bioactive cements in permanent teeth of 6- to 18-year-old patients with signs and symptoms indicative of irreversible pulpitis: a noninferiority randomized controlled trial	Uesrichai et al.	2019	10.1111/iej.13071			The outcome did not match the selection criteria
Postendodontic Pain after Pulpotomy or Root Canal Treatment in Mature Teeth with Carious Pulp Exposure: A Multicenter Randomized Controlled Trial	Eghbal et al.	2020	10.1155/2020/5853412			The outcome did not match the selection criteria
Tomographic evaluation of direct pulp capping using a novel injectable treated dentin matrix hydrogel: a 2-year randomized controlled clinical trial	Holiel et al.	2021	10.1007/s00784-021-03775-1			The outcome did not match the selection criteria
Calcium hydroxide vs mineral trioxide aggregates for partial pulpotomy of permanent molars with deep caries	Qudeimat et al.	2007	10.1007/BF03262577			Included a population of non-mature teeth
A randomized clinical trial on the use of medical Portland cement, MTA and calcium hydroxide in indirect pulp treatment	Petrou et al.	2014	10.1007/s00784-013-1107-z			Included a population of non-mature teeth
Comparative Analysis of 2 Calcium Silicate-based Cements (Biodentine and Mineral Trioxide Aggregate) as Direct Pulp-capping Agent in Young Permanent Molars: A Split Mouth Study	Katge and Patil	2017	10.1016/j.joen.2016.11.026			Included a population of non-mature teeth
Outcomes of Direct Pulp Capping by Using Either ProRoot Mineral Trioxide Aggregate or Biodentine in Permanent Teeth with Carious Pulp Exposure in 6- to 18-Year-Old Patients: A Randomized Controlled Trial	Parinyaprom et al.	2018	10.1016/j.joen.2017.10.012			Included a population of non-mature teeth
Comparative Evaluation of Indirect Pulp Therapy in Young Permanent Teeth using Biodentine and Theracal: A Randomized Clinical Trial	Rahman and Goswami	2021	10.17796/1053-4625-45.3.3			Included a population of non-mature teeth
Comparative evaluation of platelet-rich fibrin, mineral trioxide aggregate, and calcium hydroxide as pulpotomy agents in permanent molars with irreversible pulpitis: A randomized controlled trial	Kumar et al.	2016	10.4103/0976-237X.194107			Data were not available for calculation
A randomized controlled trial of various MTA materials for partial pulpotomy in permanent teeth	Kang et al.	2017	10.1016/j.jdent.2016.07.015			Data were not available for calculation
Direct Pulp Capping with Calcium Hydroxide, Mineral Trioxide Aggregate, and Biodentine in Permanent Young Teeth with Caries: A Randomized Clinical Trial	Brizuela et al.	2017	10.1016/j.joen.2017.06.031			Data were not available for calculation
Prognostic factors in direct pulp capping with mineral trioxide aggregate or calcium hydroxide: 2- to 6-year follow-up	Çalışkan and Güneri	2017	doi:10.1007/s00784-016-1798-z			Data were not available for calculation
A randomized controlled study of the use of ProRoot mineral trioxide aggregate and Endocem as direct pulp capping materials	Song et al.	2015	10.1016/j.joen.2014.09.005			The follow-up period was only three months
To Compare the Effectiveness of Biodentine versus Mineral Trioxide aggregate as Direct Pulp Capping agent in carious exposed permanent tooth	Anwar et al.	2022	10.53350/pjmhs22167172			The follow-up period was only three months
Clinical and histological analyzes of the response of the pulp after its direct capping with Calxyl, MTA and Biodentine	Eftimoska et al.	2015	N/A	ISSN: 0975-8585		Treated teeth were extracted
A Randomized Controlled Study of the Use of ProRoot Mineral Trioxide Aggregate and Endocem as Direct Pulp Capping Materials: 3-month versus 1-year Outcomes	Jang et al.	2015	10.1016/j.joen.2015.03.015			Traumatic cases were treated
Treatment Outcomes of 4 Vital Pulp Therapies in Mature Molars	Asgary et al.	2018	10.1016/j.joen.2017.12.010			No comparison of materials was made
Mineral Trioxide Aggregate Could Have a Better Success Rate Than Calcium Hydroxide for Partial Pulpotomy of Symptomatic Mature Permanent Molars	Bakhurji	2020	10.1016/j.jebdp.2019.101341			Review
Evaluation of bioceramic putty repairmen iRoot and mineral trioxide aggregate in mature permanent teeth pulpotomy	Qian et al.	2022	10.19723/j.issn.1671-167X.2022.01.018			No english full-text
Does carious exposure in adult patient really warrant direct capping?	Kundzina et al.	2015	N/A			Articles were not accessible/Conference abstract
Long-term outcomes of pulpotomy in permanent teeth with irreversible pulpitis: A multi-center randomized controlled trial	Asgary et al.	2017	N/A			Articles were not accessible
Comparative evaluation of the efficacy of theracal lc, mineral trioxide aggregate and biodentine as direct pulp capping materials in patients with pulpal exposure in posterior teeth-an interventional study	Mahapatra et al.	2019	doi:10.31838/ijpr/2019.11.02.204			Articles were not accessible/Published study protocol without results
Comparative evaluation of the efficacy of light cure calcium hydroxide and theracal LC as indirect pulp capping materials in patients with deep carious lesion-An interventional study	Mahapatra et al.	2020	N/A			Published study protocol without results
Clinical Comparison of Three Indirect Pulp Capping Restorative Protocols: A Randomized Controlled Prospective Study	Semprum-Clavier et al.	2024	10.2341/22-094-c			Articles were not accessible
Is Bioactive Glass an Effective Agent in Pulp-capping Treatments?: A Randomized Controlled Clinical Trial with One-year Follow-up	İnci and Korkut.	2022	10.5005/jp-journals-10024-3428			Included a population of primary teeth
Role Of Prf With Mta And Theracal After Pulpotomy In Relieving Pain And Maintaining The Vitality Of The Remaining Radicular Pulp Tissue In Permanent Posterior Teeth With Closed Root Apices: "Randomized Controlled Trial"	Mohammed et al.	2022	10.47750/pnr.2022.13.S05.396			The outcome did not match the selection criteria
Coronal discoloration induced by calcium silicate-based cements used in full pulpotomy in mature permanent molars: a randomized clinical trial	Taha et al.	2023	10.1007/s00784-022-04799-x			The outcome did not match the selection criteria
Comparison of the effect of iRoot BP Plus and MTA on direct pulp capping of carious pulp exposed mature permanent teeth	Cai et al.	2023	10.12016/j.issn.2096-1456.2023.06.006			No english full-text
[illegible] Procedures Using Ca $(OH)_2$ and Bioactive Tricalcium Silicate Paste vs. Laser-Assisted Procedures (Diode 980 nm, CO_2, and Er: YAG)	Nammour et al.	2023	10.3390/photonics10070834			Data were not available for calculation
X-ray evaluation of pulp calcification in adult permanent teeth after pulpotomy	Yong et al.	2023	10.19723/j.issn.1671-167X.2023.01.013			No english full-text
Effect Of Different Lining Materials in Stepwise Excavation		2012			https://clinicaltrials.gov/show/NCT01730287	Not finished registered study
Calcium Hydroxide, Mineral Trioxide Aggregate and Biodentine (Trademark) as Direct Pulp Capping of Permanent Teeth		2015			https://clinicaltrials.gov/show/NCT02492841	Not finished registered study
Comparison of efficacy of two pulp-capping materials for temperature stimulation positive deep caries permanent teeth		2016			https://trialsearch.who.int/Trial2.aspx?TrialID=ChiCTR-IOR-16007982	Not finished registered study
Partial pulpotomy in permanent teeth		2016			https://trialsearch.who.int/Trial2.aspx?TrialID=ISRCTN16363154	Not finished registered study
Direct Pulp Capping With MTA and Calcium Hydroxide		2017			https://clinicaltrials.gov/show/NCT03284697	Not finished registered study
Effect of Pulpotomy Using TheraCal Versus MTA on Survival Rate of Cariously-Exposed Vital Permanent Molars		2017			https://clinicaltrials.gov/show/NCT03119779	Not finished registered study
Similar Outcomes of Vital Pulp Therapy Using Mineral Trioxide Aggregate or Biodentine™		2017			https://clinicaltrials.gov/show/NCT03186690	Not finished registered study
Outcomes of Direct Pulp Capping Using Either ProRoot MTAÂ® or Biodentineâ?¢ in Permanent Teeth With Carious Pulp Exposure in 6-18-year-old Patients		2017			https://trialsearch.who.int/Trial2.aspx?TrialID=TCTR20170526003	Not finished registered study
Partial Pulpotomy Comparing Two Tricalcium Silicate Cement as Pulp Capping Material in Permanent Teeth with Cariously exposed Pulp in Adult Patients		2017			https://trialsearch.who.int/Trial2.aspx?TrialID=TCTR20171228003	Not finished registered study
Indirect Pulp Capping With Biodentine and TheraCal LC in Permanent Mature Molars		2018			https://clinicaltrials.gov/show/NCT03741816	Not finished registered study
Outcomes of Partial Pulpotomy Using Either ProRoot MTAÂ® or Biodentineâ?¢ in Permanent Teeth Diagnosed with Irreversible Pulpitis of 6-18 Years Old Patients		2018			https://trialsearch.who.int/Trial2.aspx?TrialID=TCTR20180612004	Not finished registered study
Pulpotomy in Mature Permanent Molars Using Biodentine Versus MTA		2019			https://clinicaltrials.gov/show/NCT03890835	Not finished registered study
Comparison of pulpotomy in teeth using MTA and biodentine		2020			https://trialsearch.who.int/Trial2.aspx?TrialID=CTRI/2020/03/023766	Not finished registered study
Comparison of Theracal LC, Mineral Trioxide Aggregate and Biodentine as tooth saviours		2020			https://trialsearch.who.int/Trial2.aspx?TrialID=CTRI/2020/01/023047	Not finished registered study
Evaluation of response to partial pulpotomy performed using different dental materials in human subjects		2020			https://trialsearch.who.int/Trial2.aspx?TrialID=CTRI/2020/11/029329	Not finished registered study
Outcome of partial pulpotomy and vital pulp therapy of teeth using biodentine compared to mineral trioxide aggregates (MTA)		2020			https://trialsearch.who.int/Trial2.aspx?TrialID=DRKS00022302	Not finished registered study
Comparing the Outcome of Pulpotomy Using Biodentine and Portland Cement in Mature Cases With Irreversible Pulpitis: randomized Clinical Trial		2020			https://clinicaltrials.gov/show/NCT04573374	Not finished registered study
MTA vs CEM Pulpotomy in Young Permanent Molars		2020			https://clinicaltrials.gov/show/NCT04243733	Not finished registered study
Outcome of Full Pulpotomy Using Calcium Silicate Based Materials		2020			https://clinicaltrials.gov/show/NCT04345263	Not finished registered study
Clinical study of vital pulp preservation treatment of carious teeth with pulpitis		2021			https://trialsearch.who.int/Trial2.aspx?TrialID=ChiCTR2100047588	Not finished registered study
Comparison of MTA and Biodentine pulpotomy in permanent teeth with fully formed root with irreversible pulpitis		2021			http://www.who.int/trialsearch/Trial2.aspx?TrialID=CTRI/2021/07/034720	Not finished registered study
Clinical trial to evaluate the success rate of indirect pulp capping procedure with four pulp capping materials in permanent teeth - a randomized clinical trial		2022			https://trialsearch.who.int/Trial2.aspx?TrialID=CTRI/2022/07/044347	Not finished registered study
