# Supplementary material for: Comparison of bioactive material failure rates in vital pulp treatment of permanent matured teeth – a systematic review and network meta-analysis
Source: Sci Rep. 2024 Aug 8;14:18421. doi: 10.1038/s41598-024-69367-7 (PMC11310317; doi:10.1038/s41598-024-69367-7)
Supplement: Supplementary file 4 [file 41598_2024_69367_MOESM60_ESM.docx]

Supplementary Table 2. D

Material	Chemical composition
MTA (ProRoot; Dentsply, Tulsa, OK) powder	Portland cement*
	Bismuth oxide
MTA (ProRoot; Dentsply, Tulsa, OK) liquid	Purified water
MTA (Angelus) powder	Portland cement*
	Iron oxide
	Calcium carbonate
	Magnesium oxide
	Calcium oxide
	Crystalline silica
MTA (Angelus) liquid	Distilled water
MTA (BioMTA+,Cerkamed, Stalowa Wola, Poland) Powder	Calcium oxide
	Hydroxyapatite
	Oxides of: silicon, iron, aluminum, sodi potassium, bismuth, magnesium, zircon
	Calcium phosphate
MTA (BioMTA+,Cerkamed, Stalowa Wola, Poland) Liquid	Purified water
	Calcium catalyst
MTA (NuSmileNeoMTA) Powder	Tricalcium Silicate
	Tantalite
	Dicalcium Silicate
	Calcium Sulfate
	Tricalcium Aluminate
MTA (NuSmileNeoMTA) Liquid	Water-based liquid
MTA Plus (Prevest Ltd., Jammu, India) Powder	Portland cement*
	Calcium sulphate
	Magnesium oxide
MTA Plus (Prevest Ltd., Jammu, India) Liquid	Hydrated polymer gel/Distilled wate
Biodentine (Septodont) powder	Calcium carbonate
	Zirconium dioxide
	Calcium oxide
